# Methodological validation and inter-site analysis in Late Bronze and Early Iron Age cremations using tooth cementum annulation counts

**DOI:** 10.1038/s41598-026-51841-z

**Published:** 2026-05-19

**Authors:** Agata Hałuszko, Stefan Tangl, Toni Dobsak, Fabian Kanz

**Affiliations:** 1 Institute of Archaeology, Maria Curie-Skłodowska University, Maria Curie-Skłodowska 4A Square, 20-031 Lublin, Poland; 2 Archeolodzy.org Foundation, Rynek 21/6, 58-100 Świdnica, Poland; 3 Karl Donath Laboratory for Hard Tissue and Biomaterial Research, University Clinic of Dentistry, Medical University of Vienna, Sensengasse 2a, 1090 Vienna, Austria; 4 Austrian Cluster for Tissue Regeneration, Donaueschingenstr 13, 1200 Vienna, Austria; 5 Center for Forensic Medicine, Medical University of Vienna, Sensengasse 2, 1090 Vienna, Austria

**Keywords:** Cementochronology, Cremation, Age estimation, Urnfield culture, Cremated human remains, Microscopy, Ageing, Anatomy, Biomarkers

## Abstract

Estimating age at death in archaeological populations is often limited by poor skeletal preservation, particularly in cremated remains. Tooth cementum annulation counts (TCAc), based on incremental lines of Salter (ILS), offer a promising alternative. In this study, TCAc is evaluated in terms of feasibility, microstructural preservation and internal consistency in cremated archaeological teeth, focusing on methodological applicability rather than chronological accuracy. We analysed 62 roots from eight Lusatian Urnfield culture cemeteries in southwestern Poland, dating to the Late Bronze and Early Iron Age. Undecalcified transverse sections were prepared, and ILS were examined alongside two complementary indicators: ILS width (ILSw) and the ratio of acellular extrinsic fibre cementum thickness to ILSw (AEFCt/ILSw). Results showed good intra- and inter-observer repeatability of ILSc, and both AEFCt and ILSc displayed significant age-related increases (*p* < 0.01). Combined estimates based on TCAc and AEFCt/ILSw yielded narrower age ranges than morphology alone. ILSw did not differ by sex, age group or tooth type, but varied significantly between cemeteries (*p* < 0.001), potentially reflecting environmental or life-history factors. These findings indicate that TCAc shows practical potential for use on cremated archaeological material, while also highlighting the need for future comparisons with known-age collections to refine interpretative standards.

**Supplementary Information:**

The online version contains supplementary material available at 10.1038/s41598-026-51841-z.

## Introduction

Estimation of the age at death of individuals from archaeological contexts is one of the most important parameters used in osteobiographical and population studies. A number of methods have been developed for estimating age at death^1,2^, the use of which depends on both the stage of skeletal development^3,4^ and, in the case of adults, on the degree of degeneration and maturation of osseous features^1^. In the case of burned and cremated material, age at death estimation of adults is most often carried out based on an assessment of the degree of obliteration of the cranial sutures^5,6^ as this is often one of the few observable age-related features in highly fragmented skeletal remains. However, the diagnostic value of this approach is limited, particularly when it is not possible to observe and compare the degree of obliteration on both the endocranial and ectocranial surfaces of the analysed suture segment and when other skeletal indicators are not preserved^5,7–9^.

The tooth cementum annulation counts (TCAc) technique, also called cementochronology^10,11^, or dental cementum increment analysis^12^ is one of the methods considered at least equally reliable for estimating age at death^13–15^(Supplementary section SI1 online), and even deemed by some to be more precise^16–18^. In theory, it is believed to be a simple method, but, in fact, it remains a biological ‘black box’, as its mechanisms of formation and growth are not yet fully understood^19–22^.

### TCAc studies of cremated teeth

Most of the studies on the applicability of the TCAc method to cremated teeth have been conducted on extracted modern teeth, burned under laboratory conditions^23–26^. In the case of cremated remains from archaeological contexts, to our best knowledge, cementochronology has only been conducted by Großkopf^23,27^ who analysed 10 teeth, including canines and premolars, from urn burials dating to the Pre-Roman Iron Age at the Schleswig-Holstein site in Germany. The research by Gocha and Schutkowski^25^ showed that identification of the acellular extrinsic fibre cementum (AEFC) was possible in 63.3% of the samples examined. The visibility and precise identification of the number of incremental lines of Salter (ILS) decreased as the temperature of the teeth burning under laboratory conditions increased^25,26^. In contrast, when examining teeth from archaeological contexts, histologically visible growth lines found in AEFC were surprisingly well visible and recognizable^23,27^. Researchers have linked these differences to the protective effect of soft tissue and alveolus surrounding the tooth root during cremation^23,26^.

When examining cremated teeth, it seems reasonable to analyse changes in the dimensions of the tooth and its respective tissues as well. Experiments carried out at 400 °C and 900 °C showed that the tooth root shortens by 3.4 ± 8.26% and 14.9 ± 9.68% respectively, the mesiodistal diameter by 4.01 ± 10.30% and 7.71 ± 7.71%, the buccolingual diameter by 2.59 ± 5.65% and 13.55 ± 10.42%^26^. At present, the influence of high temperatures on AEFC thickness (AEFCt), single incremental line of Salter width (ILSw), and the visibility or structural integrity of transparent and opaque bands remains unknown and requires further controlled investigation.

**Fig. 1:**
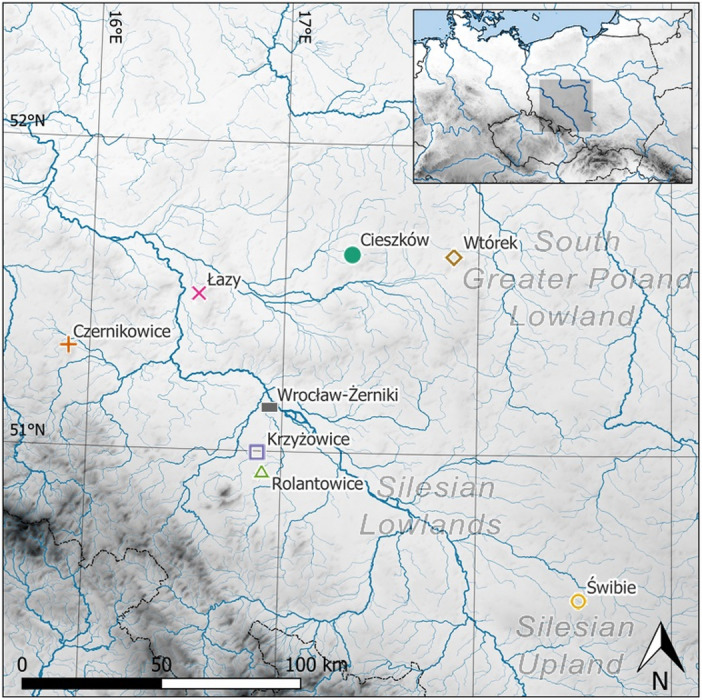
Spatial distribution of the archaeological cemeteries included in this study, all situated within the southwestern region of present-day Poland. Site symbols correspond to those used consistently throughout statistical and comparative visualizations. The map also delineates the three geographic zones referenced in subsequent analyses: South Greater Poland Lowland, Silesian Upland, and Silesian Lowlands. The map was created in QGIS 3.34 (https://qgis.org) using NASA Shuttle Radar Topography Mission (SRTM) open data distributed by OpenTopography (https://opentopography.org; 10.5069/G9445JDF; accessed 1.05.2025) and vector layers from the EU-Hydro River Network Database, Version 1.3, obtained from European Union’s Copernicus Land Monitoring Service (https://land.copernicus.eu/; 10.2909/393359a7-7ebd-4a52-80ac-1a18d5f3db9c; accessed 1.05.2025). Map prepared with the assistance of M. Mackiewicz.

The research focused on assessing the feasibility and internal consistency of TCAc in prehistoric cremated material, with particular attention to the observability and preservation of acellular extrinsic fibre cementum (AEFC) and to whether these structures can yield coherent age-related patterns within the assemblage. In the absence of individuals of known age, the research does not attempt to verify the accuracy of age at death estimation; in this context, “methodological validation” refers to assessing the practicability, reproducibility, and internal agreement of AEFCt and ILS counts (ILSc) measurements in cremated archaeological teeth. The condition of the material and its influence on the visibility and recognisability of ILS were considered as part of this methodological framework. The archaeological context imposes constraints independent of analytical design, including selective preservation of specific tooth types and variable microstructural integrity, necessitating a multi-level analytical approach. The study also sought to assess the repeatability of ILSc through intra- and inter-observer comparisons and to examine general correspondence between broad morphological age categories and age-related patterns inferred from AEFCt. Because the material derives from several cemeteries associated with Lusatian Urnfield culture (LUC) communities (Fig. 1; Table 1, Supplementary Fig. S1 and Table S1 online), the research additionally considered whether variation in AEFCt or ILSw occurs across sites, without assuming that such differences reflect any single underlying cause.

**Table 1:** Overview of cremated tooth root samples analysed in the study.

Site	Voivodeship	Geographical region	Number of examined individuals	Number of examined teeth	Number of sections	Number of sections with countable ILS	Mean thickness of the ground sections ± SD
Cieszków	Lower Silesia	South Greater Poland Lowland	1	1	3	3	51.50 ± 3.536
Czernikowice	Lower Silesia	Silesian Lowlands	3	5	24	11	40.59 ± 16.586
Krzyżowice	Lower Silesia	Silesian Lowlands	6	6	29	18	39.93 ± 7.181
Łazy	Lower Silesia	Silesian Lowlands	5	5	32	15	36.86 ± 10.569
Rolantowice	Lower Silesia	Silesian Lowlands	3	3	12	11	35.27 ± 14.718
Świbie	Silesia	Silesian Upland	27	27	121	106	34.23 ± 10.968
Wrocław-Żerniki	Lower Silesia	Silesian Lowlands	3	3	12	7	34.75 ±9.459
Wtórek	Greater Poland	South Greater Poland Lowland	12	12	59	31	36.02 ± 11.099

## Results

### Sample overview and slide preparation

TCAc analyses were performed on a total of 292 slides obtained from 62 teeth from 59 separate burials (Supplementary Fig. S2 and Tables S2–S4 online). For 202 of them, ILS was observed and counted, and for 236, data on AEFCt were obtained. Moreover, for 56 slides, AEFC observations were not feasible, primarily due to the presence of cellular intrinsic fibre cementum (CIFC) and cellular mixed stratified cementum (CMSC) in the apical and middle parts of the roots, where these cellular forms predominated over AEFC. Across the assemblage, countable ILS sections were obtained from multiple cemeteries and represented a range of morphological age categories (Supplementary Table S2 online). For most sites this distribution was broadly comparable, with two exceptions: Cieszków is represented by a single analysed tooth (three sections), and the Czernikowice sample derives mainly from children. For two teeth, AEFC microscopic observations proved impossible. The TCAc method could not be applied to a female premolar – F? of Adultus/Maturus age category from grave 458 at Świbie due to the exclusive presence of CIFC. Another tooth (Czernikowice, grave 74, Infans II, FDI 12/22) dissolved for unknown reasons during embedding in resin (Supplementary Fig. S6 online), with only a small root fragment retaining identifiable structure. A deciduous tooth from grave 22 (same site) showed partial degradation limited to the cervical root (Supplementary Fig. S7 online). Replacement samples were analysed (Supplementary Table S2 online); while one yielded usable results, another (FDI 31/41) fragmented during preparation. Cementochronological analysis was still possible, and dentin mineralization defects, including interglobular dentin (IGD) and contour lines of Owen, were observed^28^.

### Error estimation: intra- and inter-observer repeatability

The intra-observer error of ILSc was assessed for 60 teeth (excluding two as noted above). Repeated counts performed by Observer 1, separated by a six-month interval, showed a low absolute measurement error (TEM = 2.20; rTEM = 12.3%; ICC = 0.92), indicating strong internal consistency. A paired *t*-test revealed a statistically significant difference between the first and second counts (*t* = 7.38, *p* < 0.001), but the absolute magnitude of this difference was small (≈ 2 ILSc), indicating that it was not of practical significance.

The inter-observer error was evaluated for 44 teeth for which at least three slides with countable ILS were available. Comparison between the mean ILSc of Observer 1 and independent counts by Observer 2 yielded a similarly low measurement error (TEM = 2.28; rTEM = 12.9%; ICC = 0.91). No significant systematic bias was detected (*t* = 1.53, *p* = 0.13), confirming high inter-observer consistency. Although rTEM values exceeded the conventional 5% threshold, the absolute discrepancies remained minimal, averaging approximately 2 ILSc and corresponding to estimated age differences of about 2 years. These results are consistent with previously published data^19,25^, supporting the reliability of the ILSc counts for further analysis.

### Analyses across tooth types

For the comparison by tooth type: incisors, canines, premolars, first molars, molars (X6/X7), and deciduous molars, a Kruskal-Wallis test showed significant differences for AEFCt (*H* = 16.68, *p* = 0.0051, η² = 0.29) and ILSc (*H* = 12.48, *p* = 0.0288, η² = 0.22), while no significant effect was found for ILSw (*H* = 10.58, *p* = 0.0604, η² = 0.19). However, no pairwise post-hoc comparison remained significant after Holm correction. After excluding deciduous molars and third molars, only AEFCt remained significantly differentiated (*H* = 10.29, *p* = 0.0359, η² = 0.19), yet again without significant post-hoc contrasts.

The teeth originally labelled as X6/X7 (Supplementary Table S2 online) displayed higher AEFCt and ILSc values than first molars, suggesting they are more likely to represent first molars; however, to maintain a conservative approach, an averaged eruption age for both first and second molars was applied.

The observed variation in AEFCt and ILSc is therefore likely age-related rather than tooth-type specific. In contrast, ILSw remained comparatively stable, indicating limited sensitivity to tooth morphology within this assemblage.

### Analysis of tooth root regions

**Fig. 2:**
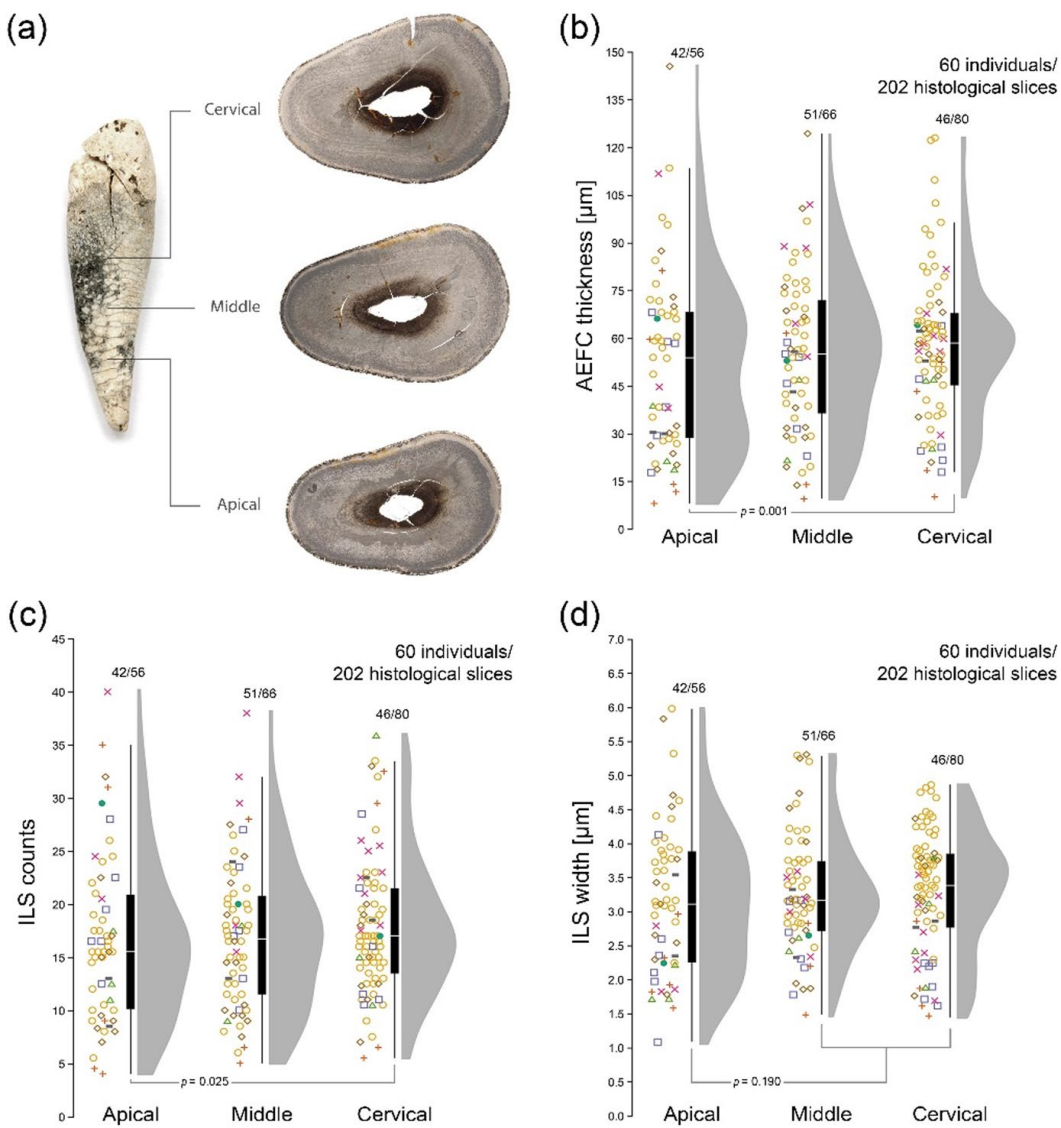
Variation in acellular extrinsic fiber cementum thickness (AEFCt) and incremental lines of Salter (ILS) characteristics across tooth root regions in cremated human teeth. (**a**) Representative cremated tooth showing the apical, middle, and cervical thirds of the root; (**b**) distribution of acellular extrinsic fiber cementum thickness (AEFCt) across root regions presented as half-violin, box, and jitter plots; (**c**) distribution of incremental line of Salter counts (ILSc) by root region; (**d**) distribution of incremental line of Salter width (ILSw) by root region.

Analyses based on simple group comparisons across tooth root thirds (apical, middle and cervical) (Fig. 2a) revealed no statistically significant differences in AEFCt (*p* = 0.4134), ILSc (*p* = 0.4662) or ILSw (*p* = 0.3686) (Fig. 2b-d and Supplementary Table S5 online).

To account for repeated measurements within teeth and the unequal representation of root regions, we fitted linear mixed-effects models with tooth identity as a random intercept and root region (apical, middle, cervical) as a fixed factor. Only teeth that contributed measurements from at least two distinct root regions were included. The overall effect of root region was statistically significant for AEFCt (likelihood-ratio test: *chi²* = 20.20, *p* < 0.001, η²₍approx₎ ≈ 0.12) and for ILSc (*chi²* = 7.36, *p* = 0.025, η²₍approx₎ ≈ 0.04), indicating detectable but relatively small variation among root regions. Post-hoc fixed-effect comparisons (estimated marginal means) showed that cervical values were significantly lower than apical values for both AEFCt (β = −8.45 μm, SE = 2.46, 95% CI: −13.27 to − 3.63, *p* = 0.001) and ILSc (β = −4.65, SE = 1.95, 95% CI: −8.47 to − 0.84, *p* = 0.017), whereas no statistically significant differences were detected between the middle and apical regions for either variable. In contrast, no significant effect of root region was observed for ILSw (*chi²* = 3.32, *p* = 0.190, η²₍approx₎ ≈ 0.02), suggesting that ILSw remains largely stable across the examined tooth root regions.

Taken together, these findings indicate that variation in AEFCt and ILSc across tooth root regions is small relative to biological variance and does not compromise the stability of AEFCt/ILSw-based age extrapolation, which appears robust and unbiased with respect to sampling location along the root.

### Correlation with morphological age

**Fig. 3:**
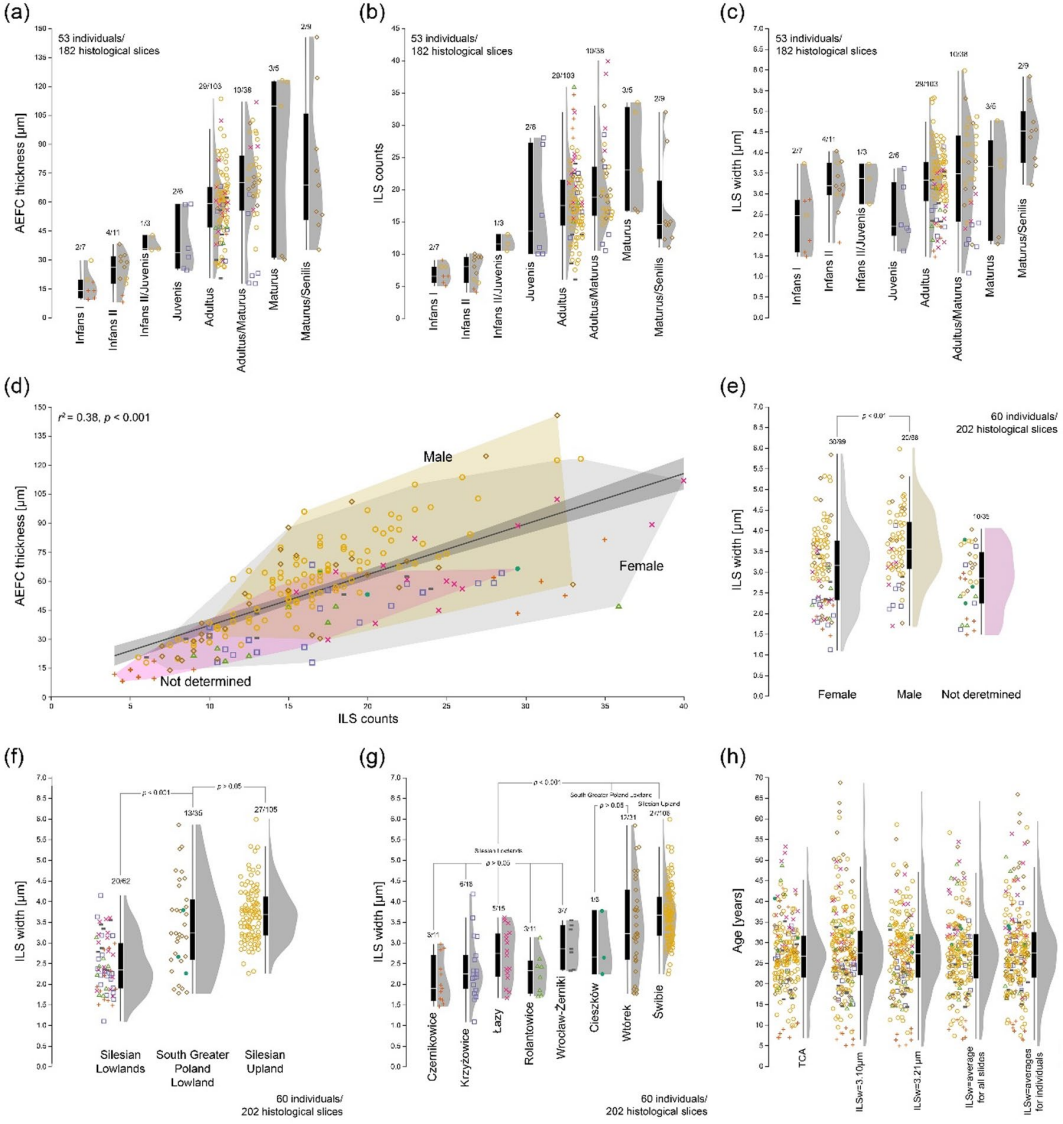
Variation in acellular extrinsic fiber cementum thickness (AEFCt), incremental line of Salter counts (ILSc), and line width (ILSw) by age, sex, and geographic context based on tooth cementum annulation counts (TCAc) analysis. (**a**) AEFC thickness stratified by morphological age category; (**b**) counts of incremental lines of Salter (ILSc) by morphological age category; (**c**) incremental lines of Salter width (ILSw) distribution across morphological age categories; (**d**) linear regression depicting the relationship between AEFC thickness and ILS count, with stratification by morphological sex; (**e**) variation in incremental lines of Salter width (ILSw) according to morphological sex; (**f**) ILS width differences among geographic regions; (**g**) ILS width comparison across cemeteries; (**h**) comparison of individual age at death estimates derived from TCAc (ILS counts combined with the age of tooth eruption) and the AEFC thickness to ILS width (AEFCt/ILSw) ratio, presented using half-violin and box plots with jittered points.

Examination of AEFCt revealed statistically significant differences between age categories estimated by morphological methods (*H* = 19.68, *p* = 0.0063, η² = 0.282), suggesting age-related variation in this parameter (Fig. 3a and Supplementary Table S6 online). These morphological categories should be understood as operational biological groupings derived from preserved skeletal traits rather than direct equivalents of chronological age, and are therefore treated primarily as comparative reference variables in relation to cementum-based results (Supplementary Table S3 online). Post-hoc pairwise comparisons using the Mann-Whitney U test with Holm adjustment indicated that individuals classified as Infans I and Infans II exhibited significantly lower AEFCt values compared with those classified as Adultus (adjusted *p* < 0.05), whereas no other pairwise differences remained statistically significant after correction for multiple testing. Following the exclusion of subadult age categories, no statistically significant differences in AEFCt were detected among the remaining adult age groups (*H* = 1.524, *p* = 0.6768), indicating that the previously observed variation was primarily driven by the youngest age categories. This outcome is also likely influenced by the composition of the sample, which was dominated by individuals assigned to the Adultus category. Consistently, a linear mixed-effects model incorporating repeated measurements likewise revealed no statistically significant age-related differences in AEFCt, further supporting the inference that cementum thickness does not systematically differentiate between adult age categories in this dataset.

The statistical evaluation of ILSc showed significant differences across age categories (*H* = 21.33, *p* = 0.0033), consistent with an age-related increase in this parameter (Supplementary Table S7 online). Post-hoc pairwise comparisons (Mann-Whitney U tests with Holm adjustment) indicated that ILSc was significantly lower in Infans I and Infans II compared with Adultus category (adjusted *p* < 0.05), whereas no other pairwise differences reached statistical significance. When the subadult categories were excluded, the differences were no longer statistically significant (*H* = 2.57, *p* = 0.4622) (Fig. 3b and Supplementary Table S7 online). Nonetheless, a linear mixed-effects model that accounted for repeated measurements within teeth indicated significant variation among adult age categories (likelihood-ratio test: *chi²* = 18.58, *p* < 0.001, η²₍approx₎ ≈ 0.10). This result indicates that age-related differences in ILSc among adults are biologically meaningful, although additional factors must also contribute to the observed variability. Post-hoc pairwise comparisons with Bonferroni correction showed that individuals classified as Maturus had significantly higher ILSc values than those classified as Adult (adjusted *p* < 0.05), whereas all other contrasts were not statistically significant. Interestingly, two individuals morphologically assigned to the Maturus/Senilis category displayed a lower-than-expected number of ILSc despite exhibiting the highest AEFCt values in the sample (Supplementary Table S6 and S7 online). This discrepancy indicates that ILSc and AEFCt may not consistently follow parallel age-related trajectories, implying that the two measures may become partially decoupled in certain individuals belonging to the oldest age categories. At present, the underlying causes cannot be determined from the available data and may reflect inter-individual biological variation rather than a single explanatory factor (Fig. 3b and Supplementary Table S2 online).

At the same time, no statistically significant differences in ILSw were observed across morphological age categories (Kruskal-Wallis test: *H* = 9.76, *p* = 0.2026), indicating that ILSw does not vary systematically with advancing age in the examined material (Fig. 3c; Supplementary Table S8 online). To verify this result while accounting for repeated measurements within teeth, a linear mixed-effects model was fitted with age category as a fixed factor and tooth transverse sections as a random intercept. The model similarly provided no evidence for age-related differences in ILSw (all fixed-effect contrasts: *p* > 0.40), suggesting that this parameter remains relatively stable across the full range of recorded age categories, and that inter-individual variation exceeds any detectable age-related trend.

The ILSc was also positively correlated with AEFCt (Pearson’s *r* = 0.62, *r²* = 0.38, *p* = 8.6E-24), indicating that approximately 38% of the variability in ILSc is explained by variation in cementum thickness (Fig. 3d). Despite this overall directly proportional relationship between AEFCt and ILSc, visual inspection of the regression plot suggested noticeable inter-sex and/or inter-population heterogeneity in the data (Fig. 3d).

### Sex-based differences in cementum microstructure

Given the above observations, and considering the association between AEFCt and sex suggested by other authors^29^, statistical analyses were carried out with separation of adult female and male individuals (Supplementary Tables S2 and S4 online). Based on all 167 slide-level measurements, ILSw values were significantly higher in males (mean = 3.61 μm, 95% CI: 3.40–3.81) than in females (mean = 3.16 μm, 95% CI: 2.97–3.35), with a mean difference of 0.44 μm (95% CI: 0.16–0.73). Welch’s unequal-variance t-test confirmed that this difference was statistically significant (*t* = 3.13, *p* = 0.00206), which was further supported by permutation testing (*p* = 0.0027) and Bayesian inference (BF = 12.23, indicating strong evidence for unequal means). The effect size was small (Cohen’s *d* = 0.48), suggesting that although males exhibit measurably wider ILSw on average than females, sex accounts for only a limited proportion of the observed variability (Supplementary Table S9 online).

When per-individual mean values were analysed, no statistically significant sex-related difference was observed (Fig. 3e), unlike in the full dataset, which was dominated by material from the Świbie site where ILSw values were generally higher (Fig. 3d). To additionally evaluate whether this pattern persisted when accounting for repeated measurements within teeth, we fitted a linear mixed-effects model with sex as a fixed effect and tooth transverse section identity as a random intercept. The model indicated no statistically significant association between sex and ILSw (β = 0.575 μm, SE = 0.428, *z* = 1.35, *p* = 0.179), showing only a non-significant trend towards higher values in males. This suggests that the apparent sex-related pattern observed in the full section-level dataset was driven primarily by inter-site differences rather than sex itself. We therefore assessed inter-population and regional variation to determine the main source of ILSw differences.

### Inter-population and regional variability

A Kruskal-Wallis test demonstrated statistically significant variation in ILSw between cemeteries (*H* = 34.46, *p* = 5.47E-06). Dunn post-hoc comparisons (Holm-adjusted) showed that individuals from Świbie and Wtórek exhibited significantly higher ILSw values than those from all other analysed cemeteries (*p* < 0.01), while no significant difference was detected between Świbie and Wtórek (*p* > 0.05), and no further pairwise contrasts reached significance. To complement these findings and account for repeated measurements per individual, we additionally applied a linear mixed-effects model to the full dataset, which likewise indicated significant between-cemetery variation in ILSw (*chi²* = 26.91, *df* = 6, *p* < 0.001, η²₍approx₎ ≈ 0.12). The highest ILSw values calculated from data for all 202 slides were recorded for the individuals from the cemetery in Świbie – mean of 3.65 μm and Wtórek – mean of 3.35 μm (Table 2). The average ILSw for the other cemeteries ranged from 2.06 μm to 2.94 μm. According to the averaged data for 60 individuals, the mean ILSw for the specimens from the Świbie site was 3.58 μm, while for Wtórek, it was 3.26 μm. The mean ILSw for the other cemeteries ranged from 2.16 μm to 2.91 μm (Table 3). The overall mean ILSw was 3.21 μm when calculated from 202 slides and 3.10 μm in case of the averages of 60 individual specimens (Tables 2 and 3). Post-hoc Tukey comparisons based on the full dataset confirmed significantly higher values in Świbie relative to several other sites (*p* < 0.05), with effect sizes indicating large differences (*d* = 0.83–0.96), moderate differences for Łazy and Wrocław-Żerniki (*d* = 0.51–0.68), and a small difference relative to Wtórek (*d* = 0.26). These findings indicate a non-uniform inter-population pattern of ILSw, suggestive of underlying developmental and/or environmental variation, which may be influenced by diet or other environmentally mediated factors.

**Table 2:** Cemetery-specific incremental line of Salter (ILSw) widths based on mean measurements from 202 histological slides.

ILSw [µm]	Cieszków	Czernikowice	Krzyżowice	Łazy	Rolantowice	Świbie	Wtórek	Wrocław-Żerniki	Total
N	3	11	18	15	11	106	31	7	202
Min	2.25	1.47	1.09	1.7	1.72	0	1.77	2.33	1.09
Max	3.78	2.97	3.62	3.59	3.13	5.98	5.84	3.54	5.98
Median	2.65	1.87	2.225	2.4	2.25	3.665	3.22	2.86	3.20
Mean	2.89	2.06	2.28	2.62	2.30	3.65	3.35	2.94	3.21
SD	0.793	0.573	0.611	0.668	0.437	0.759	1.064	0.501	0.915

**Table 3:** Cemetery-specific incremental line of Salter (ILSw) widths based on mean measurements from 60 teeth.

ILSw [µm]	Cieszków	Czernikowice	Krzyżowice	Łazy	Rolantowice	Świbie	Wtórek	Wrocław-Żerniki	Total
N	1	4	6	5	3	26	12	3	60
Min	2.89	1.91	1.62	2.46	1.89	2.72	1.87	2.81	1.62
Max	-	2.40	2.66	3.14	2.66	4.21	4.21	3.05	4.21
Median	-	2.17	2.39	2.57	2.08	3.58	3.37	2.86	3.16
Mean	-	2.16	2.28	2.65	2.21	3.58	3.26	2.91	3.10
SD	-	0.251	0.373	0.280	0.401	0.390	0.698	0.127	0.689

A separate Kruskal-Wallis test for geographical regions also indicated a highly significant effect (*H* = 36.56, *p* = 1.141E-08), with Dunn-corrected comparisons confirming that both the Silesian Upland and the South Greater Poland Lowland differed significantly from the Silesian Lowlands (*p* < 0.001), whereas their mutual contrast remained non-significant. The variation in ILSw was also related to the geographical region from which the individuals originated (Figs. 1 and 3f and g and Supplementary Table S10 and S11 online), and this relationship was statistically significant, as confirmed by a linear mixed-effects model incorporating repeated measurements from transverse root sections as a random factor (*chi²* = 21.12, *p* < 0.001).

### TCA age estimation and its correlation with other parameters

The TCA age of individuals was calculated based on the sum of the age of eruption and the number of ILS for the tooth analyzed, as well as the sum of the age of eruption and the number of ILS calculated based on the quotient of the AEFCt and the computed four different widths of a single ILS: (a) 3.21 μm (mean of 202 slides; Table 2), (b) 3.10 μm (mean of 60 individuals; Table 3) and according to site-specific averages based on (c) all slides (Table 2) and (d) averages for individuals (Table 3). No statistically significant differences were observed in the averages of the ages calculated by the TCA technique and AEFCt/ILSw ratio (Fig. 3h and Supplementary Table S2 online).

**Fig. 4:**
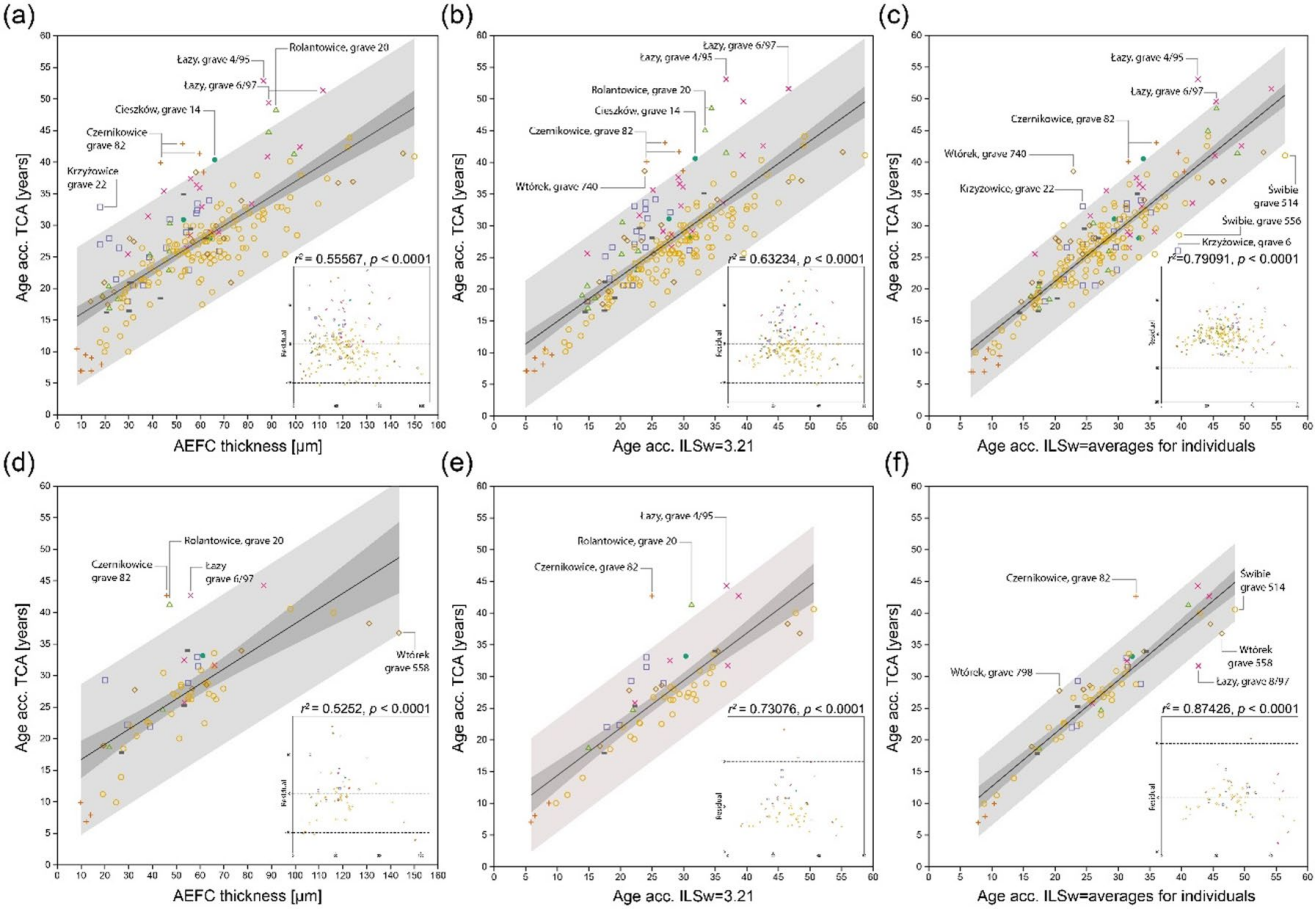
Regression models and residuals illustrating the relationship between age at death estimates derived using tooth cementun annulation counts (TCAc) method and the acellular extrinsic fiber cementum thickness to incremental line of Salter width (AEFCt/ILSw) ratio. (**a**) TCAc-derived age estimates plotted against the acellular extrinsic fiber cementum thickness (AEFCt) for 202 histological slides; (**b**) age estimates based on the AEFCt/ILSw ratio, using a fixed incremental line of Salter width (ILSw) of 3.21 μm, for the same dataset; (**c**) cemetery-specific AEFCt/ILSw age estimates for 202 slides, with ILS width values as presented in Table 2; (**d**) TCAc-derived age estimates versus the acellular extrinsic fiber cementum thickness (AEFCt) for a subset of 60 individual teeth; (**e**) AEFCt/ILSw age estimates for 60 teeth using the fixed incremental line of Salter width (ILSw) of 3.21 μm; (**f**) cemetery-specific AEFCt/ILSw age estimates for 60 teeth, using incremental line of Salter width (ILSw) values as listed in Table 3.

The TCA age calculated on the basis of 202 slides (Fig. 4a-c), was positively correlated with the AEFCt (*r*^*2*^ = 0.55567, *p =* 1.73E-38), the TCA age calculated with the ILSw of 3.21 μm (*r*^*2*^ = 0.63234, *p =* 2.0036E-46), the TCA age calculated with the ILS width of 3.10 μm (*r*^*2*^ = 0.63163, *p* = 2.4461E-46), and the TCA age calculated with the ILSw computed for particular cemeteries on the basis of all slides (*r*^*2*^ = 0.63191, *p =* 2.2631E-46; Table 2), as well as when the mean values for individuals was taken into account (*r*^*2*^ = 0.79091, *p =* 6.7667E-70; Table 3).

Similar data were obtained for the TCA age averaged for 60 individuals (Fig. 4d-f), with the highest correlation obtained between the TCA age and age according to ILSw obtained as averages for individuals (*r*^*2*^ = 0.87426, *p =* 7.7657E-28; Table 3; Fig. 4f). The age of most individuals calculated by the TCA technique and the AEFCt/ILSw ratio method corresponded with the age categories determined by morphological methods. According to the data obtained, the number of ILS was higher in relation to the AEFCt in two female individuals – 3.23% (Supplementary Table S2 online): Czernikowice, grave 82 and Rolantowice, grave 20, resulting in overestimation of TCAc age by approximately one to two decades (Fig. 4d-f, Supplementary Table S2 online). It is likely that in the case of these individuals, we registered the presence of the phenomenon of multiplication of the number of ILS.

## Discussion

Estimating the age at death of cremated individuals based on the TCAc technique and AEFCt measurements appears to be a promising research procedure, although it is still rarely used^20,30^. However, during the analyses presented here, we encountered several issues that were not always predictable. In this study, one tooth “dissolved”, while two others became partially dissolved and fragmented during embedding in MMA-based resin (Supplementary Fig. S6 and S7 online). It is possible that dehydration in ethanol prior to embedding contributed to the disintegration of the dental tissue. Additionally, differences in the consistency of soil and the presence of water in the burial environment may have affected the preservation state of the teeth and should be considered as a potential factor influencing sample stability.

Previous studies have demonstrated that the choice of embedding resin, direct epoxy vs. MMA, affects the contrast and visibility of AEFC bands^31–33^. In this context, the use of MMA-based resin was crucial, as it not only enabled the preparation of very thin sections required for reliable microscopic observation and measurement, but also penetrated and stabilised internal cracks and micro-fissures within the specimen, thereby preventing further structural damage during cutting and grinding^31^. At the same time, we acknowledge that it cannot be ruled out that the use of MMA may have influenced the visibility or clarity of ILS. However, several specimens displayed very well-defined bands, which suggests that other factors related to preservation conditions, thermal alteration or post-depositional processes may have played a more substantial role in determining their visibility.

In the case of the individual whose tooth showed complete dissolution, histological examination of an additional tooth revealed interglobular dentine (IGD), and earlier anthropological assessment identified palaeopathological changes including diploë hypertrophy and porotic hyperostosis of the parietal bones^34^. Although palaeopathological evaluation of cremated remains is particularly challenging, the observed “dissolution” effect in tooth FDI 12/22 may tentatively be associated with metabolic disturbances affecting mineralisation of dental tissues rather than solely with embedding procedures or thermal alteration^35^.

Research on cremated teeth from archaeological contexts remains scarce, and no systematic investigations have yet defined the optimal thickness of undecalcified embedded tissue sections. The slides’ thickness determined from the laboratory-burned teeth analyses was around 50–60 μm^25^ and around 100 μm for Pre-Roman Iron Age archaeological samples^27^. Most TCAc studies on inhumed/extracted teeth are based on slides with a thickness of ca. 70–100 μm^18,26,36,37^, but also ca. 50–60 μm^24^. Meanwhile, this study demonstrated that the optimal thickness of undecalcified embedded tissue sections from cremated teeth was lower than expected, ranging from 22.5 to 65.0 μm, with an average of 36.1 μm. The section thickness was individually adjusted based on the transparency of the tissue to ensure optimal visibility of microstructures (Supplementary Fig. S8 online). However, the variability in optimal thickness between individual teeth precluded the use of automated grinding procedures, thereby extending the duration of this already time-consuming preparation process. Importantly, this approach does not correspond to the standard protocol typically used in TCAc studies, but cremated teeth, unlike inhumed ones, do not exhibit sufficient translucency and therefore do not produce the overlapping-layer effect that normally enables the use of uniform, thinner sectioning.

Despite previous concerns regarding the repeatability of ILSc^36,38,39^, our results indicate satisfactory repeatability. However, the influence of different embedding resins on band visibility^31^ requires further investigation, as it may reduce intra- and inter-observer variability. Using phase-contrast and polarized light microscopy could perhaps also increase the identifiability of ILS^40^.

In contrast to human skeletal remains, one of the troublesome aspects of implementing the TCAc procedure was the correct odontological identification of the tooth root fragment subject to analysis, which translated directly into the estimated age of eruption of the tooth. In this study, most cremated tooth fragments were identified outside the alveoli and the burials often contained commingled remains. To ensure confident attribution, only teeth assignable to specific individuals were included, resulting in a final sample of 60 individuals; therefore, prior anthropological analysis was necessary to determine the minimum number of individuals and assess the feasibility of tooth-to-individual assignment. For most individuals, TCAc results aligned with the morphological age categories while markedly narrowing the estimated age span. It must be emphasised that age estimations obtained through the TCAc and AEFCt/ILSw methods could not be verified against calendar age, as reference individuals of known age are unavailable in prehistoric assemblages. Consequently, the present study assesses methodological feasibility and internal consistency rather than chronological accuracy. Studies conducted on laboratory-burned teeth sampled from individuals with known calendar ages confirm the high relevance of the use of the TCAc technique^23,24,26^, although at the same time showed a slight underestimation of the age of individuals^23,25^. In this study, we were only able to base the analyses on the comparison of ILSc with AEFCt and AEFCt/ILSw ratio, which provided results matching those of the morphognostic methods for most individuals. Furthermore, due to the lower identifiability of ILS in cremated tooth slides compared to inhumed teeth, it is a stimulating perspective to conduct studies on a larger group of individuals rather than a single sample, as this may translate into the possibility of age estimation based on the AEFCt/ILSw ratio.

Conducting cementochronological studies on a larger group of individuals also makes it possible to identify multiplication effects in the number of ILS. In our sample, two cases of ILS multiplication were identified. Both specimens belonged to individuals assessed as female, although the limited sample size precludes any sex-related interpretation of this pattern. This phenomenon has been previously reported in human cementum, typically with a frequency of approximately 3%^25,41^ or higher in some studies^42^. Although it has not yet been examined in detail, existing reports indicate that multiplication may take the form of a full doubling of the total number of ILS^41,42^. In contrast, the individuals in our study exhibit only a partial multiplication, affecting a limited number of increments, a pattern also noted in earlier research^25^. After applying the age extrapolation based on AEFCt/ILSw, the estimated TCAc age decreased by nearly 20 years for the individual from Czernikowice (grave 82) and by approximately 10 years for the individual from Rolantowice (grave 20). For the Czernikowice individual, this pattern is compatible with a probable full doubling of ILSc, whereas in the case of the Rolantowice individual the evidence suggests a partial multiplication rather than a complete duplication.

The phenomenon of partial multiplication has been identified far more frequently in tooth samples from various animal species and in individuals of both sexes^43–47^. Although its underlying causes remain uncertain, it has most often been linked to physiological cycles such as oestrus or winter dormancy^43^. Whatever its origin, this represents an important confounding factor that future studies should take into account.

Recently, there have been studies that have shown a relationship between AEFCt and sex^29,48^. In our study, the influence of sex on AEFCt and ILSw appears to be obscured by inter-population variation, and the differences initially observed are likely to reflect inter-site factors rather than a consistent sex-related effect across the examined assemblage. Notably, when repeated measurements were accounted for in the statistical analyses, these differences were no longer evident, suggesting that factors other than sex may be driving the observed variability. These results should be interpreted cautiously, particularly in light of the limited sample size and the inherent uncertainty of sex assessment in archaeological cremation contexts. The fragmentary and thermally altered nature of prehistoric cremated remains considerably restricts the preservation of sexually dimorphic traits, increasing the risk of misclassification, even when individuals with the most diagnostically informative skeletal elements are selected. Consequently, the possibility of erroneous sex estimation cannot be fully excluded and may have contributed to the observed pattern.

This study was also not aimed at identifying ILS irregularities that may indicate multifactorial physiological stress^49,50^. However, our observations led to the identification of statistically significant differences in the ILSw of populations living in distinct regions of Poland. Similar studies have not been conducted before, so the interpretation of these results has to be conducted on multiple levels. These differences may reflect ecological factors and population-specific dietary regimes influenced by local environments, potentially involving increased masticatory stress from harder foods^29^, although additional explanations should be considered. However, without direct palaeodietary or physiochemical data for the cremated material, these interpretations should be regarded as preliminary, and alternative explanations including taphonomic or developmental factors cannot be excluded.

The only data on the palaeodiet of individuals associated with the LUC communities come from biritiual cemeteries, including, but not limited to, Świbie^51,52^ and Opole-Groszowice^53,54^. Stable isotope analyses of carbon (δ^13^C) and nitrogen (δ^15^N) carried out on 23 individuals from inhumation burials show that the paleodiet of these individuals was based on the food of terrestrial origin with a predominance of C_3_-type photosynthetic plants and a high proportion of animal-based foods^55^. Similar studies conducted on the LUC populations from cemeteries further to the east in Poland, on the other hand, showed a higher proportion of plants with C_4_/CAM photosynthesis in the diet^56,57^. However, no comparative studies comprehensively describe the effect of dietary intake on the changes in the AEFCt and ILSw.

Previously, statistically significant differences in the AEFCt between the mesial and distal surfaces of teeth were demonstrated, explaining this phenomenon by tensile forces resulting from mesial displacement^58^. Variation in the AEFCt was also observed within maxillary and mandibular premolars^59^. However, few inter-population studies were conducted in this regard^60^. It is also difficult to determine whether the diet of the analysed individuals differed substantially, and the cremation process precludes isotopic analyses. Importantly, we did not examine cementum thickness comparatively across different root surfaces; therefore, our results relate solely to the oral and vestibular aspects, which are generally considered less influenced by masticatory forces^29^.

Inter-regional differences in ILSw values were recorded^61^. In the case of the cemetery in Czernikowice, the lowest ILSw values were observed (Tables 2 and 3). Previous archaeological and palaeodemographic interpretations describe this group as representing a community of relatively low socio-economic status^34^, and the assemblage includes several young individuals. Recorded defects in dentin mineralisation were also noted in this group (Supplementary Fig. S6 and S7 online). However, while such changes may be consistent with various developmental or metabolic disturbances, the present dataset does not allow determination of their underlying causes or potential relationship to ILSw. Therefore, the interpretation of ILSw variability must remain cautious, and no causal inference should be drawn from the current material.

Another factor that could perhaps have influenced the variation of the ILSw is the technique of cremating the remains, the construction of the pyre, and a range of other funerary elements, which are unverifiable in this study. However, no differences in the colour of the studied teeth and other skeletal bones were noticed between the sites (Supplementary Fig. S2-S5 online), which could indicate significant differences in the temperature or diverse techniques of cremating the remains. Although this study was not designed to directly test the effect of cremation on AEFCt or ILSw, the results indicate that AEFCt varied between different parts of the tooth root, whereas ILSw did not show such intra-root variability. This may suggest that cremation-related thermal alteration did not substantially modify AEFCt, and that ILSw exhibited stable values across the different parts of the tooth root.

Studies conducted by other researchers similarly indicate that ILSw values obtained from cremated specimens do not differ markedly from those recorded in non-archaeological samples and appear to vary primarily between populations rather than within individuals^19,26^. Oliveira-Santos and co-authors conducted a study on laboratory-burned teeth obtained from patients of Portuguese clinics. They subjected 42 teeth to combustion at temperatures of 400 and 900 °C, 21 teeth at each temperature. According to their results, the ILSw of teeth burnt at 400 °C was 3.29 μm on average, with a median of 3.20 μm, while at 900 °C the result was 3.57 μm on average, with a median of 3.50 μm^26^. With the assumption that increasing the temperature positively affects the shrinkage of the AEFC, the expected result would be a lower value of the ILSw in the case of the teeth burnt at higher temperatures. Meanwhile, the results obtained are the opposite. Taking into account this study, it must be assumed that any shrinkage of the AEFC is affected by temperatures of up to 400 °C and that increasing the temperature does not contribute to further changes in the AEFCt. The materials analysed in this study exhibit predominantly white to cream coloration, a feature generally associated with full calcination of bone, together with cracking and deformation indicative of exposure to elevated temperatures^34,62–68^. Although the precise thermal conditions cannot be reconstructed in detail, and no analyses specifically targeting thermal parameters were undertaken in this study, these macroscopic characteristics suggest that the cremation processes applied across the assemblage were broadly comparable in intensity. On this basis, the inter-site variation observed in ILSw is unlikely to result solely from differences in burning conditions. Nevertheless, at the current stage of research, the underlying causes of this variation cannot be determined unambiguously. Additional factors, including biological, depositional or taphonomic influences, may also have contributed, but confirming their impact would require further specialised analyses.

Although multiple biological and histomorphometric parameters could be assessed for the analysed material, the study is based on a relatively small sample size, and the results should therefore be interpreted with caution. The dataset represents only a subset of the variation that may have existed within and between prehistoric populations, underscoring the need for broader comparative research when applying TCAc to archaeological contexts. Future work should draw on larger, independently sourced samples and employ integrated analytical frameworks in which histological observations are systematically compared with isotopic, palaeopathological and environmental data. Unpacking the ‘black box’ of cementochronology thus remains an ongoing process that advances future research rather than yielding definitive conclusions at this stage.

Taken together, the results suggest that although TCAc does not yet permit calendar-age estimation in prehistoric cremated assemblages, it provides internally consistent quantitative proxies that can inform demographic reconstructions at the population level.

## Materials and methods

All research procedures were carried out in accordance with Polish national legislation concerning human remains from archaeological contexts, specifically following the Act of 23 July 2003 on the Protection and Care of Monuments (Dz.U.2024.1292)^69^ and the Act of 31 January 1959 on Cemeteries and Burial of the Dead (Dz.U.1959.11.62)^70^. In Poland, human remains from archaeological sites are regulated under these laws as cultural heritage and are not subject to other specific ethical regulations. All analyses were carried out with the permission of the institutions curating the archaeological collections, as detailed in the Ethics Declaration section. The study was additionally conducted in line with the ethical guidelines of the Polish Anthropological Society^71^ concerning the scientific treatment of archaeological human remains.

This research was conducted on 62 cremated teeth, from which 292 slides were obtained for further analysis. The teeth came from 60 individuals identified at eight archaeological sites associated with the LUC cemeteries dated to the Late Bronze Age and Early Iron Age, i.e. c. 1100 − 450 BC (Table 1, Supplementary section SI2, Fig. S1, S3-S5 and Table S1 online).

Age at death estimation followed standard osteological procedures^3,5,72–74^, and the study included nine subadults: two Infans I, four Infans II, one Infans II/Juvenis, two Juvenis, as well as 49 adults: 29 Adultus, 10 Adultus/Maturus, three Maturus, two Maturus/Senilis, and five individuals generally defined as adult. It was impossible to estimate the age at death for two individuals due to the lack of diagnostic bone fragments (Supplementary section SI3 and Tables S2–S3 online).

Among the materials analysed, there were 30 females, 20 males and 10 individuals of undetermined sex (Supplementary section SI3 and Tables S2–S4 online). It should be emphasised that sex estimation in cremated assemblages is inherently limited; however, the individuals included in this study displayed at least several diagnostic traits that allowed us to assign sex with reasonable caution. In further studies, having in mind the likely link between various abnormalities in the AEFC, the sex of individuals was included with caution on account of the separate issue of determining and confidence in assessing the sex of cremated individuals^5,72^.

The teeth examined were preserved as tooth roots, numbered using the FDI system^75^. The teeth analysed included eight incisors, 16 canines, 23 premolars, 12 molars, and three deciduous molars. Single-rooted teeth, primarily premolars, were preferentially selected for histological examination; however, the condition of the cremated prehistoric material often required the use of preserved root fragments, including those originating from molars, as no alternative preserved roots were available in the form of sufficiently large and morphologically recognisable fragments suitable for further TCAc analysis (Supplementary section SI4, Fig. S2 online).

Most of the teeth derived from burials of single individuals, including cases where each individual was placed in a separate urn with no evidence of commingling. Teeth from 25 individuals were selected from contexts containing two or three individuals, most commonly adults buried together with children, where the distinction and attribution of individual tooth root fragments could still be determined with certainty (Supplementary section SI4, Fig. S2 and Table S2 online).

During ground section preparation, the teeth were subjected to dehydration in a graded ethanol series and embedded in methyl methacrylate (MMA) resin, according to the technique described by Plenk for undecalcified hard tissues^76^. For polymerisation, small glass vials were used as embedding moulds, allowing precise positioning and minimal resin volume. After polymerisation, the resin blocks were carefully removed from the vials and the exposed surface was lightly ground to obtain a flat reference plane. Each embedded root was then transversely sectioned into two halves using a Struers Accutom-50 precision cutting machine, and the two resulting halves were labelled as ‘0’ and ‘1’. Both halves were subsequently surface-ground using 1200-grit abrasive paper and then polished using 4000-grit abrasive material to obtain an optically flat surface. Next, each polished half was mounted on an adhesive-coated microscope glass slide using the cyanoacrylate adhesive Scotch-Weld™ Instant Adhesive CA8 (3M, USA), forming a sandwich-type configuration (‘slide 0’ and ‘slide 1’). The same sandwich-mounting procedure was subsequently repeated to obtain the remaining transverse sections for each specimen.

During the next stage, the slides were manually ground using 1200-grit wet abrasive paper on a circular polisher until sufficient transparency was achieved. After each grinding cycle, the transparency of the section was checked. Before the final polishing step, the surface was additionally refined using 4000-grit abrasive (Supplementary Fig. S8 online), and final polishing was performed using aluminium oxide (Al₂O₃) to ensure optimal microscopic visibility. The thickness of each section was controlled using a micrometric measurement procedure based on focus shift between the top and bottom surfaces of the section (z-plane method). Prior to measurement, the thickness reading of the glass slide was taken in close proximity to the specimen and zeroed, ensuring that only the thickness of the section was recorded. The analysed sections averaged 36.2 ± 11.4 μm, ranging from 22.5 to 65.0 μm (Table 1, Supplementary section SI4 and Tables S1–S2 online).

The slides were documented under transmitted light on an Olympus BX61VS microscope with an Olympus XC10 digital camera mounted. The ILS were also observed under a Nikon ECLIPSE E200 microscope at magnifications of 40, 100 and 400 times. The ILS were counted in Adobe Photoshop graphics software based on documented slide sections where the ILS were most visible and recognizable. The AEFCt measurements were taken in real-time microscopic imaging using NIS-Elements software assigned to a Nikon ECLIPSE E200.

**Fig. 5:**
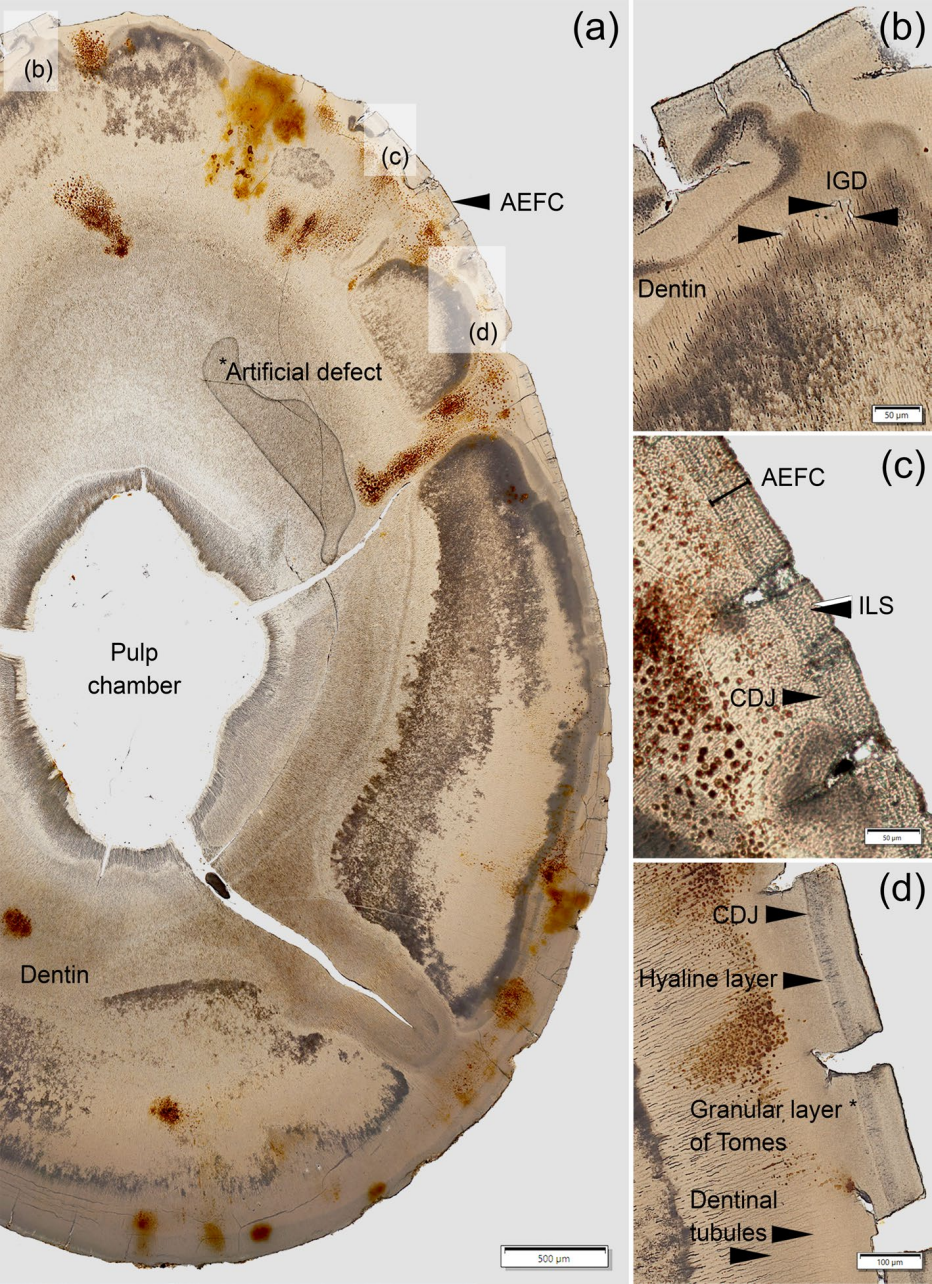
Microscopic analysis of ground sections of undecalcified dental roots from cremated human remains. Observations of dental hard tissues in undecalcified sections (a–d), highlighting the microstructural characteristics of acellular extrinsic fiber cementum (AEFC), the identification of incremental lines of Salter (ILS), and structural features such as the cemento-dentinal junction (CDJ) and interglobular dentine (IGD).

AEFCt was measured on oral (lingual/palatal) and vestibular (buccal/labial)^29^ surfaces where the CDJ border was clearly recognisable, and ILSc values were also obtained. The measurements were taken line-to-line, i.e. between the cementodentinal junction (CDJ) and the last visible AEFC band (Fig. 5, Supplementary section SI4 online).

Statistical procedures were applied to evaluate measurement repeatability, observer agreement, and variation in AEFCt, ILSc, and ILSw in relation to dental, biological, and contextual variables. The reliability of ILSc scoring was assessed by quantifying both intra- and inter-observer error. Intra-observer precision was evaluated by comparing the first and second scoring sessions conducted by Observer 1, performed six months apart to account for potential temporal variation in scoring performance. Inter-observer agreement was examined by comparing the mean ILSc values recorded by Observer 1 with those independently produced by Observer 2, an archaeologist trained in the TCAc method. Reliability metrics included the technical error of measurement (TEM), relative TEM (rTEM%), and intraclass correlation coefficients (ICC), calculated using two-way mixed-effects models with an absolute agreement^77,78^.

Data distribution and homogeneity of variance were assessed using the Shapiro-Wilk and Levene tests. According to these diagnostics, group comparisons were performed using one-way ANOVA, Welch ANOVA, or the Kruskal-Wallis test, with appropriate post-hoc procedures applied when required. Effect sizes were reported as η² or Cohen’s *d* to facilitate interpretation beyond statistical significance.

Repeated measurements originated from the same tooth specimen, linear mixed-effects models (LMMs) were used to account for the non-independence of observations. Separate models were fitted for AEFCt, ILSc, and ILSw, with root region, morphological age category, sex, cemetery, and geographic region included as fixed effects. Tooth section identity was specified as a random intercept to reflect repeated measurements taken from the same specimen. Model significance was evaluated using likelihood-ratio tests, and results were reported as β coefficients with standard errors, 95% confidence intervals, and *p*-values. Pearson’s correlation coefficients (*r*) were calculated to examine the relationship between AEFCt and ILSc, and linear regression models were applied to investigate associations between AEFCt, ILSc, ILSw, and TCA-derived age estimates, with model fit expressed using the coefficient of determination (*r²*). All analyses and data visualisations were carried out using R (packages *lme4*, *lmerTest*, *emmeans*)^79^ and PAST 4.11^80^.

In microscopic studies, determining the AEFCt rendered no problems, so the AEFCt analyses complemented the age at death estimation studies. For this purpose, it was necessary to estimate the width of a single ILS, which would be the divisor for calculating the estimated number of ILS at a known AEFCt. The ILSw was initially measured directly on the slide according to the technique used by Oliveira-Santos and co-authors^26^. However, these measurements varied significantly within the same ILS, so the technique was modified for this study. The single ILSw was calculated as the quotient of the AEFCt and the ILSc (AEFCt/ILSc) based on slides with clearly visible and countable ILS. This approach also made it possible to extrapolate an estimated ILSc for individuals in whom the AEFCt was preserved but the increments themselves were not sufficiently distinct to allow reliable counting. This provided data on (a) the width of the single ILS for individual slides, which were averaged to obtain data on (b) the width of the ILS for the teeth analysed and (c) the width of the ILS for the cemeteries (which are treated here as different LUC populations), as well as (d) the overall width of a single ILS for all individuals studied.

Due to the specific nature of the cremated teeth and many “unknowns” that emerged during the study, mainly related to the effect of high temperature on the potential shrinking effect of AEFC, but also a possibly varied shrinking effect of transparent and opaque bands, the AEFCt, the ILSw and the ILSc were analysed according to the division of the slides into the three areas of the tooth root they had been sampled from, i.e. the apical, middle and cervical part^81^.

## Supplementary Information

Below is the link to the electronic supplementary material.

Supplementary Material 1

## Data Availability

Data regarding AEFCt measurements, ILSc by Observer 1 and 2, and detailed characteristic of cremated tooth root samples are available on the Zenodo platform under the https://doi.org/10.5281/zenodo.15281125 and are included in the Supporting Information.
