# Supplementary material for: Methodological validation and inter-site analysis in Late Bronze and Early Iron Age cremations using tooth cementum annulation counts
Source: Sci Rep. 2026 May 19;16:22850. doi: 10.1038/s41598-026-51841-z (PMC13389445; doi:10.1038/s41598-026-51841-z)
Supplement: Supplementary file 1 — Supplementary Material 1 [file 41598_2026_51841_MOESM1_ESM.pdf]

Supporting Information for

Methodological validation and inter-site analysis in Late Bronze and Early Iron Age cremations using tooth cementum annulation counts

Agata Hałuszko, Stefan Tangl, Toni Dobsak and Fabian Kanz

Corresponding Author: Agata Hałuszko
email: agata@archeolodzy.org

SI1. Overview of cementochronological/TCAc research

Tooth Cementum annulation counts (TCAc) is a histological method based on counting the incremental line of Salter (ILS) in acellular cementum (more precisely, in acellular extrinsic fibre cementum – AEFC), observed in transverse sections of tooth roots, and then adding their number to the age of eruption of the tooth for which the ILS was counted [1–5]. The process of ILS formation in an annual cycle is used for this, in which an increment of one light (translucent) and one dark (opaque) band is formed. In very general terms, it resembles the increment of annual rings of secondary wood [6–8].

General observations regarding the correlation of increasing cementum thickness with the age of an individual were made as early as the second half of the 19th century [9,10]. The first detailed research to check the correlation of the actual age of individuals (humans and other mammals) with cementum thickness [11] and the number of ILS [12] was undertaken in the 1950s. The TCAc method for determining the age of humans was initiated in the 1980s [13,14], but it was not until the early 21st century that a standardized procedure for determining the age of individuals using the TCAc technique was proposed [5]. Single-rooted teeth (incisors, canines and premolars) are most commonly used for TCAc examinations, primarily due to regularity, better ILS observation and a broader range of occurrence within the tooth root: approximately 60-90% of the root of a single-rooted tooth vs 50-30% of the root of a multi-rooted tooth [15]. However, there are no objections to using multi-rooted teeth as well, especially M1 and M2 molars [1,5,16].

It should be emphasized that not all researchers agree on the high correlation of TCAc results with the calendar age of the subjects [17]. Some point to the lack of experience of researchers in making histological preparations [18], the type of resins used [19], the complicated process of sectioning, grinding and polishing the slides [20], the high intra- and inter-observer errors of the ILS counts and thus the impact on the interpretation of the results [21–23] as well as not taking into consideration taphonomic processes during the research [24].

Interestingly, researchers investigating the process of ILS formation in different mammalian species have begun to highlight important issues related to seasonal variation and hypomineralization of ILS bands under various climatic conditions [25–29]. These differences have also started to be seen in association with the nutrition and biomechanics of the masticatory organ [30], as well as the phases of fertility [31]. The occasional phenomenon of ILS doubling has also begun to be identified [32,33], which may significantly impact and cause an erroneous age estimation using the TCAc method. On the other hand, in humans, an appositional growth of ILS has begun to be interpreted as a manifestation of physiological stress associated with, among other things, pregnancy, skeletal trauma and other diseases [34–38]. It also led to questioning some of these analyses [39].

Despite the potential multidimensional research results obtained using the TCAc method, it is not commonly applied to archaeological human remains [20,22,40–43]. The reasons for this are

probably the tedious and labour-intensive process of preparing microscope slides, the small number of laboratories specializing in the TCAc method and the high cost of analysis, but also the destructive nature of the research [22,44,45]. There is also an important aspect of the reliability of TCAc results obtained for individuals from archaeological contexts whose calendar age is unknown. TCAc studies based on archaeological samples highlight the influence of post-depositional factors that may hinder ILS observations [20,24,37,42,46].

Some authors are also inclined to use age at death estimation methods based on the measurements of AEFC thickness [47,48] and indirectly of a single ILS measurement [37], which appears to be a much simpler technique, especially for archaeological samples with indistinct ILS [20]. The AEFC growth rate, depending on the tooth and its surface, is approximately 0.004-0.008 µm/day [21,40,49], and this cycle is believed to be constant over the entire lifetime [11,50]. The thickness of the AEFC, depending on the age of humans, is assumed to be approximately 50-200 µm [15], and the AEFC is mainly composed of mineralized collagen fibres [51,52].

SI2. Archaeological background

The study was conducted on 62 cremated teeth, from which 292 slides were obtained for further analysis. The teeth came from 60 individuals identified at eight archaeological sites associated with the Lusatian Urnfield Culture (Fig. S1 and S2, Table S1). These cemeteries are generally dated to the Late Bronze Age and Early Iron Age, i.e. c. 1100-450 BC [53–59]. The analyzed osteological materials had been cremated; they were characterized by white, light grey and cream colours, and sometimes they displayed a “sandwich effect” [60].

Archaeological Background of the studied sites

Cieszków

The cemetery has been known since the 19th century, and the first major scientific excavations were undertaken in 1926 by E. Boehlich [61]. At that time, 50 graves were discovered, 10 of which were destroyed and their methodical exploration was impossible. The excavated burials were mostly dated to the Hallstatt period C, while two to the Bronze Age period V [55,62]. Later rescue works were carried out on the orders of the Conservator of Archaeological Monuments for the Wrocław Voivodeship in 1955 and verification works in 1964. From both of these research seasons, another 4 cremation graves were obtained - two graves from each season [62]. In 1973, systematic excavations were undertaken, led by Dr. J. Domańska and J. Gołubkow from the Archaeological Museum in Wrocław. The works lasted for 4 seasons and a total of 120 cremation graves were explored [62–67]. Among the discovered grave complexes, the presence of 4 symbolic graves and 1 animal grave was identified.

Anthropological analyses were performed after each excavation season by Dr. B. Miszkiewicz, Dr. M. Magnuszewicz and (at that time) M.S. K. Borysławski. The results of these studies were not published as a separate anthropological publication, but their results were included in archaeological publications. The presence of 143 individuals was identified in 120 graves, of which 44.76% were children, and approx. 11% of individuals lived to the age of Senilis [64–66,68].

Most of the osteological materials were not available for repeated anthropological analyses during the cementochronological studies. TCAc studies were conducted on one sample of a cremated tooth root (FDI X4/X5) from an individual of unknown sex and age at death from double grave no. 14 (Table S1 and S2).

Czernikowice

The cemetery in Czernikowice has been known since 1916 [61]. Most likely 97 cremation graves were discovered at that time. Their exact number is an estimate, as both detailed documentation and archaeological artifacts were dispersed after 1945 [53]. Excavations were resumed in 1968 in connection with the commencement of aggregate exploitation in the cemetery. During the first excavation season, research covered 14 ares and 65 objects dated to the transitional period between the 4th and 5th period of the Bronze Age were explored. In the following year, research covered 6 ares and 18 graves were discovered [69]. The third season of research was continued in 1971 and excavations covered 30 ares of the site, where 87 burials were documented. The next and last excavation season took place in 1972 and covered an area of 9 ares, where 35 objects were identified [70].

Anthropological analyses were performed on burnt bone remains obtained in the 1960s and 1970s. Of the approximately 205 graves, only bones from a total of 24 features could be subjected to anthropological reanalysis (Fig. S3): from 20 graves and four bone clusters, because most of the osteological materials were lost. For 100 graves, archival anthropological documentation prepared by Dr. Brunon Miszkiewicz has been preserved, which was included in the analytical and interpretative studies of the entire necropolis [53]. All preserved collections are under the care of the Museum of Copper in Legnica [53].

The MNI (minimum number of individuals) was determined for 146 individuals, of which 140 (95.2%) had their age at death determined. The largest group consisted of children (69.2%: Infans I – 52.7%, Infans II – 16.4%), followed by Adultus – 13.0%, and the smallest were individuals from the oldest age categories – 1.5%. Determining the sex of most individuals (72.1%) was impossible [71]. This was due to the significant percentage of individuals classified in the youngest age categories. Of the 146 individuals, 22 females and 17 males were identified using standard anthropological methods [53].

TCAc studies were performed on five teeth, including two roots of deciduous molars, from three individuals (Tables S1 and S2). Due to the dissolution of two teeth from children (Fig. S6 and S7), it was necessary to repeat the analyses. In one individual, the presence of a partial duplication of the ILS number was identified.

Krzyżowice

The site was discovered in 1961, when rescue excavations were undertaken at the request of the owner of the field, who had found fragments of clay vessels during agricultural work [72]. After familiarizing oneself with the terrain situation and recognizing the collected archaeological artifacts, excavations were undertaken in the places where the most numerous ceramic clusters occurred. Two excavations were established with a total area of 1.5 ares and 10 urn graves of the so-called Lusatian culture were explored. Due to the shallow depth of deposition (0.26-0.60 m), the burials were partially destroyed. A painted bowl was discovered in one of the graves, while the ceramics recovered from the other burials were devoid of decorative patterns. Based on artifactual dating, the chronology of the cemetery was determined to be the Bronze Age IV and V period and the early Iron Age (HC).

Archaeological research took place in two stages in 2013 and 2014 and was conducted by the archaeological company DELFA Robert Szwed. In total, the research covered an area of approx. 15 ares. 28 cremation graves were explored, of which osteological materials from 22 vessels from 18 graves were transferred for anthropological research, although four well-preserved urns have not been explored because they are part of non-invasive research. Chronologically, among the discovered burials, 11 graves (56.6%) were distinguished dated to the Early Iron Age, including 10 graves dated to HaC and one to HaD. For the remaining burials, the chronology was broadly defined from III BAP to the Early Iron Age (Ha).

The minimum number of individuals (MNI) from 14 graves was estimated at 16 individuals. Age at death was determined for 14 individuals, and biological sex for 11: seven females, four males, and five undetermined. Among the examined bones, mainly graves of single individuals were identified (80.0%), while in 2 burials (grave 11, vessel 1 and grave 28, vessel 12) the remains of two individuals were placed in one urn. In both cases, these were graves of a juvenile or a child with an adult.

TCAc studies were performed on the roots of permanent teeth from six individuals (Tables S1 and S2).

Łazy

The necropolis has been known since the 19th century, and the most recognizable artifact from one of the graves is a clay urn with a narrative engraving depicting a scene of "hunting deer" [73]. Since the beginning of the 20th century, there have been mentions of further finds of various

archaeological artifacts from the dune area [61]. In the 1990s, the cemetery again became the object of deliberate plundering aimed at obtaining valuable archaeological artifacts. As a result of these activities, several grave complexes were destroyed. At the request of the State Service for the Protection of Monuments in Wrocław, rescue and security excavations were undertaken at that time, which were continued in the 1997-1998 seasons, and were managed by Dr. Paweł Madera [58,74]. Osteological materials obtained during these excavation seasons were used in these studies.

In general, osteological materials from 6 cremation graves dated to the Bronze Age V to the Early Iron Age (Ha C) were examined. The MNI was estimated at 16 individuals. Assignment to specific age at death categories was possible for 15 individuals; one individual was assigned to the general age category of adult. Biological sex was estimated for 8 individuals (three females and five males), for eight individuals sex could not be estimated. In individual graves, the presence of 2 to 6 individuals was identified, with 71.4% of cases the remains of a single individual being placed in one urn, 14.3% the remains of two individuals being mixed together in one urn, and 14.3% the mixed remains of two individuals occurring in two urns.

TCAc studies were performed on five adult individuals (Tables S1 and S2).

Rolantowice

The cemetery in Rolantowice has a multicultural character and has been known since the beginning of the 20th century [56,75–78]. Among other things, 8 cremation graves of Lusatian urn fields dated to the Old Bronze Age and Early Iron Age were discovered in the necropolis, as well as one skeletal burial of difficult to determine chronology [77,78]. In addition, pottery was obtained from destroyed graves of the Funnel Beaker culture [77] and materials from the younger phase of the Globular Amphora culture [75] and 2 graves of the Corded Ware culture [79] were examined, the chronology of which was established based on radiocarbon dating.

Anthropological studies were conducted on osteological materials from excavations conducted in 2013 on the site of today's gravel pit (Trans-Piach Sand Mine in Rolantowice). The research was conducted by the archaeological company DWOJAK – Andrzej Dwojak under the direction of Wojciech Łęcki. During these excavations, 9 cremation graves from Lusatian urn fields were discovered. The graves contained 2 to 22 vessels, as well as bronze objects. The chronology of the objects was established based on relative dating to the Hallstatt period C (Ha C). The vessels from 3 graves were preserved in their entirety together with the filling (an urn and one of the adjoining vessels), therefore, after the excavations were completed, they were subjected to tomographic examinations [56].

In general, anthropological analyses were performed on bones from 7 cremation graves. Materials from 2 graves subjected to CT scanning at a later time were processed based on virtual bone imaging and constitute a valuable source of information on bioturbation processes. In 7 analyzed burials, the presence of 9 individuals (MNI=9) was found, with sex estimated for two

female individuals, and for the remaining biological sex not assessed. Age at death was determined for only one Infans I, two Adultus and three adult individuals, for the remaining age at death was not estimated. Each individual was placed in a separate urn, and the presence of 2 individuals was identified in two graves.

TCAc studies were performed on three individuals (Tables S1 and S2). In one individual, a partial duplication of the ILS number was identified.

Świbie

The biritual cemetery in Świbie was accidentally discovered during forest planting in 1936, and in the same year, archaeologist Franz Pfützenreiter from the Oberschlesisches Landesmuseum in Bytom conducted rescue research there, during which he uncovered two graves. Their results were published two years later by Friedrich Hufnagel. After the war, from 1961 to 1967, Anna Stankiewicz-Węgrzykowa, archaeologist and head of the department of the Museum in Gliwice, conducted systematic archaeological research. In the following years, 1970-1992, the excavations were continued by Halina Wojciechowska, then head of the archaeology department at the Museum in Gliwice. During these studies, a necropolis of the Lusatian culture was discovered, covering an area of approx. 1 ha, with 576 graves identified, including 490 skeletal graves and 86 cremation graves [59,80].

Osteological materials were preserved in only about 33% of the graves. Anthropological analyses were performed on bones identified in 220 graves: 100 skeletal, 93 cremated and 27 biritual [81]. In total, 242 individuals were found: 114 inhumed and 128 cremated. In 21 burials, more than one individual was identified, and in three of them, the remains of 3 individuals were found. Gender estimation was possible in 62 cases (25.6%), although due to the very poor state of preservation of the skeleton of inhumed individuals, it was more common to determine the sex of individuals from cremated graves (26.6%) than from skeletal graves (24.6%). Tomographic examinations were also undertaken for 33 individuals to determine sex based on the LA method [71,81]. Age at death of individuals assigned to a category was determined for 98 individuals (40.5%): 32 (28.1%) from inhumation graves and 66 (51.6%) from cremation graves. Additionally, 83 individuals (34.3%) were classified into general age categories, i.e. "child" – 8 individuals (3.3%): four each from cremation and skeletal graves, and "adult" – 75 individuals (30.9%): 50 (43.9%) from inhumation graves and 25 (19.5%) from cremation graves.

For 27 cremated individuals from 26 graves, precise age determination was obtained based on the TCAc method (Fig. S4, Tables S1 and S2).

Wrocław-Żerniki

This site was accidentally discovered in 1925 and was defined as a cremation cemetery of the Lusatian culture from the 3rd-5th period of the Bronze Age. Research conducted in 1996 and 2005

did not confirm its existence in this place. During the rescue research and later excavations in 2007 and 2009 conducted by a research team from the Institute of Archaeology of the University of Wrocław under the supervision of Dr. Mirosław Furmanek, the remains of a cremation cemetery dated to the Late Bronze Age and Early Iron Age were discovered [54].

During excavations in 2009 related to the construction of the western part of the Wrocław bypass in an area of approx. 30 ares, 50 objects were discovered. The graves were located shallowly in the sandy substrate and were exposed to mechanical destruction related to the presence of allotment gardens in the area. The burials had no stone structures and the grave pits were poorly legible. The ceiling sections of the burials were disturbed and burnt bones from damaged urns were located in the cultural layer. Some of the complexes did not contain burnt bones. The chronology of the burials was determined based on the artefactual equipment to be period IV-V of the Bronze Age and the early Iron Age (HC) [54].

In total, the presence of osteological materials was identified in 43 graves. The MNI was estimated at 47 individuals. Most of the urns contained the remains of single individuals, only in the urn from grave 21 were the mixed remains of 2 individuals identified. In the burials, however, the presence of 1 to 3 individuals was found. Based on the methods of sex assessment based on morphological features of the skeleton, precise determination of age at death was possible for 22 individuals. For 14 individuals, a general age classification was applied, i.e. "child" and "adult". In the remaining cases, determination of age at death was not possible.

From the examined materials from 4 graves, fragments of permanent teeth roots were selected for histological TCAc analyses (Tables S1 and S2). Gender was determined using morphological methods only for 4 individuals.

Wtórek

The cemetery in Wtórek was accidentally discovered in 1999 by the then owner of a farm located near the cemetery [82]. Surface archaeological verification preceding the excavations confirmed the existence of a Lusatian culture necropolis in this place. Before the excavations began, there was an agricultural field in the cemetery.

The large-scale archaeological excavations were of a commercial nature and were conducted in the years 2012-2016 by a consortium of two companies: Usługi Archeologiczne Grzegorz Gmyrek and MBL Leszek Ziąbka. The works were commissioned by GDDKiA and were conducted in connection with the construction of the Ostrów Wielkopolski bypass and the reconstruction of the district road from Ostrów Wielkopolski to Grabów nad Prosną.

As part of the excavations carried out on over 220 ares, the presence of a settlement and a cemetery of the Lusatian Urnfield culture was recorded, as well as traces of medieval and modern settlement. In the necropolis of interest to us, covering an area of approx. 27 ares, 222 cremation graves with varying degrees of preservation were discovered. Most of the burials were destroyed

due to the small depth of deposition and the related damage caused by agricultural activity. In the examined part of the cemetery, only the occurrence of so-called flat graves was noted, which are currently not distinguishable on the surface. In a few cases, the outlines of burial pits were preserved (16.7%). Most of the discovered burials were urn graves (82.9%), in which human bones were placed in a vessel-urn.

During the anthropological analyses, the presence of 257 individuals was found in 222 discovered graves [83]. Biological sex estimation was possible for 32.2% of individuals: 23.3% females and 8.9% males. Age at death could be determined for 61.5% of individuals. The largest group in the age structure were children (30.7%). Fragments of cremated teeth from 12 individuals were selected for TCAc studies (Fig. S5, Tables S1 and S2).

SI3. Age at death and biological sex estimation

Age at death estimation

Prior to the TCAc analyses, age at death was estimated using standard morphological methods commonly applied in bioarchaeological research. In adults, age assessment was based on the combined evaluation of multiple osteological indicators, including the degree of cranial suture obliteration (endocranial and ectocranial), epiphyseal fusion, dental development, and, in several cases, morphological changes of the auricular surface of the ilium [84–92]. The degree of cranial suture obliteration was recorded separately for individual segments of the coronal (C1–C2), sagittal (S1–S4) and lambdoid (L1–L3) sutures, distinguishing endocranial and ectocranial expression [84,93]. Cranial suture obliteration was treated as one of several age-related indicators and interpreted cautiously in conjunction with other skeletal traits, rather than as a primary determinant of adult age**.**

Dental indicators included the stage of permanent root formation (with particular reference to apex closure, Ac stage), crown formation stages in subadults (e.g., Cr½, Cr¾, Crc), and the presence of fully formed and open alveoli in the maxilla and mandible (recorded according to FDI notation where preservation permitted) [92,94].

In selected individuals, additional skeletal markers were considered, such as fusion of the spheno-occipital synchondrosis, fusion of the sternal epiphysis of the clavicle, fusion of the iliac crest epiphysis, and fusion of pelvic elements at the acetabulum [95].

These criteria were interpreted jointly, with particular attention to the preservation state and diagnostic value of individual skeletal elements. No single indicator was used in isolation to determine the final osteological age estimate; instead, concordance among available traits was evaluated in each case (see Table S3).

For subadult individuals, age estimation relied primarily on the stage of skeletal development, including epiphyseal fusion of the long bones, as well as the degree of dental formation and eruption [92,94–96]. Particular emphasis was placed on the stage of crown and root

development of deciduous and permanent teeth, the presence of developing tooth germs, and the state of fusion of primary ossification centres and epiphyses of long bones, including the proximal and distal epiphyses of the humerus, femur and tibia, as well as other preserved elements.

Age categories were used according to the European standard [97,98], allowing for consistent classification of individuals across the analysed assemblage, following standard bioanthropological practice (see Table S3). Individual age ranges were derived through concordance of independent osteological indicators rather than from averaged values or a single anatomical trait. Assignment to broader age categories (Infans I, Infans II, Juvenis, Adultus, Maturus, Senilis or transitional categories such as Adultus/Maturus), was therefore intended to minimise interpretative error associated with fragmentary preservation and the variable expression of individual age markers.

The study was carried out on nine subadults: two Infans I, four Infans II, one Infans II/Juvenis, two Juvenis, as well as 49 adults: 29 Adultus, 10 Adultus/Maturus, three Maturus, two Maturus/Senilis, and five individuals classified broadly as adult due to limited diagnostic indicators. It was impossible to estimate the age at death for two individuals due to the lack of diagnostic bone fragments (Table S3).

For each individual, the preserved diagnostic elements, the specific age indicators used, their observed stages, and the methodological references are presented in detail in Supplementary Table S3. The column “Confidence of age assessment” reflects the robustness of the osteological inference based on the number, preservation and diagnostic value of the available traits, and does not represent a statistical posterior probability.

In the case of two individuals from the cemetery in Czernikowice (from graves 22 and 74), two teeth of each were examined, due to the problems encountered during the preparation of the first selected samples (cf. Table S2). We concluded that the issues noted during these analyses could be of interest in the context of similar studies in the future; therefore, both samples for each individual were included in the remainder of this study.

Sex estimation

For individuals in whom diagnostic skeletal traits were preserved, sex estimation was performed using morphological methods. Sex was assessed on the basis of preserved diagnostic traits, primarily using non-metric criteria observable on the cranial bones, with particular attention to the frontal, occipital and zygomatic bones and the mandible, as well as on the postcranial skeleton [87,99,100]. For each individual, we recorded (i) preserved diagnostic elements, (ii) the sexually dimorphic traits assessed, and (iii) the trait assessment (i.e., the observed expression, including ordinal scores where applicable), together with the methodological references used (Table S4).

Sex classification into female or male categories followed the morphological approach proposed by Strzałko, Piontek and Malinowski [101,102], based on the combined evaluation of individual sexually dimorphic traits [99].

Cranial traits included, where preserved, the morphology of the glabella, supraorbital margins and supraorbital region (including the development of the supraorbital ridge), the external occipital protuberance and nuchal lines/crest, and the size of the mastoid processes. Mandibular traits comprised the morphology of the mental eminence and the size/robusticity of the mandibular condylar process. Zygomatic traits included the size and robusticity of the zygomatic bone and the morphology of the frontal process of the zygomatic bone, including the presence/absence and expression of the marginal tubercle.

Pelvic traits (when preserved) included the morphology of the greater sciatic notch, the auricular surface and the presence and expression of the preauricular sulcus [99,100,103]. Postcranial traits were used as supportive indicators and included, depending on preservation, the size of the humeral trochlea, the diameter/robusticity of the humeral head, and the diameter/robusticity of the femoral head. In selected cases, the axis (C2) was also assessed, including the metric/morphological expression of the odontoid process [101,102,104].

Trait expression was recorded using standard descriptive terminology and, where applicable, ordinal scoring (e.g., 1–5 or 1–2/3–4 ranges reflecting trait expression), allowing traits to be reported consistently as female, male, or intermediate morphology, particularly for cranial indicators [99]. Osteometric methods were also applied, based on measurements of the zygomatic bones, dimensions of the mandibular condylar process, metric parameters of the axis (C2), including the odontoid process, the diameter of the humeral head, and the diameter of the femoral head [101,102,104,105] (Table S4). In individuals lacking sexually dimorphic elements or preserving only non-diagnostic fragments, sex was recorded as “not determined”. In subadult individuals, sex estimation was treated with particular caution due to the limited expression of sexual dimorphism prior to adulthood, and where appropriate no final sex assessment was provided despite recording individual traits (Table S4). CT-based assessment of the petrous part of the temporal bone using the lateral angle method (LAM) [71,81,83,106–109] was not used for sex estimation in this study and was therefore not included as a variable in Table S4 or in the main analyses.

The final sex assessment was expressed together with a confidence value (%) reflecting the strength of classification based on the number, preservation and diagnostic value of the evaluated traits, in accordance with the applied osteological methodology [101,102]. This value represents an interpretative assessment of classification strength and does not constitute a statistical or probabilistic estimate (Table S4).

When trait expression was conflicting or based on a limited number of features, sex was reported conservatively as “possibly female” or “possibly male” (F?/M?) rather than forcing a binary assignment (Table S4) [100].

Within the analysed assemblage, 30 individuals were assessed as female, 20 as male, and 10 remained of undetermined sex. In further analyses, given the potential association between abnormalities observed in the AEFC, the sex of individuals was treated with caution due to the limitations associated with sex determination and the diagnostic value of cremated skeletal material (Tables S2 and S4).

SI4. Selection and preparation of cremated tooth roots with accompanying documentation of ground transverse cross-sections

There was an attempt to select single-rooted teeth for histological examinations, mainly premolars, but due to the nature of the cremated prehistoric material, we were often forced to use preserved tooth fragments, including molar roots. Most of the teeth came from the burials of single individuals. Teeth from 25 individuals were selected from the burials of two or three individuals, most of which were burials of adults with children, making it possible to assign specific tooth root fragments to particular individuals. In the case of two individuals from the Adultus category, from grave 175 from Świbie, teeth were extracted from the alveoli, while in the case of grave 427 from the same cemetery, it was impossible to assign a tooth to a particular adult individual.

The teeth examined were preserved as tooth roots. Teeth that were not embedded in the alveoli were identified based on their morphology [110–112]. Teeth were numbered using the FDI system [113]. Tooth fragments not numbered with an explicit FDI notation were marked with the letter X and a number indicating the order of the tooth in the dental arch (e.g. X3 – canine). The age of individuals was estimated by counting ILS and adding to the sex-averaged age of eruption of the tooth studied [114]. Teeth marked with an X were assigned an eruption age according to the differences in eruption time between upper and lower teeth.

The teeth analyzed included eight incisors, 16 canines, 23 premolars, 12 molars, and three deciduous molars (Fig. S2; Table S2). Single-rooted teeth, mainly premolars, were preferentially selected for histological examination. In several cases, however, the condition of the cremated material necessitated the use of preserved root fragments, including molar roots, because no other sufficiently large and morphologically recognisable tooth roots suitable for TCAc analysis were available. The tooth roots labelled as X6/X7 were extracted from alveoli in damaged maxillary and mandibular fragments located adjacent to other open alveoli, which prevented confident identification as either X6 or X7. In contrast, no such ambiguity occurred in the case of the tooth roots labelled as X8. Owing to the destructive nature of sampling and the museum-curated status of the assemblage, it was not possible to analyse multiple fragments from the same individual.

During ground sections preparation, the teeth were subject to a dehydration procedure and embedded in methyl methacrylate (MMA) based resin, according to the technique described by Plenk for undecalcified hard tissues [115]. Histological preparations and analyses were carried out at the laboratory of the Center for Forensic Medicine, Medical University of Vienna, while scanning of

the slides was performed at the Core Facility Hard Tissue and Biomaterial Research, Karl Donath Laboratory, Medical University of Vienna. Teeth embedded in resin were subject to transverse cutting into between 2 to 10 layers, averaging to about 5 layers, depending on the length of the tooth root fragment to be analyzed. During the next stage, due to the highly variable transparency, the slides were manually ground to a thickness that allowed microscopic observation. The transparency of the slide was checked each time after a complete grinding cycle under transmitted light with a Nikon ECLIPSE E200 microscope. In cases where optimum transparency was not achieved, the entire grinding cycle was repeated until the required level of transparency was obtained; otherwise, microscopic analysis of dental tissues would not have been possible. After grinding, the slides were polished with aluminium powder until a scratch-free surface was produced across the transverse section of the tooth. Because the transparency of cremated material was highly variable, the thickness of the slides could not be standardised and depended entirely on the degree of transparency achieved during preparation; consequently, thickness ranged from 22.5 to 65.0 µm, with a mean of 36.1 µm (Fig. S8; Table S1 and S2).

The slides were documented under transmitted light on an Olympus BX61VS microscope with an Olympus XC10 digital camera mounted. The ILS were also observed under a Nikon ECLIPSE E200 microscope at magnifications of 40, 100 and 400 times. The ILS were counted in Adobe Photoshop graphics software based on documented slide sections where the ILS were most visible and recognizable. AEFCt measurements were performed using real-time microscopic imaging in NIS-Elements software connected to a Nikon ECLIPSE E200 microscope. The measurements were taken line-to-line, i.e. between the cementodentinal junction (CDJ) and the last visible AEFC band. Based on previous studies reporting no statistically significant differences in AEFC thickness between oral (lingual/palatal) and vestibular (buccal/labial) surfaces [116], AEFCt was measured on these surfaces wherever the CDJ border was clearly recognisable, and ILSc values were obtained concurrently.

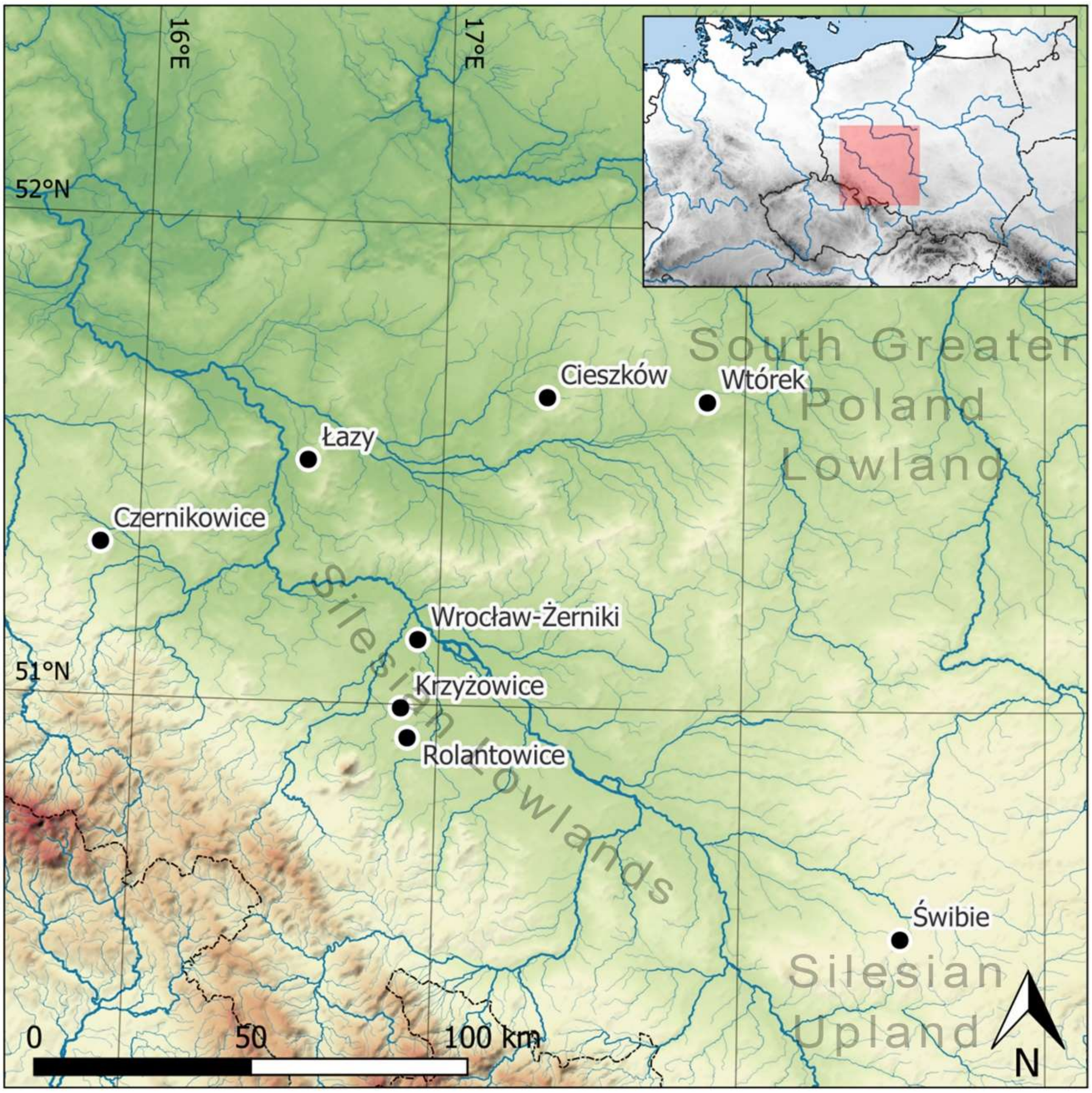

Fig. S1. Location of archaeological sites. The map was created in QGIS 3.34 (https://qgis.org) using NASA Shuttle Radar Topography Mission (SRTM) open data distributed by OpenTopography (https://opentopography.org; https://doi.org/10.5069/G9445JDF; accessed 1.05.2025) and vector layers from the EU-Hydro River Network Database, Version 1.3, obtained from European Union's Copernicus Land Monitoring Service (https://land.copernicus.eu/; https://doi.org/10.2909/393359a7-7ebd-4a52-80ac-1a18d5f3db9c; accessed 1.05.2025) (map prepared with the assistance of M. Mackiewicz).

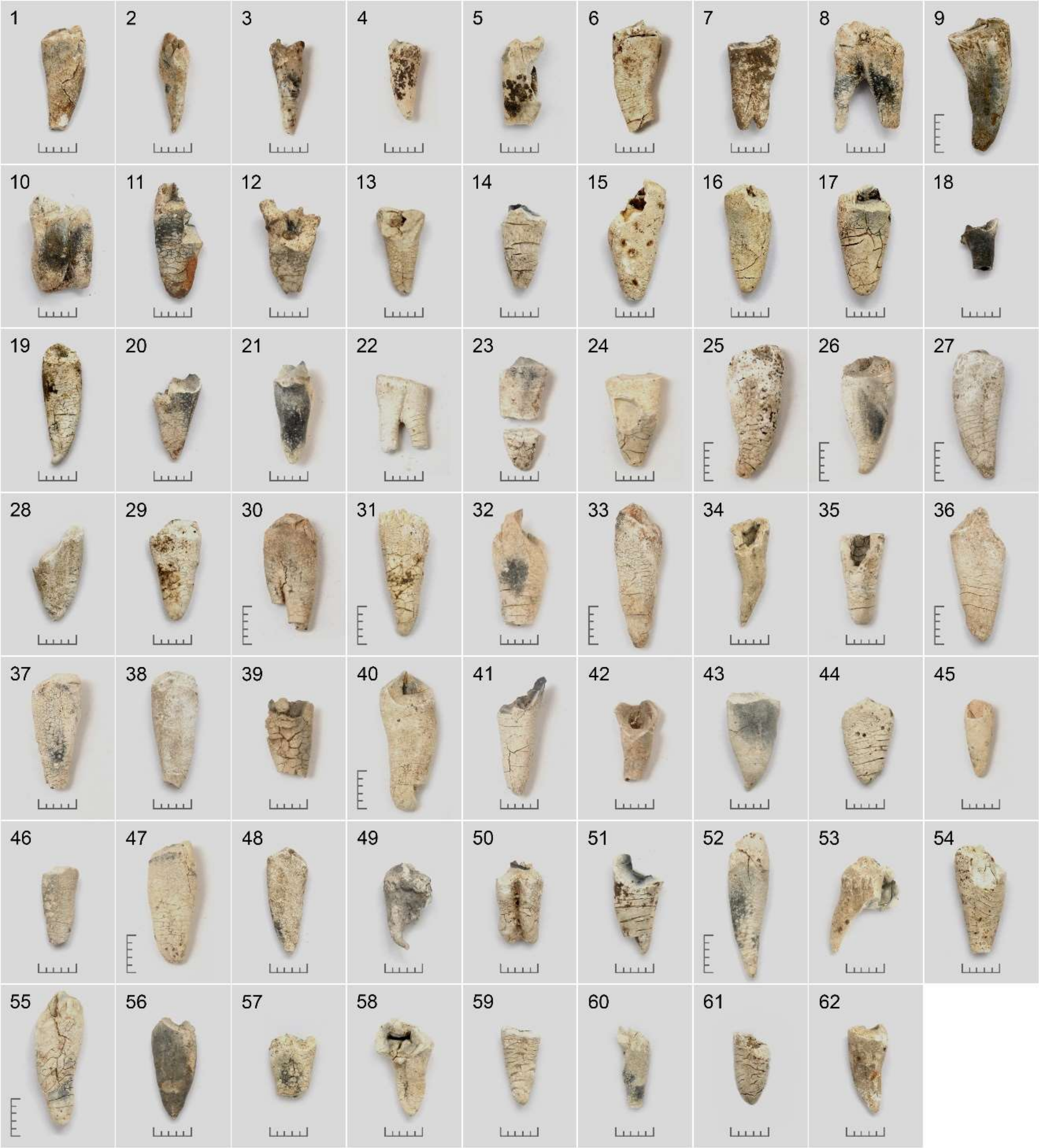

Fig. S2. Fragments of cremated teeth subject to histological analyses; numbers correspond to the sequence number in Table S2; scale bar 0,5 cm (prepared by A. Hałuszko).

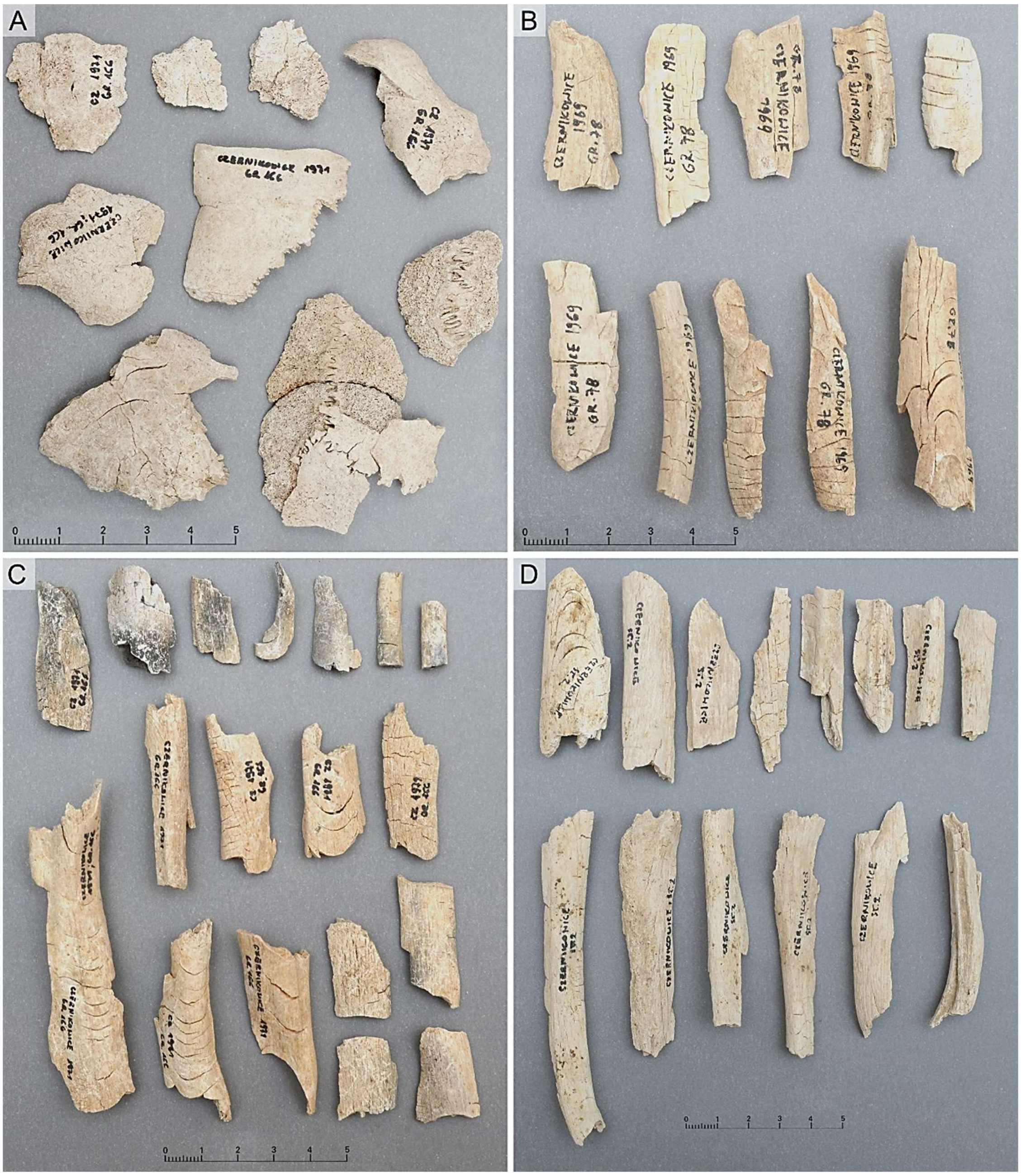

Fig. S3. Czernikowice, sites 2 and 4, Chojnów commune: A - burnt cranial vault fragments with well-preserved cranial sutures of a mature female from burial 166; B - cracking and delamination of cortical layers in burnt diaphyseal fragments of an Adultus possible female from grave 78; C - uneven burning intensity of diaphyseal fragments of a mature female from burial 166; D – even burning intensity of diaphyseal fragments of a probable Adultus/Maturus male from burial Cz/18 (photos A. Hałuszko) [53].

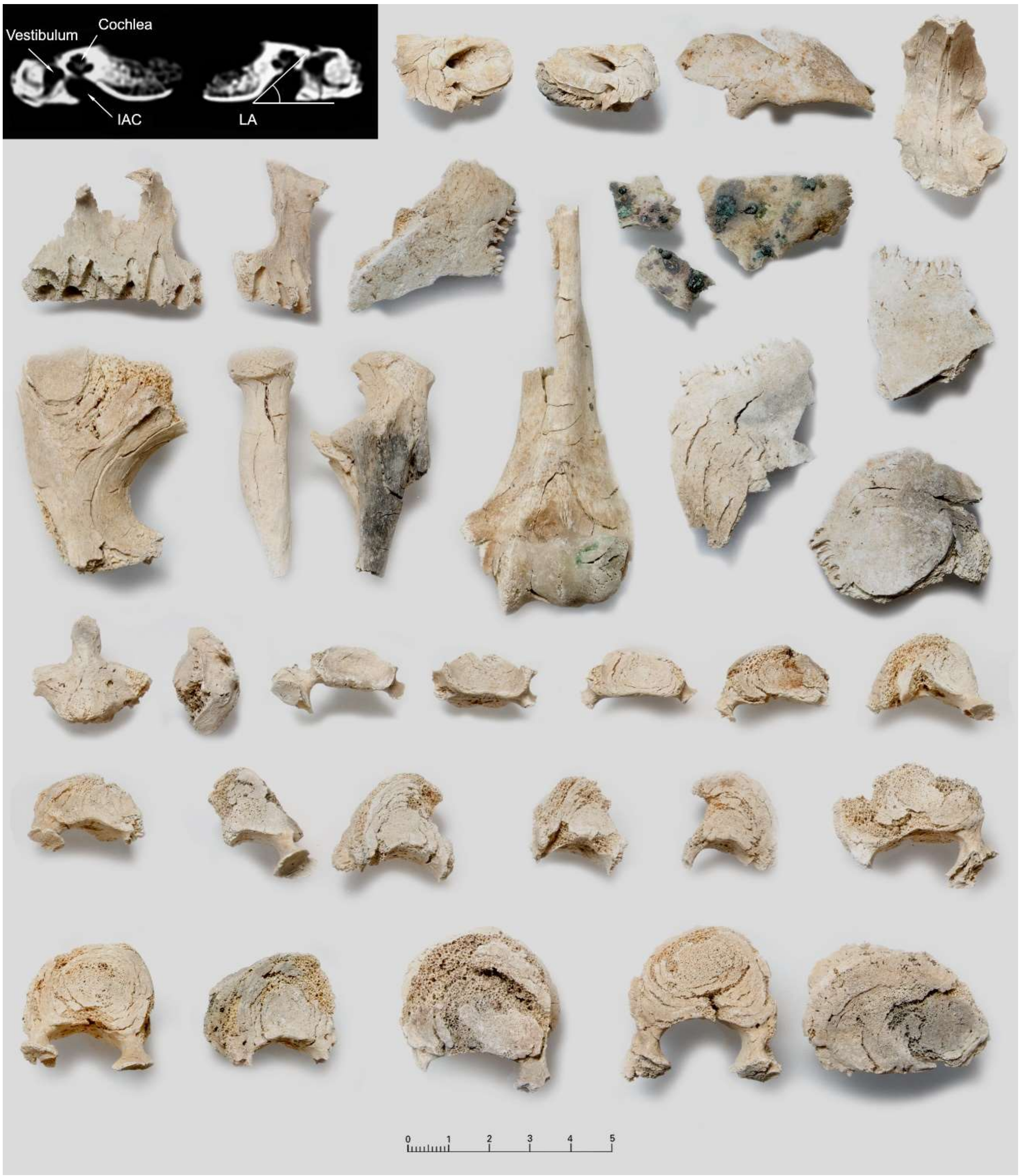

Fig. S4. Cemetery at Świbie, cremation grave no. 31. Selected preserved bone fragments of a possibly female individual of Adultus age with an MPR reconstruction of the petrous parts of the temporal bones: vestibule (*vestibulum*), cochlea, internal auditory canal (IAC), and LA – measurement of the lateral angle (photos A. Hałuszko) [81].

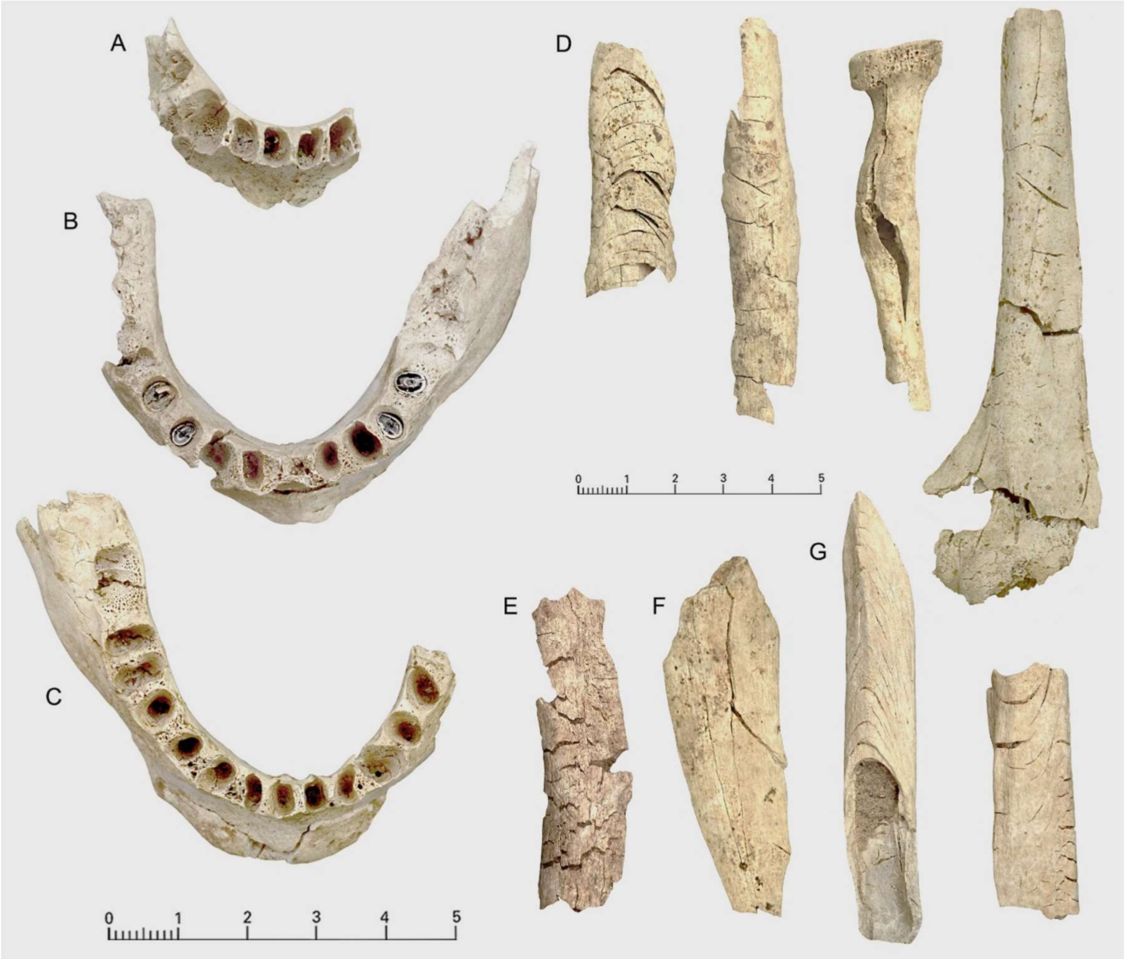

Fig. S5. Wtórek, site 7 (AZP 69-36/51), Ostrów Wielkopolski commune. Fragments of the mandibular body from A – grave 540, B – grave 537 and C – grave 578, and selected diaphyseal fragments of long bones from D – grave 526, E – grave 480, F – grave 723 and G – grave 889 (photo: A. Hałuszko).

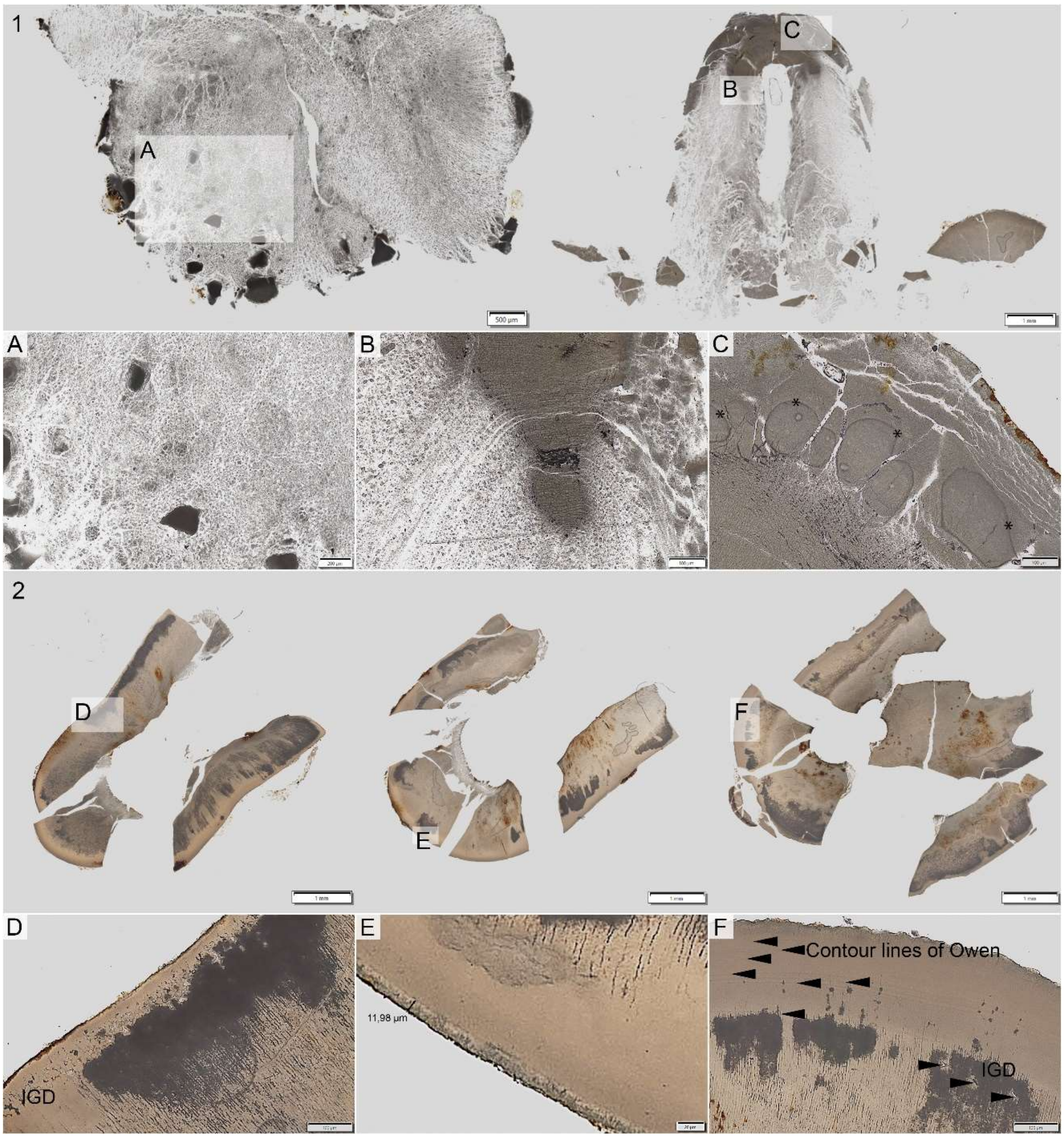

Fig. S6. Imaging of slides from two permanent teeth of a child from grave 74, from the burial ground in Czernikowice. Tooth 12/22, which underwent unexplained destruction during embedding (1): A – dissolved dentin; B, C – fragments of preserved dentin tissue structure. Tooth 31/41, which underwent partial fragmentation during embedding (2): D – defects in dentin mineralization in the form of IGD; E – a fragment of the slide with well-recognized AEFC; F – defects in dentin development and mineralization in the form of contour lines of Owen and IGD; areas with artificial defects (glue edges) occurring in the slide preparation process are marked with asterisks (prepared by A. Hałuszko).

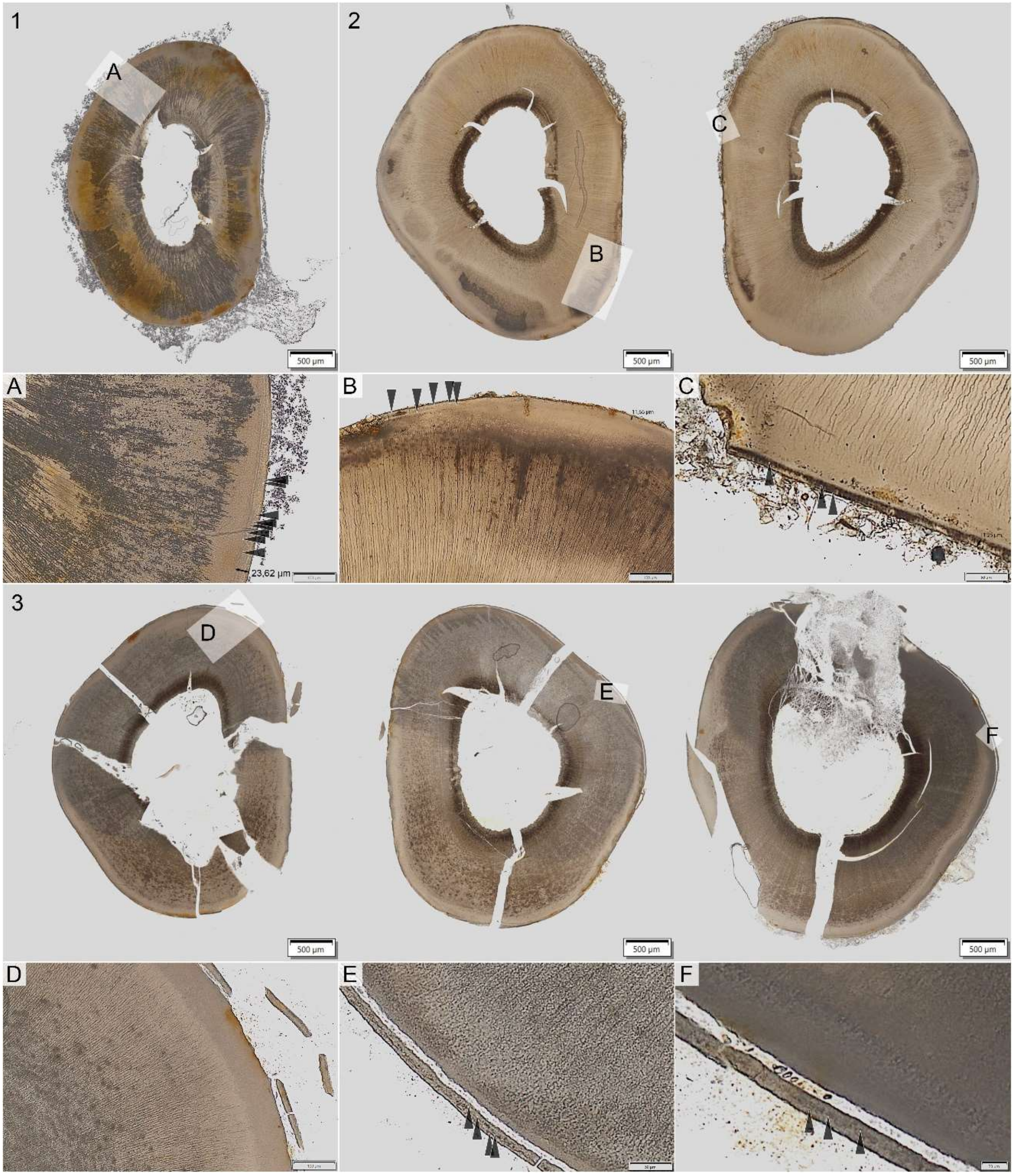

Fig. S7. Imaging of slides of children's deciduous teeth from the cemeteries in Świbie (1) and Czernikowice (2, 3): A – AEFC with recognizable ILS from the tooth of an individual from grave 543; B-F – state of preservation and AEFC with recognizable ILS from the two teeth of an individual from grave 22 (prepared by A. Hałuszko).

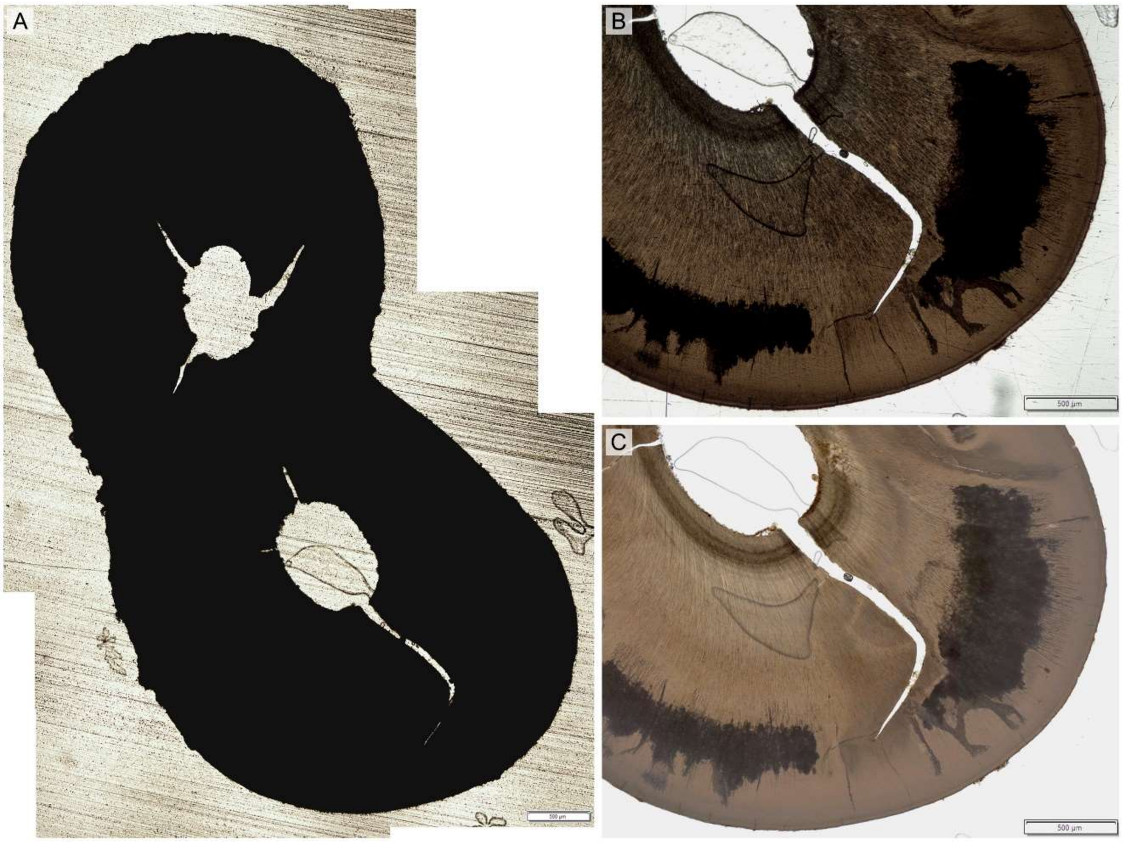

Fig. S8. Variability in the transparency of a transverse section of a cremated maxillary first premolar (FDI 14/24) root from the Krzyżowice site (specimen TCA 76, no. 7 in Fig. S2), examined at different section thicknesses: A – transverse section ground to approximately 100 µm, with no observable transparency (the section was not yet polished at this stage, as polishing was carried out only after adequate transparency had been achieved); B – intermediate preparation stage, showing partial transparency at approximately 70 µm (for imaging purposes only, the section was lightly pre-polished with 4000-grit abrasive paper; final polishing was not performed at this stage); C – transverse section ground to approximately 50 µm, where sufficient transparency was achieved and final polishing completed, resulting in clear visibility of dental tissues (prepared by A. Hałuszko).

Table S1. General characteristics of the materials used in the study.

Site	Voivodeship	Geographical region	PAR (AZP)*	Latitude and longitude	Number of examined individuals	Number of examined teeth	Number of analyzed tooth ground sections	Number of analyzed tooth ground sections with countable ILS	Mean number of sections from one tooth	Mean thickness of the ground sections ± SD
Cieszków	Lower Silesia	South Greater Poland Lowland	69-39/164	51.643937°N 17.341475°E	1	1	3	3	3,0	51.50 ± 3.536
Czernikowice	Lower Silesia	Silesian Lowlands	75-18/2	51.317389°N 15.871469°E	3	5	24	11	4,8	40.59 ± 16.586
Krzyżowice	Lower Silesia	Silesian Lowlands	82-27/34	50.991784°N 16.881032°E	6	6	29	18	4,8	39.93 ± 7.181
Łazy	Lower Silesia	Silesian Lowlands	71-24/99	51.501889°N 16.550415°E	5	5	32	15	6,4	36.86 ± 10.569
Rolantowice	Lower Silesia	Silesian Lowlands	83-27/50	50.929931°N 16.905221°E	3	3	12	11	4,0	35.27 ± 14.718
Świbie	Silesia	Silesian Upland	92-43/-	50.526735°N 18.528901°E	27	27	121	106	4,5	34.23 ± 10.968
Wrocław-Żerniki	Lower Silesia	Silesian Lowlands	79-27/101	51.134323°N 16.931425°E	3	3	12	7	4,0	34.75 ±9.459
Wtórek	Greater Poland	South Greater Poland Lowland	69-36/51	51.639414°N 17.875440°E	12	12	59	31	4,9	36.02 ± 11.099
Total					60	62	292	202	4,6	36.15 ± 11.403

*Polish Archaeological Record (in Polish – Archeologiczne Zdjęcie Polski)

Table S2. Detailed characteristics of the examined teeth; sequence numbering follows Fig. S2.

No	Site	Grave number	Relative chronology (acc. Montelius)	MNI	Biological sex	Morphological age category	Tooth FDI	TCA ID	Number of analyzed tooth ground sections	Number of analyzed tooth ground sections with countable ILS	Average thickness of the preparation ± SD	Average AEFCt [µm] ± SD	Average ILSc ± SD	Average ILSw [µm] ± SD	Age acc. ILSc	Age acc. ILSw 3,10 µm	Age acc. ILSw 3,21 µm	Age acc. mean ILSw site specific
1	Cieszków	14	V BAP	2	nd	nd	X4/X5	TCA 22	3	3	51,5±3,54	61,20±6,446	22,2±5,91	2,89±0,795	33,2±5,86	30,7±2,12	30,1±2,06	32,2±2,27
2	Czernikowice	22	V BAP	3	nd	Infans I	dm	TCA 92	2	2	32,0±1,41	12,23±2,348	5,3±0,50	2,35±0,677	7,0±0,35	5,9±0,88	5,8±0,85	7,9±1,32
3	Czernikowice	22	V BAP	3	nd	Infans I	dm	TCA 45	3	3	32,0±6,93	14,19±4,059	7,3±1,75	1,98±0,768	9.3±1,44	6,6±1,44	6,4±1,39	8,9±2,17
4	Czernikowice	74	V BAP	2	nd	Infans II	12/22	TCA 46	0	-	not aplicable							
5	Czernikowice	74	V BAP	2	nd	Infans II	31/41	TCA 93	9	2	44,8±19,13	10,04±2,063	4,3±0,50	2,40±0,807	10,0±0,71	8,7±0,83	8,6±0,57	10,4±0,89
6	Czernikowice	82	V BAP	2	F?	Adultus	X3	TCA 47	10	4	41,7±18,03	45,95±15,646	31,2±3,77	1,91±0,326	42,7±4,53	25,3±5,18	24,8±5,01	32,8±7,71
7	Krzyżowice	3 (N10)	nd	1	nd	Juvenis	14/24	TCA 76	9	4	43,0±9,73	29,62±5,018	11,8±3,28	2,66±0,900	22,3±2,87	20,1±1,72	19,7±1,66	23,5±2,33
8	Krzyżowice	5	EIA (HaC)	1	F	Adultus	X6	TCA 73	5	4	37,8±5,81	39,08±10,486	16,5±3,39	2,50±0,600	22,0±3,61	18,1±3,67	17,7±3,55	22,6±4,99
9	Krzyżowice	6	EIA (HaC)	1	M?	Adultus	33/43	TCA 74	5	3	37,6±5,37	54,97±9,704	19,4±3,54	2,50±0,267	28,9±3,07	27,2±3,39	26,6±3,27	33,6±4,60
10	Krzyżowice	22 (N7)	EIA (HaC)	1	F	Adultus/Maturus	18/28	TCA 75	5	3	36,4±3,13	20,25±2,823	12,9±3,18	1,62±0,365	29,3±3,21	21,7±3,02	21,5±2,91	23,6±4,10
11	Krzyżowice	28	EIA (HaC)	2	F?	Adultus/Maturus	31/41	TCA 77	3	2	44,3±3,79	59,20±4,721	26,0±3,40	2,27±0,042	31,5±3,54	24,6±1,59	23,9±1,53	31,5±2,16
12	Krzyżowice	WN	nd	1	nd	Juvenis	X6	TCA 78	2	2	39,5±10,61	58,93±2,916	27,5±3,70	2,14±0,052	33,0±0,71	24,5±0,03	23,9±0,03	31,3±0,04
13	Łazy	1/97	V BAP-HaC	6	M	Adultus	X6/X7	TCA 66	6	2	33,8±4,75	53,45±18,296	19,3±3,66	2,46±1,064	25,8±3,84	22,7±6,69	22,1±6,46	25,9±7,92
14	Łazy	4/95	EIA (HaC)	2	M?	Maturus	33/43	TCA 67	8	1	52,5±8,07	86,71±0,056	34,8±7,17	2,49±0,000	44,3±7,65	37,5±0,00	36,5±0,00	42,6±0,00
15	Łazy	5/97	V BAP	2	F?	Adultus	X4/X5	TCA 68	6	6	32,2±4,79	53,40±9,590	21,5±4,34	2,58±0,785	32,5±4,00	28,2±3,13	27,6±3,03	31,4±3,69
16	Łazy	6/97	V BAP-HaC	2	(F)	Adultus/Maturus	13/23	TCA 69	5	3	32,8±10,43	56,07±5,081	31,2±6,41	2,57±0,390	42,7±6,17	39,3±7,39	38,4±7,14	44,4±8,75
17	Łazy	8/97	V BAP	2	M?	Adultus	14/24	TCA 70	7	3	32,3±7,47	66,16±12,469	21,2±5,94	3,14±0,351	31,7±2,75	37,7±8,84	36,7±8,54	42,7±10,46
18	Rolantowice	19	EIA (HaC)	2	nd	nd	X6/X7	TCA 71	4	4	24,0±3,56	21,96±2,779	10,8±1,67	2,08±0,407	18,8±2,99	15,1±2,79	14,8±2,78	17,5±2,88
19	Rolantowice	20	EIA (HaC)	1	F	Adultus	31	TCA 23	5	4	47,6±12,50	47,25±21,562	35,9±7,26	1,89±0,369	41,4±7,79	31,9±7,60	31,0±7,34	41,1±10,25
20	Rolantowice	21	EIA (HaC)	1	(F)	Adultus	X6/X7	TCA 72	3	3	27,0±9,90	44,33±44,328	16,8±1,60	2,66±0,452	24,8±3,09	22,3±3,05	21,8±3,03	27,3±3,29
21	Świbie	7	EIA (HaC-D)	1	F?	Adultus	11/21	TCA 55	5	5	37,0±9,75	51,36±9,541	14,1±2,51	3,72±0,967	20,1±2,40	22,6±3,15	22,0±3,05	20,1±2,69
22	Świbie	31	EIA (HaC-D)	1	F	Adultus	14	TCA 56	2	2	22,5±0,71	31,03±5,891	11,5±1,00	2,72±0,660	22,0±0,71	20,5±1,83	20,2±1,77	19,0±1,55
23	Świbie	42	EIA (HaC-D)	1	F?	Adultus	X4/X5	TCA 58	4	4	49,3±10,63	56,27±6,445	16,4±2,62	3,63±0,239	27,4±2,49	31,2±3,66	30,5±3,54	28,2±3,12
24	Świbie	132	EIA (HaC-D)	1	(F)	Adultus	15/25	TCA 52	2	2	38,0±24,04	52,02±7,353	16,8±2,22	3,11±0,026	28,3±2,47	28,3±2,34	27,7±2,26	25,8±1,99
25	Świbie	175	EIA (HaC-D)	2	(F)	Adultus	33/43	TCA 60	6	6	29,7±7,89	27,92±1,201	9,0±1,21	3,13±0,323	18,5±0,95	18,5±0,34	18,2±0,33	17,1±0,29
26	Świbie	175	EIA (HaC-D)	2	M?	Adultus	15/25	TCA 64	6	6	38,5±10,58	48,82±10,902	15,7±3,63	3,34±0,309	27,2±3,60	29,7±6,84	29,1±6,61	27,0±5,81
27	Świbie	188	EIA (HaC-D)	1	M?	Adultus	23	TCA 32	8	5	34,1±8,13	65,95±65,945	22,1±5,74	3,57±0,673	33,6±5,33	35,3±8,70	34,5±8,40	31,7±7,39
28	Świbie	194	EIA (HaC-D)	2	F?	Adultus	X3	TCA 33	2	2	37,0±9,90	55,09±5,071	15,8±1,71	3,49±0,140	26,3±1,44	28,3±1,94	27,7±1,90	25,6±1,76
29	Świbie	196	EIA (HaC-D)	2	M?	Adultus	X4/X5	TCA 34	4	4	39,0±2,16	60,34±4,577	19,9±3,23	3,23±0,215	30,9±1,53	33,0±2,04	32,3±1,97	29,7±1,75
30	Świbie	197	EIA (HaC-D)	2	M?	Adultus	X4	TCA 61	4	4	28,8±8,06	52,12±15,099	18,4±4,34	3,67±0,485	28,6±4,73	32,1±5,25	31,4±5,07	28,9±4,46
31	Świbie	211	EIA (HaC-D)	3	(F)	Maturus	X3	TCA 35	6	3	35,4±3,05	116,06±10,008	29,5±5,79	4,09±0,598	40,0±5,20	48,7±2,41	47,4±2,34	42,9±2,13
32	Świbie	226	EIA (HaC-D)	2	(M)	Adultus/Maturus	33/43	TCA 51	6	6	27,5±4,93	66,76±2,900	16,8±2,12	4,10±0,501	26,3±1,89	32,5±1,86	31,7±1,79	29,0±1,58
33	Świbie	248	EIA (HaC-D)	1	(F)	Adultus	X3	TCA 63	7	5	26,9±6,47	65,69±4,573	21,0±2,79	3,64±0,354	31,5±2,85	35,2±4,78	34,3±4,63	31,4±4,10
34	Świbie	252	EIA (HaC-D)	1	M?	adult	X4/X5	TCA 36	4	4	34,0±9,42	38,35±3,048	11,6±1,85	3,34±0,404	22,6±1,62	23,4±0,67	22,9±0,66	21,5±0,64
35	Świbie	263	EIA (HaC-D)	1	F?	Adultus/Maturus	X4/X5	TCA 50	2	2	65,0±14,14	50,49±5,023	15,8±1,50	3,22±0,541	26,8±1,04	27,3±1,46	26,7±1,42	24,8±1,28
36	Świbie	309	EIA (HaC-D)	1	(M)	Adultus/Maturus	X4/X5	TCA 37	9	6	37,6±14,4	44,67±23,658	19,5±4,27	3,72±0,874	30,5±4,17	34,7±7,69	33,9±7,43	31,1±6,54
37	Świbie	365	EIA (HaC-D)	1	(M)	Adultus	X5	TCA 62	3	3	32,7±5,13	63,71±2,382	15,7±2,50	4,11±0,558	27,2±1,89	32,1±0,44	31,3±0,42	29,0±0,37
38	Świbie	371	EIA (HaC-D)	2	F?	Adultus	X3	TCA 38	7	7	36,9±10,49	56,43±10,018	15,8±2,52	3,58±0,464	26,3±2,45	28,7±3,35	28,1±3,24	26,0±2,90

No	Site	Grave number	Relative chronology (acc. Montelius)	MNI	Biological sex	Morphological age category	Tooth FDI	TCA ID	Number of analyzed tooth ground sections	Number of analyzed tooth ground sections with countable ILS	Average thickness of the preparation ± SD	Average AEFCt [μm] ± SD	Average ILSc ± SD	Average ILSw [μm] ± SD	Age acc. ILSc	Age acc. ILSw 3,10 μm	Age acc. ILSw 3,21 μm	Age acc. mean ILSw site specific
39	Świbie	421	EIA (HaC-D)	1	F?	Adultus	X6/X7	TCA 49	3	3	29,3±8,50	65,74±5,378	18,8±4,88	4,01±0,571	26,8±5,34	31,1±3,20	30,3±3,17	27,7±3,08
40	Świbie	424	EIA (HaC-D)	1	F?	Adultus	X3	TCA 59	7	7	26,1±7,78	62,16±10,276	18,4±3,55	3,99±0,347	28,9±2,83	34,1±3,41	33,3±3,30	30,6±2,95
41	Świbie	427	EIA (HaC-D)	3	nd	adult	X4	TCA 48	4	3	31,3±9,29	55,17±3,330	16,2±3,31	3,49±0,650	26,7±2,93	25,6±2,27	25,0±2,27	22,9±2,27
42	Świbie	429	EIA (HaC-D)	1	nd	Infans II	X6	TCA 53	2	2	35,0±2,83	19,26±2,394	5,8±0,50	3,37±0,545	11,3±0,35	11,7±0,63	11,5±0,61	10,8±0,53
43	Świbie	439	EIA (HaC-D)	1	nd	Infans II/Juvenis	X4/X5	TCA 39	3	3	26,5±9,19	38,00±4,289	11,7±1,86	3,28±0,488	22,7±1,25	23,3±1,32	22,8±1,28	21,4±1,16
44	Świbie	458	EIA (HaC-D)	1	F?	Adultus/Maturus	X4/X5	TCA 40	3	-	35,0±4,58	CIFC						
45	Świbie	543	EIA (HaC-D)	1	nd	Infans I	dm	TCA 57	2	2	26,5±3,54	24,83±5,894	8,0±0,82	3,10±0,885	10.0±0.00	10,0±2,28	9,7±2,21	8,8±1,94
46	Świbie	558	EIA (HaC-D)	1	F?	Adultus	X2	TCA 41	4	4	37,8±19,16	61,92±5,420	15,5±2,73	4,10±0,863	22,5±2,54	27,0±1,43	26,3±1,39	24,0±1,25
47	Świbie	562	EIA (HaC-D)	1	M?	Adultus/Maturus	33/43	TCA 54	6	6	35,3±11,11	63,36±8,706	19,3±4,29	4,21±0,585	28,8±3,43	37,0±4,38	36,0±4,23	32,8±3,72
48	Wrocław-Żerniki	11	EIA (HaC)	1	(F)	Adultus	31/41	TCA 79	4	3	39,3±9,91	53,34±8,978	19,8±6,15	2,81±0,500	25,3±5,97	22,7±2,59	22,1±2,5,0	23,6±2,72
49	Wrocław-Żerniki	18	V BAP	1	F?	Adultus	X6/X7	TCA 80	4	3	28,0±5,29	27,08±4,872	12,5±3,31	3,05±0,551	17,9±4,44	16,7±1,82	16,4±1,76	17,2±1,91
50	Wrocław-Żerniki	52	IV-V BAP	1	M?	adult	X8	TCA 81	4	1	37,0±10,36	54,67±2,887	17,5±3,87	2,86±0,000	34,0±1,41	33,6±0,00	33,0±0,00	34,5±0,00
51	Wtórek	514	V BAP	2	M?	adult	35/45	TCA 82	5	2	33,0±5,43	97,93±3,944	29,1±0,85	3,36±0,031	40,6±0,53	51,5±11,95	50,2±11,54	48,5±11,05
52	Wtórek	526	EIA (HaC)	1	(F)	Maturus/Senilis	X3	TCA 42	7	5	36,6±21,03	48,08±8,309	13,3±1,89	3,97±0,597	24,0±1,85	28,6±4,60	28,0±4,46	27,3±4,28
53	Wtórek	542	V BAP	3	nd	Infans II	X6	TCA 83	4	4	30,0±4,08	26,92±4,544	8,5±1,29	3,18±0,503	14,0±1,29	14,2±1,58	13,9±1,53	13,5±1,47
54	Wtórek	556	V BAP	1	(M)	Adultus/Maturus	33/43	TCA 84	4	2	29,3±7,50	69,83±2,095	18,7±0,58	3,78±0,887	28,2±0,58	28,9±13,78	28,2±13,3	27,5±12,75
55	Wtórek	558	EIA (HaC)	1	M?	Maturus/Senilis	33/43	TCA 85	7	1	28,0±7,09	143,46±26,857	27,3±4,27	4,21±0,017	36,8±4,75	49,4±2,47	48,0±2,39	46,4±2,29
56	Wtórek	578	EIA (HaC)	2	nd	Infans II	X4/X5	TCA 43	3	3	40,3±18,01	33,43±4,044	9,5±0,55	3,52±0,474	20,5±0,55	21,8±1,41	21,4±1,37	21,0±1,32
57	Wtórek	582	EIA (HaC)	1	M?	Adultus	31/41	TCA 86	3	1	33,7±8,39	56,77±4,412	22,5±2,52	2,58±0,000	28,0±0,71	26,1±3,20	25,4±3,09	24,5±2,96
58	Wtórek	740	V BAP	1	M?	Adultus/Maturus	X6	TCA 87	8	4	40,9±6,31	62,73±7,072	23,1±6,14	3,10±1,063	28,6±6,47	27,1±1,60	26,3±1,54	25,5±1,48
59	Wtórek	795	nd	1	F?	Adultus	14/24	TCA 88	6	1	42,3±6,02	130,9±44,936	27,8±3,86	3,81±0,000	38,3±4,60	47,3±3,72	46,1±3,53	44,6±3,44
60	Wtórek	798	V BAP	1	(F)	Maturus	X4/X5	TCA 89	3	2	28,0±5,29	32,63±4,834	16,8±0,50	1,87±0,115	27,8±0,65	21,5±0,82	21,2±0,79	20,7±0,76
61	Wtórek	819	IV BAP	1	M?	Adultus	X4/X5	TCA 90	4	1	37,3±7,41	77,4±7,404	23,0±0,71	3,37±0,000	34,0±0,71	36,0±0,00	35,1±0,00	34,1±0,00
62	Wtórek	828	V BAP	2	F?	adult	14/24	TCA 91	5	5	47,0±7,97	19,54±4,127	8,5±1,34	2,34±0,503	19,0±1,37	16,8±1,18	16,6±1,14	16,3±1,09

Abbreviations used in the table:

BAP – Bronze Age Period
EIA (Ha) – Early Iron Age (Hallstatt period)
MNI – Minimum Number of Individuals
F/M – Female/Male
(F)/(M) – probably Female/probably Male
F?/M? – possibly Female/possibly Male
nd – not determined
CIFC – Cellular Intrinsic Fibre Cementum
Infans I – 0–6.9 years
Infans II – 7–13.9 years
Juvenis – 14–21/22 years
Adultus – 21/22–35 years
Maturus – 35.1–50 years
Senilis – >50 years
adult – onset of suture obliteration

Table S3. Age at death assessment of the analysed individuals based on osteological indicators; sequence numbering follows Table S2.

No.	Site	Grave number	TCA ID	Preserved diagnostic elements	Age indicators used	Observation / stage	Method reference	Osteological age estimate	Confidence of age assessment*	Final age category
1	Cieszków	14	TCA 22	Permanent premolar root recovered among the child's skeletal remains	Tooth root development (X4/X5)	Apex completed	Smith, 1991	≥ 12 years	Low	not determined
2	Czernikowice	22	TCA 92	Fragments of cranial vault bones; fragments of the petrous parts of the right and left temporal bones; fragments of deciduous tooth roots; developing permanent tooth germs: FDI 11/21, 12, 22, 24?, 25, 16, 37?; two sacral vertebrae; epiphyses of long bones; proximal epiphysis of the humerus; fragment of the proximal epiphysis of the femur; proximal epiphysis of the right tibia; fragment of the left ischium; fragment of the calcaneus	Unfused epiphyses of long bones; stage of crown development of permanent teeth; stage of root development of deciduous teeth	Deciduous tooth roots – Rc; FDI 11/21 - Crc; FDI 12 and 22 - Crc; FDI 24 and 25? - Cr½; FDI 16 - Crc?; FDI 37? - Coc; unfused epiphyses of long bones: proximal humerus, proximal femur and proximal tibia; unfused left ischium	Cunningham et al., 2016; Smith, 1991; Ubelaker, 1978, pp. 46–47	ca. 4 years ± 1 year	High	Infans I
3	Czernikowice	22	TCA 45	Additional tooth from the same individual (replacement specimen)	As in TCA92	Repeat observation of the same individual	Cunningham et al., 2016; Smith, 1991; Ubelaker, 1978, pp. 46–47	ca. 4 years ± 1 year	High	Infans I
4	Czernikowice	74	TCA 46	Numerous fragments of cranial vault bones; fragment of the petrous part of the right temporal bone; Wormian bone in the lambdoid suture; fragments of permanent tooth roots; crown of a premolar; middle phalanx of the hand; fragments of long bone shafts	Unfused epiphyses of long bones; stage of crown development of permanent teeth	Permanent tooth roots – Rc, A½; premolar crown (X4/X5) – Crc; fragment of a long bone shaft with an unfused epiphysis	Cunningham et al., 2016; Smith, 1991; Ubelaker, 1978, pp. 46–47	ca. 10 years ± 2.5 years	High	Infans II
5	Czernikowice	74	TCA 93	Additional tooth from the same individual (replacement specimen; original specimen dissolved)	As in TCA46	Repeat observation of the same individual	Cunningham et al., 2016; Smith, 1991; Ubelaker, 1978, pp. 46–47	ca. 10 years ± 2.5 years	High	Infans II
6	Czernikowice	82	TCA 47	Numerous fragments of cranial vault bones with well-preserved sutures (coronal, sagittal and lambdoid); fragments of long bones	Degree of cranial suture obliteration; stage of root development of permanent teeth; epiphyseal fusion of long bones	Sagittal suture: segments S2 and S3 fused endocranially, ectocranially open; lambdoid suture open endocranially and ectocranially; coronal suture, segments C1 and C2: in the process of fusion; permanent tooth roots – Ac stage; distal epiphysis of the humerus completely fused; olecranon fused with the shaft of the ulna	Cunningham et al., 2016; Hunger & Rother, 1978; Smith, 1991	ca. 21/22–35 years	Moderate	Adultus
7	Krzyżowice	3 (N10)	TCA 76	Numerous fragments of cranial vault bones with preserved sagittal and lambdoid sutures; permanent tooth roots; fragments of pelvic bones; fragments of long bone shafts and epiphyses	Degree of cranial suture obliteration; stage of root development of permanent teeth; varying stages of epiphyseal fusion in long and pelvic bones	Cranial sutures open endocranially and ectocranially; permanent tooth roots – A½–Ac; proximal humeral shaft without fused head; proximal epiphysis of the humerus unfused; head of the left femur fused; fragments of proximal tibial epiphyses in the process of fusion. Radiographic (X-ray) imaging of the proximal humerus conducted as part of the diagnostic assessment	Cunningham et al., 2016; Hunger & Rother, 1978; Smith, 1991; Ubelaker, 1978, pp. 46–47	ca. 14–17 years	High	Juvenis
8	Krzyżowice	5	TCA 73	Numerous fragments of the cranial vault bones; preserved fragments of the frontal and parietal bones and the squamous part of the occipital bone with cranial sutures; the alveolar process of the left maxilla; the alveolar part of the mandible; two fragments of permanent tooth roots including that of an M1 tooth; fragments of vertebral bodies and arches; fragments of pelvic bones; fragments of the epiphyses and shafts of long bones	Degree of cranial suture obliteration; stage of development of permanent tooth roots; degree of fusion of vertebral elements, pelvic bones and the shafts and epiphyses of long bones	Sagittal suture, segments S2 and S3: endocranially fusing; ectocranially open; coronal suture, segment C2: fusing endocranially; ectocranially open; lambdoid suture: open ectocranially and endocranially; squamous suture: open; alveoli of permanent teeth fully formed and open (most teeth absent from the sockets); permanent tooth roots: Ac stage; fragments of the sacrum fused; pelvic bones fused at the acetabulum; epiphyses fused with the shafts: distal epiphysis of the humerus; proximal epiphysis of the ulna; proximal epiphysis of the radius; epiphyses of anatomically unidentifiable long bones fused with the shafts	Cunningham et al., 2016; Hunger & Rother, 1978; Smith, 1991; Ubelaker, 1978, pp. 46–47	ca. 21/22–35 years	Moderate	Adultus
9	Krzyżowice	6	TCA 74	Numerous fragments of the cranial vault bones; fragments of the parietal bones and the squamous part of the occipital bone; the alveolar part of the mandible; fragments of permanent tooth roots; fragments of pelvic bones; numerous fragments of the shafts and epiphyses of long bones	Degree of cranial suture obliteration; stage of development of permanent tooth roots; degree of fusion of pelvic bones and the shafts and epiphyses of long bones	Sagittal suture, segment S2: endocranially fused; ectocranially open; sagittal suture, segment S4: endocranially fused; ectocranially open; lambdoid suture, segment L2: open endocranially and ectocranially; alveoli fully formed and open; pelvic bones fused at the acetabulum; permanent tooth roots: Ac stage; epiphyses of long bones fused with the shafts: proximal epiphyses and the trochlea and condyles of the humeri; proximal and distal epiphyses of the ulnae; femoral heads fused and distal epiphyses also fused; proximal and distal epiphyses of the tibiae; distal epiphysis of the left fibula	Cunningham et al., 2016; Hunger & Rother, 1978; Smith, 1991; Ubelaker, 1978, pp. 46–47	ca. 25–35 years	Moderate	Adultus

No.	Site	Grave number	TCA ID	Preserved diagnostic elements	Age indicators used	Observation / stage	Method reference	Osteological age estimate	Confidence of age assessment*	Final age category
10	Krzyżowice	22 (N7)	TCA 75	Numerous fragments of the cranial vault bones, including fragments of the frontal bone; fragments of the parietal bones and the squamous part of the occipital bone; the left alveolar process of the maxilla; the left part of the mandible; fragments of permanent tooth roots; fragments of pelvic bones; numerous fragments of the shafts and epiphyses of long bones	Degree of cranial suture obliteration; stage of development of permanent tooth roots; morphology of the auricular surface; degree of epiphyseal fusion in long bones	Sagittal suture, segment S4: endocranially fused; ectocranially open; lambdoid suture, segment L1: beginning to close; fully formed, open alveoli present following teeth FDI 22–26 and 33, 35–38 (tooth 37 lost ante mortem); one maxillary molar preserved in the alveolus (FDI 18 or 28, side indeterminate); permanent tooth roots: Ac stage; auricular surface: phase 4; sternal epiphysis of the clavicle fused; other epiphyses of long bones fused	Cunningham et al., 2016; Hunger & Rother, 1978; Lovejoy et al., 1985; Smith, 1991	ca. 30–40 years	Moderate	Adultus/Maturus
11	Krzyżowice	28	TCA 77	Numerous fragments of the cranial vault bones with the coronal, sagittal and lambdoid sutures preserved; a fragment of the right maxilla; fragments of permanent tooth roots; fragments of pelvic bones; numerous fragments of the shafts and epiphyses of long bones	Degree of cranial suture obliteration; stage of development of permanent tooth roots; degree of epiphyseal fusion in long bones	Sagittal suture, segment S4: endocranially fused; ectocranially open; lambdoid suture, segment L2: beginning to fuse endocranially; ectocranially open; fully formed alveoli of the maxilla (FDI 11–15) and mandible present; permanent tooth roots: Ac stage; ischial tuberosity fused; pelvic bones fused at the acetabulum; epiphyses of long bones fused, in particular the distal epiphysis of the radius, the proximal and distal epiphyses of the femora, and the proximal epiphysis of the tibia	Cunningham et al., 2016; Hunger & Rother, 1978; Smith, 1991	ca. 30–40 years	Moderate	Adultus/Maturus
12	Krzyżowice	BN	TCA 78	Numerous fragments of the cranial vault bones with the coronal, sagittal and lambdoid sutures preserved; fragments of permanent tooth roots; fragments of pelvic bones; numerous fragments of the shafts and epiphyses of long bones	Degree of cranial suture obliteration; stage of development of permanent tooth roots; degree of epiphyseal fusion in long bones	Cranial sutures open; permanent tooth roots: Ac stage; preserved fragments of the acetabula indicating fusion of the ilium, ischium and pubis; proximal epiphysis of the humerus in the process of fusion; fragments of the distal epiphyses of the femora unfused	Cunningham et al., 2016; Hunger & Rother, 1978; Smith, 1991	ca. 15–20 years	High	Juvenis
13	Łazy	1/97	TCA 66	Numerous fragments of the cranial vault bones with the coronal, sagittal and lambdoid sutures preserved; fragments of permanent tooth roots; fragments of the shafts and epiphyses of long bones	Degree of cranial suture obliteration; stage of development of permanent tooth roots; degree of epiphyseal fusion in long bones	Coronal suture, segment C1: beginning of obliteration; sagittal suture, segments S1 and S2: beginning of obliteration; lambdoid suture: open; fully formed and open alveoli in the alveolar part of the mandible following teeth FDI 31–36 and 41–48; permanent tooth roots: Ac stage; epiphyses of long bones fused with the shafts,preserved fragmentarily and anatomically indeterminate	Cunningham et al., 2016; Hunger & Rother, 1978; Smith, 1991	ca. 21/22–35 years	Moderate	Adultus
14	Łazy	4/95	TCA 67	Numerous fragments of the cranial vault bones with the coronal, sagittal and lambdoid sutures preserved; fragments of permanent tooth roots; fragments of the shafts and epiphyses of long bones	Degree of cranial suture obliteration; stage of development of permanent tooth roots; degree of epiphyseal fusion in long bones	Coronal suture, segment C1: endocranially completely obliterated; ectocranially open; sagittal suture, endocranially obliterated along its entire length; ectocranially almost obliterated at segment S2 and obliterated at segment S4; lambdoid suture, segment L1: endocranially obliterated, ectocranially open; permanent tooth roots: Ac stage; epiphyses of long bones fused with the shafts, but not assignable to specific anatomical elements	Cunningham et al., 2016; Hunger & Rother, 1978; Smith, 1991	ca. 35–45 years	Moderate	Maturus
15	Łazy	5/97	TCA 68	Numerous fragments of the cranial vault bones with the coronal, sagittal and lambdoid sutures preserved; numerous fragments of permanent tooth roots and alveolar processes; fragments of the sacrum; fragments of the shafts and epiphyses of long bones	Degree of cranial suture obliteration; dental development and alveolar formation; fusion of sacral elements; degree of epiphyseal fusion in long bones	Coronal suture, segment C2: endocranially fusing; ectocranially open; sagittal suture, segment S4: endocranially fusing; ectocranially open; alveolar processes of the maxillae with fully formed, open alveoli; permanent tooth roots: Ac stage; vertebrae of the sacrum fused; epiphyses fused with the shafts: trochlea of the humerus and proximal epiphyses of the radii, distal epiphysis of the femur, distal epiphysis of the tibia	Cunningham et al., 2016; Hunger & Rother, 1978; Smith, 1991	ca. 25–35 years	Moderate	Adultus
16	Łazy	6/97	TCA 69	Numerous fragments of the cranial vault bones with the coronal, sagittal and lambdoid sutures preserved; fragments of the mandible; fragments of permanent tooth roots; metatarsals; fragments of the shafts and epiphyses of long bones	Degree of cranial suture obliteration; dental development; degree of epiphyseal fusion in metatarsal and long bones	Coronal suture, segment C2: ectocranially open; sagittal suture, segment S3: obliterated; sagittal suture, segment S4: beginning to obliterate endocranially; lambdoid suture, segment L1: beginning of endocranial obliteration; ectocranially open; lambdoid suture, segment L2: beginning of endocranial obliteration; ectocranially open; permanent tooth roots: Ac stage; alveoli of the mandible fully formed and open; metatarsals with fused epiphyses; proximal epiphysis of the left ulna fused; epiphyses of long bones fused with the shafts but not assignable to specific anatomical locations	Cunningham et al., 2016; Hunger & Rother, 1978; Smith, 1991	ca. 30–40 years	Moderate	Adultus/Maturus

No.	Site	Grave number	TCA ID	Preserved diagnostic elements	Age indicators used	Observation / stage	Method reference	Osteological age estimate	Confidence of age assessment*	Final age category
17	Łazy	8/97	TCA 70	Numerous fragments of cranial vault bones; fragments of the alveolar processes of the maxillae; fragments of permanent tooth roots; a clavicle fragment; and numerous fragments of the shafts and epiphyses of long bones	Degree of cranial suture obliteration; dental development; stage of epiphyseal fusion in long bones, including the clavicle	Sagittal suture, segment S3: endocranially obliterated, ectocranially open; permanent tooth roots at Ac stage; alveoli of teeth FDI 11–14 fully developed and open; sternal epiphysis of the clavicle fused; distal femoral epiphysis fused; epiphyses of long bones fused with the diaphyses, although most are not anatomically identifiable	Cunningham et al., 2016; Hunger & Rother, 1978; Smith, 1991	ca. 21/22–35 years	Low	Adultus
18	Rolantowice	19	TCA 71	Fragments of cranial vault bones; a fragment of the alveolar process; fragments of permanent tooth roots; fragments of the shafts and epiphyses of long bones	Degree of cranial suture obliteration; dental development; stage of epiphyseal fusion in long bones	Preserved fragments of cranial sutures, all open, but too small to assign to specific suture segments; permanent tooth roots at Ac stage; alveoli fully developed and open; no diagnostically relevant fragments of long bone diaphyses and epiphyses permitting assessment of fusion, and no articular or fusion surfaces observed among the preserved material	Cunningham et al., 2016; Hunger & Rother, 1978; Smith, 1991	>ca. 15 years ± 3 years	Low	not determined
19	Rolantowice	20	TCA 23	Fragments of cranial bones with preserved segments of the coronal, sagittal and lambdoid sutures; fragment of the mandible with alveoli; permanent tooth roots; fragments of long bones, including the humerus and femur, with preserved epiphyses	Degree of obliteration of cranial sutures; development of permanent tooth roots and mandibular alveoli; degree of epiphyseal fusion in long bones, including the humerus and femur	Coronal suture, segment C1: beginning of endocranial obliteration, ectocranially open; segment C2: ectocranially open; sagittal suture, segment S1: beginning of endocranial obliteration, ectocranially open; segment S2: endocranially almost obliterated, ectocranially partially open; lambdoid suture, segment L1: open endocranially and ectocranially; mandibular alveoli open; permanent tooth roots at Ac stage; epiphyses of long bones fused with the diaphyses, including the proximal and distal humeral epiphyses and the distal femoral epiphysis	Cunningham et al., 2016; Hunger & Rother, 1978; Smith, 1991	ca. 21/22–30 years	Moderate	Adultus
20	Rolantowice	21	TCA 72	Fragments of cranial vault bones; the alveolar part of the mandible; permanent tooth roots; fragments of the shafts and epiphyses of long bones	Degree of obliteration of cranial sutures; development of permanent tooth roots and mandibular alveoli; degree of epiphyseal fusion in long bones	Sagittal suture, segment S3: endocranially obliterated, ectocranially open; lambdoid suture, segment L2: open endocranially and ectocranially; permanent tooth roots at Ac stage; mandibular alveoli fully developed and open; epiphyses of long bones fused with the diaphyses	Cunningham et al., 2016; Hunger & Rother, 1978; Smith, 1991	ca. 21/22–35 years	Low	Adultus
21	Świbie	7	TCA 55	Cranial bones with preserved coronal, sagittal and lambdoid sutures; permanent tooth roots; manual phalanges; proximal radius; long bones with preserved epiphyses	Degree of cranial suture obliteration; development of permanent tooth roots; degree of epiphyseal fusion in manual phalanges, proximal radius and long bones	Coronal suture segment C2 ectocranially open; sagittal suture segment S2 endocranially obliterated and ectocranially open; lambdoid suture segments L1 and L2 open endocranially and ectocranially; permanent tooth roots at Ac stage; epiphyses of the manual phalanges fused with the diaphyses; radial head fused with the diaphysis; epiphyses of long bones fused with the diaphyses	Cunningham et al., 2016; Hunger & Rother, 1978; Smith, 1991	ca. 21/22–35 years	Low	Adultus
22	Świbie	31	TCA 56	Cranial bones with preserved coronal, sagittal and lambdoid sutures; permanent tooth roots; auricular surface of the ilium; proximal radius and ulna; distal humerus; long bones with preserved epiphyses	Degree of cranial suture obliteration; development of permanent tooth roots; morphology of the auricular surface of the ilium; degree of epiphyseal fusion in the proximal radius and ulna, distal humerus and other long bones	Coronal suture segment C1 endocranially in the initial stage of obliteration and ectocranially open; sagittal suture segment S1 endocranially in the initial stage of obliteration and ectocranially open; sagittal suture segment S2 endocranially obliterating and ectocranially open; lambdoid suture segment L2 endocranially obliterating and ectocranially open; permanent tooth roots at Ac stage; auricular surface phase 3; proximal radial and ulnar epiphyses fused with the diaphyses; distal humeral epiphysis fused with the diaphysis; other long-bone epiphyses fused with the diaphysis	Cunningham et al., 2016; Hunger & Rother, 1978; Lovejoy et al., 1985; Smith, 1991	ca. 25–35 years	Moderate	Adultus
23	Świbie	42	TCA 58	Cranial bones with preserved coronal, sagittal and lambdoid sutures; permanent tooth roots; pelvic bones with acetabulum; humerus; proximal radius	Degree of cranial suture obliteration; development of permanent tooth roots; fusion of pelvic bones in the acetabulum; degree of epiphyseal fusion in the humerus and proximal radius	Coronal suture segment C2 endocranially obliterated and ectocranially open; sagittal suture segment S4 endocranially obliterated and ectocranially open; lambdoid suture segment L1 endocranially obliterating and ectocranially open; permanent tooth roots at Ac stage; pelvic bones fused in the acetabulum; proximal and distal humeral epiphyses fused with the diaphysis; radial head fused with the diaphysis	Cunningham et al., 2016; Hunger & Rother, 1978; Smith, 1991	ca. 25–35 years	Moderate	Adultus
24	Świbie	132	TCA 52	Cranial bones with preserved coronal and sagittal sutures; permanent tooth roots; metacarpals and metatarsals; manual phalanges; long bones with preserved epiphyses	Degree of coronal and sagittal suture obliteration; development of permanent tooth roots; degree of epiphyseal fusion in metacarpals, metatarsals, manual phalanges and long bones	Coronal suture segment C1 endocranially obliterating and ectocranially open; sagittal suture segment S2 endocranially obliterated and ectocranially open; permanent tooth roots at Ac stage; epiphyses of metacarpals and metatarsals fused; epiphyses of manual phalanges fused with the diaphyses; epiphyses of long bones fused with the diaphyses	Cunningham et al., 2016; Hunger & Rother, 1978; Smith, 1991	ca. 20–35 years	Low	Adultus

No.	Site	Grave number	TCA ID	Preserved diagnostic elements	Age indicators used	Observation / stage	Method reference	Osteological age estimate	Confidence of age assessment*	Final age category
25	Świbie	175	TCA 60	Cranial bones with preserved sagittal suture; mandible with alveoli; permanent tooth roots; auricular surface of the ilium; proximal femur	Degree of sagittal suture obliteration; development of permanent tooth roots and alveolar morphology; morphology of the auricular surface of the ilium; degree of epiphyseal fusion in the proximal femur	Sagittal suture segment S4 endocranially obliterating and ectocranially open; permanent tooth roots at Ac stage; mandibular alveoli fully developed and open; auricular surface phase 4; femoral head fused	Cunningham et al., 2016; Hunger & Rother, 1978; Lovejoy et al., 1985; Smith, 1991	ca. 21/22–35 years	Moderate	Adultus
26	Świbie	175	TCA 64	Cranial bones with preserved sagittal and lambdoid sutures; permanent tooth roots; auricular surface of the ilium; proximal humerus; long bones with preserved epiphyses	Degree of sagittal and lambdoid suture obliteration; development of permanent tooth roots; morphology of the auricular surface of the ilium; degree of epiphyseal fusion in the proximal humerus and other long bones	Sagittal suture segment S2 endocranially obliterated and ectocranially open; sagittal suture segment S4 endocranially obliterating and ectocranially open; lambdoid suture segment L1 open endocranially and ectocranially; permanent tooth roots at Ac stage; auricular surface phase 3–4; proximal humeral epiphysis fused with the diaphysis; other long-bone epiphyses fused with the diaphyses	Cunningham et al., 2016; Hunger & Rother, 1978; Lovejoy et al., 1985; Smith, 1991	ca. 21/22–35 years	Moderate	Adultus
27	Świbie	188	TCA 32	Cranial bones with preserved coronal and sagittal sutures; spheno-occipital synchondrosis; maxillae and mandible with preserved alveoli; permanent tooth roots; proximal radius; distal femur	Degree of coronal and sagittal suture obliteration; closure of the spheno-occipital synchondrosis; development of permanent tooth roots and alveolar morphology; degree of epiphyseal fusion in the proximal radius and distal femur	Coronal suture segment C1 ectocranially open; sagittal suture segment S1 endocranially obliterated and ectocranially partially open; sagittal suture segment S2 endocranially obliterated; spheno-occipital synchondrosis fused; permanent tooth roots at Ac stage; maxillary alveoli fully developed and open (FDI 11–16, 21–28); mandibular alveoli fully developed and open (FDI 31–38, 41–43); proximal radial epiphysis fused with the diaphysis; distal femoral epiphysis fused with the diaphysis	Cunningham et al., 2016; Hunger & Rother, 1978; Smith, 1991	ca. 21/22–35 years	Moderate	Adultus
28	Świbie	194	TCA 33	Cranial bones with preserved coronal, sagittal and lambdoid sutures; mandible with preserved alveolar part; permanent tooth roots; proximal femur; long bones with preserved epiphyses	Degree of cranial suture obliteration; development of permanent tooth roots and alveolar morphology; degree of epiphyseal fusion in the proximal femur and other long bones	Coronal suture segment C1 endocranially obliterated and ectocranially open; sagittal suture segment S2 endocranially obliterated and ectocranially open; lambdoid suture segment L3 open endocranially and ectocranially; permanent tooth roots at Ac stage; alveoli of the mandibular alveolar part fully developed and open; epiphyses of long bones, including the femoral head, fused with the diaphyses	Cunningham et al., 2016; Hunger & Rother, 1978; Smith, 1991	ca. 21/22–35 years	Moderate	Adultus
29	Świbie	196	TCA 34	Cranial bones with preserved sagittal and lambdoid sutures; mandible with preserved alveoli; permanent tooth roots; distal humerus; distal fibula	Degree of sagittal and lambdoid suture obliteration; development of permanent tooth roots and alveolar morphology; degree of epiphyseal fusion in the distal humerus and distal fibula	Sagittal suture segments S2–S3 endocranially obliterated and ectocranially open; sagittal suture segment S4 endocranially in the process of obliteration and ectocranially open; lambdoid suture segment L1 open endocranially and ectocranially; permanent tooth roots at Ac stage; mandibular alveoli fully developed and open; distal humeral epiphysis fused with the diaphysis; distal fibular epiphysis fused with the diaphysis	Cunningham et al., 2016; Hunger & Rother, 1978; Smith, 1991	ca. 21/22–35 years	Low	Adultus
30	Świbie	197	TCA 61	Cranial bones with preserved sagittal and lambdoid sutures; mandible; permanent tooth roots; long bones with preserved epiphyses	Degree of sagittal and lambdoid suture obliteration; development of permanent tooth roots; degree of epiphyseal fusion in long bones	Sagittal suture segment S4 endocranially in the initial stage of obliteration and ectocranially open; lambdoid suture segment L1 in the initial stage of obliteration; permanent tooth roots at Ac stage; epiphyses of long bones fused with the diaphyses	Cunningham et al., 2016; Hunger & Rother, 1978; Smith, 1991	ca. 21/22–35 years	Low	Adultus
31	Świbie	211	TCA 35	Cranial bones with preserved sagittal and lambdoid sutures; maxillae and mandible with preserved alveoli; permanent tooth roots; manual phalanx; iliac bones with preserved auricular surfaces; proximal femur; long bones with preserved epiphyses; metatarsals	Degree of sagittal and lambdoid suture obliteration; development of permanent tooth roots and alveolar morphology; morphology of the auricular surfaces of the ilia; degree of epiphyseal fusion in the manual phalanx, proximal femur, metatarsals and other long bones	Sagittal suture segments S1–S4 endocranially obliterated and ectocranially partially obliterated (stages 1–2); lambdoid suture segments L1 and L2 endocranially obliterated and ectocranially open; permanent tooth roots at Ac stage; maxillary and mandibular alveoli fully developed and open; manual phalanx with fused basal epiphysis; auricular surfaces phase 5–6; femoral head fused with the diaphysis; other long-bone epiphyses fused with the diaphyses; metatarsal heads fused	Cunningham et al., 2016; Hunger & Rother, 1978; Lovejoy et al., 1985; Martin & Knussmann, 1988, p. 433; Smith, 1991	ca. 35-50 years	Moderate	Maturus
32	Świbie	226	TCA 51	Cranial bones with preserved sagittal and lambdoid sutures; manual phalanges; long bones with preserved epiphyses; permanent tooth roots	Degree of sagittal and lambdoid suture obliteration; development of permanent tooth roots; degree of epiphyseal fusion in manual phalanges and long bones	Sagittal suture segment S2 endocranially obliterated and ectocranially obliterated, stage 1; other sagittal suture segments obliterated endocranially; lambdoid suture segment L2 open endocranially and ectocranially; permanent tooth roots at Ac stage; manual phalanges with fused epiphyses; epiphyses of long bones fused with the diaphyses	Cunningham et al., 2016; Hunger & Rother, 1978; Smith, 1991	ca. 30–40 years	Low	Adultus/Maturus

No.	Site	Grave number	TCA ID	Preserved diagnostic elements	Age indicators used	Observation / stage	Method reference	Osteological age estimate	Confidence of age assessment*	Final age category
33	Świbie	248	TCA 63	Cranial bones with preserved sagittal and lambdoid sutures; maxilla and mandible with preserved alveolar processes; permanent tooth roots; pelvic bones with acetabulum; proximal femora; long bones with preserved epiphyses	Degree of sagittal and lambdoid suture obliteration; development of permanent tooth roots and alveolar morphology; fusion of pelvic bones in the acetabulum; degree of epiphyseal fusion in the proximal femora and other long bones	Sagittal suture segment S2 endocranially obliterated and ectocranially open; sagittal suture segment S4 endocranially obliterating and ectocranially open; lambdoid suture segments L1 and L2 open endocranially and ectocranially; permanent tooth roots at Ac stage; alveoli of the maxillary alveolar process FDI 11–16 (further damaged) and mandibular alveolar part FDI 31–36 and 41–45 (further fragment damaged); pelvic bones fused in the acetabulum; femoral heads fused with the diaphyses; other long-bone epiphyses fused with the diaphyses	Cunningham et al., 2016; Hunger & Rother, 1978; Smith, 1991	ca. 21/22–35 years	Moderate	Adultus
34	Świbie	252	TCA 36	Cranial bones with preserved coronal and sagittal sutures; maxilla with preserved alveolar process; permanent tooth roots; femora with preserved epiphyses	Degree of coronal and sagittal suture obliteration; development of permanent tooth roots and alveolar morphology; degree of epiphyseal fusion in the proximal and distal femora	Coronal and sagittal suture segments open ectocranially; permanent tooth roots at Ac stage; maxillary alveoli fully developed and open (FDI 12–16); proximal and distal femoral epiphyses fused with the diaphyses	Cunningham et al., 2016; Hunger & Rother, 1978; Smith, 1991	>21/22 years	Low	adult
35	Świbie	263	TCA 50	Cranial bones with preserved coronal, sagittal and lambdoid sutures; auricular surface of the ilium; distal femur; long bones with preserved epiphyses	Degree of cranial suture obliteration; morphology of the auricular surface of the ilium; degree of epiphyseal fusion in the distal femur and other long bones	Coronal suture segment C1 ectocranially open; sagittal suture segment S4 endocranially obliterated and ectocranially open; lambdoid suture segment L1 endocranially in the process of obliteration and ectocranially open; auricular surface of the ilium phase 4; distal femoral epiphysis fused; other long-bone epiphyses fused with the diaphyses	Cunningham et al., 2016; Hunger & Rother, 1978; Lovejoy et al., 1985; Smith, 1991	ca. 30–40 years	Moderate	Adultus/Maturus
36	Świbie	309	TCA 37	Cranial bones with preserved sagittal and lambdoid sutures; maxilla with preserved alveolar process; permanent tooth roots; distal humerus; manual phalanges; unidentified long bones with preserved epiphyses	Degree of sagittal and lambdoid suture obliteration; development of permanent tooth roots and cementum deposition; morphology of the maxillary alveolar process; degree of epiphyseal fusion in the distal humerus, manual phalanges and other long bones	Sagittal suture segment S3 endocranially obliterated and ectocranially partially obliterated (stages 1–2); lambdoid suture segments L1–L2 endocranially partially obliterated and ectocranially open; permanent tooth roots at Ac stage with increased apical cementum deposition; maxillary alveolar process with fully developed and open alveoli; distal humeral epiphysis fused with the diaphysis; manual phalanges with fused epiphyses; unidentified long-bone epiphyses fused with the diaphyses	Cunningham et al., 2016; Hunger & Rother, 1978; Martin & Knussmann, 1988, p. 433; Smith, 1991	ca. 30–40 years	Moderate	Adultus/Maturus
37	Świbie	365	TCA 62	Cranial bones with preserved sagittal and lambdoid sutures; auricular surface of the ilium; permanent tooth roots; long bones with preserved epiphyses	Degree of sagittal and lambdoid suture obliteration; morphology of the auricular surface of the ilium; development of permanent tooth roots; degree of epiphyseal fusion in long bones	Sagittal suture segments S1–S4 endocranially obliterated and ectocranially nearly obliterated (stages 1–2); lambdoid suture ectocranially open; auricular surface phase 3; permanent tooth roots at Ac stage; epiphyses of long bones fused with the diaphyses	Cunningham et al., 2016; Hunger & Rother, 1978; Lovejoy et al., 1985; Smith, 1991	ca. 21/22–35 years	Moderate	Adultus
38	Świbie	371	TCA 38	Cranial bones with preserved sagittal and lambdoid sutures; mandible with preserved alveoli; permanent tooth roots; metacarpals; manual phalanges	Degree of sagittal and lambdoid suture obliteration; development of permanent tooth roots and alveolar morphology; degree of epiphyseal fusion in metacarpals and manual phalanges	Sagittal suture segment S1 endocranially and ectocranially obliterated; sagittal suture segment S2 endocranially obliterated and ectocranially partially obliterated (stage 1); lambdoid suture segments L2–L3 open endocranially and ectocranially; permanent tooth roots at Ac stage; mandibular alveoli fully developed and open; metacarpal heads fused; manual phalangeal bases fused	Cunningham et al., 2016; Hunger & Rother, 1978; Martin & Knussmann, 1988, p. 433; Smith, 1991	ca. 21/22–35 years	Low	Adultus
39	Świbie	421	TCA 49	Cranial bones with preserved sagittal suture; permanent teeth; manual phalanges; long bones with preserved epiphyses	Degree of sagittal suture obliteration; development of permanent tooth roots; degree of epiphyseal fusion in manual phalanges and long bones	Sagittal suture segments S1–S4 endocranially obliterated and ectocranially obliterated (stages 1–2); permanent tooth roots at Ac stage; epiphyses of the manual phalanges fused with the diaphyses; epiphyses of long bones fused with the diaphyses	Cunningham et al., 2016; Hunger & Rother, 1978; Smith, 1991	ca. 21/22–35 years	Moderate	Adultus
40	Świbie	424	TCA 59	Cranial bones with preserved coronal, sagittal and lambdoid sutures; permanent tooth roots; manual phalanges; distal femur; long bones with preserved epiphyses	Degree of cranial suture obliteration; development of permanent tooth roots; degree of epiphyseal fusion in manual phalanges, distal femur and other long bones	Coronal suture segment C1 open endocranially and ectocranially; sagittal suture segment S2 endocranially partially obliterated and ectocranially open; lambdoid suture segment L2 open endocranially and ectocranially; permanent tooth roots at Ac stage; middle and distal manual phalanges with fused epiphyses; distal femoral epiphysis fused; other long-bone epiphyses fused with the diaphyses	Cunningham et al., 2016; Hunger & Rother, 1978; Smith, 1991	ca. 21/22–35 years	Low	Adultus

No.	Site	Grave number	TCA ID	Preserved diagnostic elements	Age indicators used	Observation / stage	Method reference	Osteological age estimate	Confidence of age assessment*	Final age category
41	Świbie	427	TCA 48	Cranial bones with preserved lambdoid suture; permanent teeth; Fragments of long bones with preserved epiphyses; manual phalanges	Development of permanent tooth roots; degree of lambdoid suture obliteration; degree of epiphyseal fusion in long bones and manual phalanges	Lambdoid suture, segment L2 endocranially and ectocranially open; permanent tooth roots at Ac stage; epiphyses of long bones fused with the diaphyses; proximal epiphyses of the manual phalanges fused with the diaphyses	Cunningham et al., 2016; Hunger & Rother, 1978; Smith, 1991	>21/22 years	Low	adult
42	Świbie	429	TCA 53	Fragments of cranial vault bones; deciduous and permanent teeth; middle phalanx of the hand	Degree of cranial suture obliteration; development of deciduous and permanent tooth roots; degree of epiphyseal fusion in the manual phalanges	Cranial sutures open endocranially and ectocranially; roots of deciduous and permanent teeth present; middle manual phalanx with unfused epiphysis	Cunningham et al., 2016; Smith, 1991	ca. 7–15 years	Low	Infans II
43	Świbie	439	TCA 39	Permanent teeth; distal humerus; proximal and distal femur; long-bone shafts	Developmental stage of permanent tooth roots; degree of epiphyseal fusion in the distal humerus, proximal and distal femur and other long bones	Permanent tooth roots including canines and premolars at Ac stage; molars at stages R¾–Ac; humeral trochlea unfused; proximal femur with unfused head and trochanters; distal femur with articular surface present and epiphysis unfused; numerous long-bone shafts with unfused epiphyses	Cunningham et al., 2016; Smith, 1991	ca. 13–18 years	Moderate	Infans II/Juvenis
44	Świbie	458	TCA 40	Cranial bones with preserved coronal, sagittal and lambdoid sutures; maxilla with preserved alveoli; permanent tooth roots; sternal end of the clavicle; proximal humerus, ulna and tibia; metatarsal; long bones with preserved epiphyses	Degree of cranial suture obliteration; development of permanent tooth roots and alveolar morphology; fusion of the sternal epiphysis of the clavicle; degree of epiphyseal fusion in the proximal humerus, ulna, tibia, metatarsals and other long bones	Coronal suture segment C1 endocranially obliterated and ectocranially open; sagittal suture endocranially obliterated and ectocranially open; lambdoid suture segment L2 endocranially obliterated and ectocranially open; permanent tooth roots at Ac stage; maxillary alveoli fully developed and open; sternal epiphysis of the clavicle fully fused; proximal humeral, ulnar and tibial epiphyses fused with the diaphyses; metatarsal head fused with the diaphysis	Cunningham et al., 2016; Hunger & Rother, 1978; Smith, 1991	ca. 30–40 years	Low	Adultus/Maturus
45	Świbie	543	TCA 57	Frontal bone with metopic suture; occipital bone; deciduous teeth and developing permanent tooth germ; first metacarpal; iliac bones; femora	Degree of metopic suture closure; fusion of the lateral part of the occipital bone with the squama; development of deciduous tooth roots and permanent premolar crown; presence of a pseudoepiphysis of the first metacarpal; degree of pelvic and femoral epiphyseal fusion	Metopic suture obliterated; lateral part of the occipital bone fused with the squama (fusion with the basioccipital indeterminate due to damage); deciduous tooth roots at Ac stage; developing premolar crown at stage Cr3/4–Crc; pseudoepiphysis of the first metacarpal present; iliac bones unfused; femoral shafts with unfused heads, trochanters and distal epiphyses	Cunningham et al., 2016; Smith, 1991; Ubelaker, 1978, pp. 46–47	ca. 5 years ± 1.5 years	Moderate	Infans I
46	Świbie	558	TCA 41	Cranial bones with preserved coronal and sagittal sutures; permanent tooth roots; long bones with preserved epiphyses	Degree of coronal and sagittal suture obliteration; development of permanent tooth roots; degree of epiphyseal fusion in long bones	Coronal suture segment C1 endocranially beginning to obliterate and ectocranially open; coronal suture segment C2 endocranially obliterated and ectocranially open; sagittal suture segments S1–S2 open endocranially and ectocranially; permanent tooth roots at Ac stage; epiphyses of long bones fused with the diaphyses	Cunningham et al., 2016; Hunger & Rother, 1978; Smith, 1991	ca. 21/22–35 years	Low	Adultus
47	Świbie	562	TCA 54	Cranial bones with preserved sagittal and lambdoid sutures; permanent tooth roots; long bones with preserved epiphyses	Degree of sagittal and lambdoid suture obliteration; development of permanent tooth roots; degree of epiphyseal fusion in long bones	Sagittal suture segment S4 endocranially obliterated and ectocranially open; lambdoid suture segment L1 endocranially in the process of obliteration and ectocranially open; lambdoid suture segment L2 open endocranially and ectocranially; permanent tooth roots at Ac stage; epiphyses of long bones fused with the diaphyses	Cunningham et al., 2016; Hunger & Rother, 1978; Smith, 1991	ca. 25–40 years	Low	Adultus/Maturus
48	Wrocław-Żerniki	11	TCA 79	Fragments of cranial vault bones; fragments of the alveolar process of the maxilla; fragments of permanent tooth roots; fragments of pelvic bones; fragments of the shafts and epiphyses of long bones	Degree of obliteration of cranial sutures; development of permanent tooth roots and maxillary alveoli; degree of epiphyseal fusion in the pelvic bones and long bones	Fragment of the fused spheno-occipital synchondrosis; sagittal suture, segment S2: endocranially almost obliterated, ectocranially open; sagittal suture, segment S4: endocranially fusing, ectocranially open; lambdoid suture, segment L3: open endocranially and ectocranially; permanent tooth roots at Ac stage; maxillary alveoli fully developed and open; epiphysis of the iliac crest fused with the ilium; proximal epiphysis of the humerus fused with the shaft; distal epiphysis of the femur fused with the shaft	Cunningham et al., 2016; Hunger & Rother, 1978; Smith, 1991	ca. 20–35 years	Moderate	Adultus
49	Wrocław-Żerniki	18	TCA 80	Fragments of cranial bones with preserved portions of the coronal, sagittal and lambdoid sutures; fragments of permanent tooth roots; fragments of the shafts and epiphyses of long bones	Degree of obliteration of cranial sutures; development of permanent tooth roots; degree of epiphyseal fusion in long bones	Coronal suture, segment C1: ectocranially open; sagittal suture, segment S4: endocranially almost obliterated, ectocranially open; lambdoid suture, segment L3: open endocranially and ectocranially; permanent tooth roots at Ac stage; epiphyses of long bones fused with the diaphyses	Cunningham et al., 2016; Hunger & Rother, 1978; Smith, 1991	ca. 21/22–35 years	Low	Adultus

No.	Site	Grave number	TCA ID	Preserved diagnostic elements	Age indicators used	Observation / stage	Method reference	Osteological age estimate	Confidence of age assessment*	Final age category
50	Wrocław-Żerniki	52	TCA 81	Fragments of cranial bones; fragments of permanent tooth roots; fragments of the shafts and epiphyses of long bones	Degree of obliteration of cranial sutures; development of permanent tooth roots; degree of epiphyseal fusion in long bones	Sagittal suture, segment S4: endocranially fusing, ectocranially open; permanent tooth roots at Ac stage; epiphyses of long bones fused with the diaphyses	Cunningham et al., 2016; Hunger & Rother, 1978; Smith, 1991	>21/22 years	Low	adult
51	Wtórek	514	TCA 82	Fragments of cranial vault bones; fragments of permanent tooth roots; fragments of the mandible including the alveolar part; fragments of the shafts and epiphyses of long bones	Degree of obliteration of cranial sutures; development of permanent tooth roots and alveoli; degree of epiphyseal fusion in long bones	Lambdoid suture, segment L2: open endocranially and ectocranially; permanent tooth roots at Ac stage; distal femoral epiphysis fused; proximal tibial epiphysis fused; epiphyses of anatomically unidentifiable long bones fused with the diaphyses	Cunningham et al., 2016; Hunger & Rother, 1978; Smith, 1991	>21/22 years	Low	adult
52	Wtórek	526	TCA 42	Fragments of cranial bones with preserved portions of the coronal, sagittal and lambdoid sutures; fragment of the mandible with alveoli; permanent tooth roots; fragments of pelvic bones; fragments of the humerus, ulna, radius and femur with preserved epiphyses	Degree of obliteration of cranial sutures; development of permanent tooth roots and mandibular alveoli; morphology of the auricular surface of the ilium; degree of epiphyseal fusion in long bones	Coronal suture, segment C2: endocranially fused, ectocranially open; sagittal suture, segment S1: obliterated; lambdoid suture, segments L1 and L2: endocranial obliteration, ectocranially open; permanent tooth roots at Ac stage; mandibular alveoli (FDI 44–38) fully developed and open; auricular surface of the ilium: phase 6–7; proximal and distal epiphyses of the humerus fused; proximal epiphysis of the ulna fused; proximal epiphysis of the radius fused; distal femoral epiphysis fused	Cunningham et al., 2016; Hunger & Rother, 1978; Lovejoy et al., 1985; Smith, 1991	ca. 45–59 years	Moderate	Maturus/Senilis
53	Wtórek	542	TCA 83	Fragments of cranial bones; fragments of permanent tooth roots; fragments of the shafts and epiphyses of long bones; fragments of metatarsals	Degree of cranial suture obliteration; development of permanent molar roots; stage of epiphyseal fusion in the tibia, long bones and metatarsals	Cranial sutures open; two fragments of permanent molar roots fully developed (Ac stage); proximal epiphysis of the tibia unfused; proximal epiphysis at the base of the first metatarsal unfused; distal (head) epiphyses of the metatarsals unfused; epiphyses of anatomically unidentifiable long bones unfused	Cunningham et al., 2016; Smith, 1991; Ubelaker, 1978, pp. 46–47	ca. 7–14 years	High	Infans II
54	Wtórek	556	TCA 84	Fragments of cranial bones with preserved portions of the sagittal and lambdoid sutures; fragments of the mandible and maxilla with alveoli; permanent tooth roots; fragments of pelvic bones including the auricular surface; fragments of the humerus, ulna and radius with preserved epiphyses	Degree of cranial suture obliteration; development of permanent tooth roots and alveoli; morphology of the auricular surface of the ilium; degree of epiphyseal fusion in long bones	Sagittal suture, segment S1: endocranially obliterated, ectocranially open; lambdoid suture, segment L3: open endocranially and ectocranially; permanent tooth roots at Ac stage; mandibular and maxillary alveoli fully developed and open; auricular surface of the ilium: phase 4; epiphyses of the humerus, ulna and radius fused to the diaphyses	Cunningham et al., 2016; Hunger & Rother, 1978; Lovejoy et al., 1985; Smith, 1991	ca. 30–40 years	Moderate	Adultus/Maturus
55	Wtórek	558	TCA 85	Fragments of cranial bones with preserved portions of the coronal, sagittal and lambdoid sutures; fragments of permanent tooth roots; fragments of long bones	Degree of cranial suture obliteration; development of permanent tooth roots; degree of epiphyseal fusion in long bones	Coronal suture, segment C2: endocranially obliterated, ectocranially partially obliterated; sagittal suture, segment S4: completely obliterated endocranially, ectocranially open; lambdoid suture, segment L1: completely obliterated endocranially and ectocranially (stage 4); permanent tooth roots at Ac stage; epiphyses of long bones fused with the diaphyses, including the proximal epiphyses of the radius and tibia; remaining fused epiphyses not anatomically identifiable	Cunningham et al., 2016; Hunger & Rother, 1978; Martin & Knussmann, 1988, p. 433; Smith, 1991	ca. 40–55 years	Moderate	Maturus/Senilis
56	Wtórek	578	TCA 43	Fragment of the mandible with preserved alveoli; fragments of premolar crowns and developing third molar germs; fragments of the ulna and left radius with preserved distal epiphyses; fragment of the ilium including the iliac crest; fragment of the tibia with the proximal epiphysis	Development of permanent teeth and mandibular alveoli; stage of crown formation of premolars and third molars; degree of fusion of the iliac crest epiphysis; degree of epiphyseal fusion in the ulna, radius and tibia	Mandibular alveoli preserved for teeth FDI 31–35 and 41–47, with a crypt of tooth 48; fragments of premolar crowns at Crc stage; developing third molar germ (FDI 18) at Crc stage; fragment of the ulnar shaft with the distal epiphysis unfused; fragment of the left radial shaft with the distal epiphysis unfused; iliac crest epiphysis unfused; proximal epiphysis of the tibia unfused	Cunningham et al., 2016; Smith, 1991; Ubelaker, 1978, pp. 46–47	ca. 10–12 years	Moderate	Infans II
57	Wtórek	582	TCA 86	Fragments of cranial bones with preserved portions of the sagittal suture; fragments of the maxilla with alveoli (FDI 11–15); fragments of the mandible with alveoli (FDI 31–38 and 42–46, partially damaged); permanent tooth roots; fragments of the ulnae and radii with preserved epiphyses	Degree of cranial suture obliteration; development of permanent tooth roots and alveoli; degree of epiphyseal fusion in the ulnae and radii	Sagittal suture, segment S2: endocranially obliterated, ectocranially open; other preserved cranial sutures ectocranially open; permanent tooth roots at Ac stage; maxillary alveoli (FDI 11–15) fully developed and open; mandibular alveoli open for teeth FDI 31–38 and 42–46 (47-48 partially damaged); epiphyses of the ulnae and radii fused with the diaphyses	Cunningham et al., 2016; Hunger & Rother, 1978; Smith, 1991	ca. 21/22–35 years	Low	Adultus

No.	Site	Grave number	TCA ID	Preserved diagnostic elements	Age indicators used	Observation / stage	Method reference	Osteological age estimate	Confidence of age assessment*	Final age category
58	Wtórek	740	TCA 87	Fragments of cranial bones with preserved portions of the sagittal and lambdoid sutures; permanent tooth roots; hand phalanges; fragments of long bones including the radii with preserved epiphyses	Degree of cranial suture obliteration; development of permanent tooth roots; degree of epiphyseal fusion in hand phalanges and long bones	Sagittal suture, segment S4: endocranially obliterated, ectocranially obliterated (stage 1); lambdoid suture, segment L1: endocranially obliterated, ectocranially open; permanent tooth roots at Ac stage; epiphyses at the bases of the hand phalanges fused with the shafts; epiphyses of long bones fused with the diaphyses, including the radial epiphyses	Cunningham et al., 2016; Hunger & Rother, 1978; Martin & Knussmann, 1988, p. 433; Smith, 1991	ca. 30–40 years	Moderate	Adultus/Maturus
59	Wtórek	795	TCA 88	Fragments of cranial bones with preserved portions of the sagittal suture; permanent tooth roots; fragments of long bones with preserved epiphyses	Degree of cranial suture obliteration; development of permanent tooth roots; degree of epiphyseal fusion in long bones	Sagittal suture, segment S2: ectocranially open; permanent tooth roots at Ac stage; epiphyses of long bones fused with the diaphyses	Cunningham et al., 2016; Hunger & Rother, 1978; Smith, 1991	ca. 20–35 years	Low	Adultus
60	Wtórek	798	TCA 89	Fragments of cranial bones with preserved portions of the coronal, sagittal and lambdoid sutures; permanent tooth roots; fragments of long bones including the tibia with preserved epiphyses	Degree of cranial suture obliteration; development of permanent tooth roots; degree of epiphyseal fusion in long bones, including the proximal tibial epiphysis	Coronal suture, segment C1: endocranially obliterated, ectocranially open; sagittal suture: ectocranially open; lambdoid suture, segment L1: endocranially obliterated, ectocranially open; permanent tooth roots at Ac stage; epiphyses of long bones fused with the diaphyses; proximal tibial epiphysis fused with the shaft	Cunningham et al., 2016; Hunger & Rother, 1978; Smith, 1991	ca. 35–45 years	Moderate	Maturus
61	Wtórek	819	TCA 90	Fragments of cranial bones with preserved portions of the coronal and sagittal sutures; permanent tooth roots; proximal epiphysis of the tibia; radial head; fragments of long bones with preserved epiphyses	Degree of cranial suture obliteration; development of permanent tooth roots; degree of epiphyseal fusion in long bones, including the proximal tibial epiphysis and the radial head	Coronal suture, segment C1: endocranially obliterated, ectocranially open; sagittal suture, segment S1: endocranially and ectocranially open; permanent tooth roots at Ac stage; radial head fused; proximal tibial epiphysis fused; other epiphyses of long bones fused with the diaphyses	Cunningham et al., 2016; Hunger & Rother, 1978; Smith, 1991	ca. 20–30 years	Low	Adultus
62	Wtórek	828	TCA 91	Mandibular fragment; distal femur; fragments of long bones with preserved epiphyses and diaphyses	Development of permanent dentition; degree of epiphyseal fusion in long bones	Mandibular body with alveoli of teeth FDI 31–33 and 41–42, fully developed with open sockets; permanent tooth roots at Ac stage; distal femoral epiphysis fused; epiphyses and diaphyses of unidentifiable long bones fused	Cunningham et al., 2016; Hunger & Rother, 1978; Smith, 1991	>21/22 years	Low	adult

*Confidence reflects the robustness of the osteological assessment based on the number, preservation and diagnostic value of the observed indicators; it does not represent a statistical posterior probability.

Abbreviations used in the table:

Ci — Cusp initiation
Cco — Cusp coalescence
Coc — Crown outline complete
Cr ½ — Crown one half
Cr ¾ — Crown three-fourths
Crc — Crown complete
Ri — Root initiated
Rcl — Root cleft present
R ¼ — Root one-fourth
R ½ — Root one-half
R ⅔ — Root two-thirds
R ¾ — Root three-fourths
Rc — Root complete
A ½ — Root apex half closed
Ac — Root apex closed

Table S4. Osteological sex assessment of the analysed individuals; sequence numbering follows Table S2.

No.	Site	Grave number	TCA ID	Preserved diagnostic elements	Sex traits assessed	Trait assessment	Method reference	Sex estimation	Confidence of sex assessment (%)*	Final sex assessment
1	Cieszków	14	TCA 22	Sexually dimorphic elements not preserved	None	Not applicable	Not applicable	not determined	Not applicable	not determined
2	Czernikowice	22	TCA 92	Sexually dimorphic elements not preserved	None	Not applicable	Not applicable	not determined	Not applicable	not determined
3	Czernikowice	22	TCA 45	Additional tooth from the same individual as TCA 92 (replacement specimen)	None	Repeat observation of the same individual - TCA 92	Not applicable	not determined	Not applicable	not determined
4	Czernikowice	74	TCA 46	Sexually dimorphic elements not preserved	None	Not applicable	Not applicable	not determined	Not applicable	not determined
5	Czernikowice	74	TCA 93	Additional tooth from the same individual as TCA 46 (replacement specimen)	None	Repeat observation of the same individual - TCA 46	Not applicable	not determined	Not applicable	not determined
6	Czernikowice	82	TCA 47	Numerous fragments of cranial bones; squamous part of the occipital bone; fragments of temporal bones; fragments of the mandible; dens of the axis (C2)	Occipital bone morphology; mastoid processes; mandibular condylar process; size of the dens (odontoid process) of C2	Occipital (nuchal crest): gracile (female morphology), score 1–2; mastoid process: small (female morphology; partially damaged), score 1–2; mandibular condylar process: small (female morphology); odontoid process (dens of the axis): small (female morphology)	Buikstra & Ubelaker, 1994, pp. 15–21; Cavazzuti et al., 2019; Piontek, 1985, pp. 210–215; Strzałko et al., 1973	Possibly female	65%	F?
7	Krzyżowice	3 (N10)	TCA 76	Numerous fragments of cranial bones; numerous pelvic fragments including iliac blades; humeral and femoral heads	Morphology of the frontal bone and occipital squama; size of the humeral and femoral heads	Glabella: flat, gracile (female morphology), score 1–2; nuchal crest: gracile (female morphology), score 1–2; greater sciatic notch: score 2–3 (intermediate morphology); humeral and femoral heads: medium size (intermediate morphology)	Buikstra & Ubelaker, 1994, pp. 15–21; Cavazzuti et al., 2019; Piontek, 1985, pp. 210–215; Strzałko et al., 1973	Possibly female	Not applicable	not determined; subadult individual
8	Krzyżowice	5	TCA 73	Numerous fragments of cranial bones, including fragments of the mandible, fragments of the pelvic bones	Morphology of the frontal bone, parietal bones and the squamous part of the occipital bone; the angle of the mandible; fragments of the pelvic bones without preserved diagnostic features	Glabella: gracile (female morphology), score 1–2; supra-orbital margin: sharp (female morphology), score 1; nuchal crest: gracile (female morphology), score 1–2; mandibular angle: smooth (female morphology)	Buikstra & Ubelaker, 1994, pp. 15–21; Piontek, 1985, pp. 210–215; Strzałko et al., 1973	Female	80%	F
9	Krzyżowice	6	TCA 74	Numerous fragments of cranial bones, including fragments of the mandible, fragments of long bones	Morphology of the squamous part of the occipital bone; size of the mandibular condylar process; size of the proximal epiphyses of the humerus and the humeral trochleae; size of the proximal epiphyses of the femora	Nuchal crest: pronounced, score 5; mandibular condylar process: robust (male morphology); humeral head and trochlea: robust (male morphology); femoral head: large (male morphology)	Buikstra & Ubelaker, 1994, pp. 15–21; Cavazzuti et al., 2019; Piontek, 1985, pp. 210–215; Strzałko et al., 1973	Possibly male	60%	M?
10	Krzyżowice	22 (N7)	TCA 75	Numerous fragments of cranial bones and the mandible; fragments of pelvic bones; fragments of long bones	Morphology of the frontal bone and the occipital bone; morphology of the mandible and the size of the mandibular condylar processes; morphology of the preserved fragment of the ilium; size of the epiphyses of long bones, including the humeral trochlea and the femoral head	Supraorbital ridges weakly developed (almost absent); supra-orbital margin very sharp, score 1; nuchal crest smooth, score 1; mandibular angle smooth, without a pronounced masseteric tuberosity; mandibular condylar process small (female morphology); auricular surface (female-like); humeral trochlea small (female morphology); femoral head small (female morphology)	Buikstra & Ubelaker, 1994, pp. 15–21; Cavazzuti et al., 2019; Piontek, 1985, pp. 210–215; Strzałko et al., 1973; Wescott, 2015	Female	87%	F
11	Krzyżowice	28	TCA 77	Numerous fragments of cranial bones and the mandible; fragments of long bones	Morphology of the squamous part of the occipital bone; size of the mastoid processes of the temporal bones; left zygomatic bone; morphology of the mandible and size of the condylar process; size of the femoral heads	Nuchal crest: smooth, score 1 (female morphology); mastoid processes: small, score 1–2 (female morphology); left zygomatic bone: gracile, marginal tubercle absent (female morphology); mandibular angle: smooth, without a pronounced masseteric tuberosity (female morphology); mandibular condylar processes: small (female morphology); femoral heads: small (female morphology)	Buikstra & Ubelaker, 1994, pp. 15–21; Cavazzuti et al., 2019; Piontek, 1985, pp. 210–215; Strzałko et al., 1973	Possibly female	70%	F?
12	Krzyżowice	BN	TCA 78	Numerous fragments of cranial vault bones and the mandible; fragments of long bones	Morphology of the squamous part of the frontal and occipital bones; fragment of the right zygomatic bone; fragments of the right mandible; size of the humeral and femoral heads	Frontal squama preserved too fragmentarily for assessment of the glabella (indeterminate); occipital surface robust (male morphology), external occipital protuberance pronounced, score 5 (male morphology); zygomatic bone large (male morphology); fragments of the mandibular rami robust (male morphology); humeral head medium in size (intermediate morphology); femoral head medium in size (intermediate morphology)	Buikstra & Ubelaker, 1994, pp. 15–21; Cavazzuti et al., 2019; Piontek, 1985, pp. 210–215; Strzałko et al., 1973	Possibly male	Not applicable	not determined; subadult individual

No.	Site	Grave number	TCA ID	Preserved diagnostic elements	Sex traits assessed	Trait assessment	Method reference	Sex estimation	Confidence of sex assessment (%)*	Final sex assessment
13	Łazy	1/97	TCA 66	Numerous fragments of cranial vault bones	Morphology of the frontal bone; morphology of the squamous part of the occipital bone; morphology of the mastoid processes of the temporal bones	Supra-orbital margin rounded, score 4–5 (male morphology); nuchal crest pronounced, score 4 (male morphology); mastoid processes large, score 4 (male morphology)	Buikstra & Ubelaker, 1994, pp. 15–21; Piontek, 1985, pp. 210–215; Strzałko et al., 1973	Male	80%	M
14	Łazy	4/95	TCA 67	Fragments of cranial vault bones; fragment of the C2 vertebra (axis)	Morphology of the frontal bone; morphology of the odontoid process (dens) of the axis	Glabella: score 4 (male morphology); supra-orbital margin: score 4 (male morphology); odontoid process (dens) of the axis (intermediate morphology)	Buikstra & Ubelaker, 1994, pp. 15–21; Cavazzuti et al., 2019; Piontek, 1985, pp. 210–215; Strzałko et al., 1973	Possibly male	65%	M?
15	Łazy	5/97	TCA 68	Fragments of cranial bones; fragment of a cervical vertebra; fragments of the humerus; fragments of the radii	Morphology of the squamous part of the occipital bone; morphology of the mastoid process of the temporal bone; morphology of the right zygomatic bone; size of the axis (C2), including the odontoid process; size of the humeral trochlea; size of the radial heads	Occipital bone: score 1 (female morphology); mastoid process: score 2 (female morphology); axis (C2): small (female morphology); zygomatic bone small, without a marginal process (female morphology); humeral trochlea large (male morphology); radial head small (female morphology)	Buikstra & Ubelaker, 1994, pp. 15–21; Cavazzuti et al., 2019; Piontek, 1985, pp. 210–215; Strzałko et al., 1973	Possibly female	65%	F?
16	Łazy	6/97	TCA 69	Fragments of cranial bones, including elements of the frontal, occipital, zygomatic and mandibular regions	Morphology of the frontal bone and supraorbital region; morphology of the squamous part of the occipital bone; morphology of the zygomatic bone; morphology of the mandible; size of the mandibular condyle	Supraorbital ridges weakly developed (female morphology); supra-orbital margin sharp, score 1 (female morphology); occipital squama moderately developed, score 3 (intermediate morphology); zygomatic bone small, without a marginal tubercle (female morphology); mandibular condyle small (female morphology); mental eminence gracile, score 1–2 (female morphology)	Buikstra & Ubelaker, 1994, pp. 15–21; Cavazzuti et al., 2019; Piontek, 1985, pp. 210–215; Strzałko et al., 1973	Probably female	75%	(F)
17	Łazy	8/97	TCA 70	Fragments of cranial bones and the mandible; fragments of the axis (C2)	Morphology of the occipital bone; size of the condylar process of the mandible; size of the odontoid process (dens) of C2	External occipital protuberance and nuchal lines robust (male morphology); condylar process of the mandible large (male morphology); odontoid process (dens) of the axis large (male morphology)	Buikstra & Ubelaker, 1994, pp. 15–21; Cavazzuti et al., 2019; Piontek, 1985, pp. 210–215; Strzałko et al., 1973	Possibly male	65%	M?
18	Rolantowice	19	TCA 71	Numerous fragments of cranial bones	Morphology of the frontal and occipital bones	Fragments of the frontal and occipital bones preserved too fragmentarily for detailed morphological assessment; preserved portions gracile, without pronounced muscular attachments or bony relief	Buikstra & Ubelaker, 1994, pp. 15–21	not determined	0%	not determined
19	Rolantowice	20	TCA 23	Numerous fragments of cranial bones and the mandible; fragments of pelvic bones; fragments of the humerus	Morphology of the frontal bone; morphology of the zygomatic bones; morphology of the mandible and size of the condylar processes; pelvic bone morphology and degree of opening of the greater sciatic notch; size of the humeral trochlea	Glabella gracile, score 1 (female morphology); zygomatic bones small, without marginal tubercles (female morphology); mental eminence small, score 1–2 (female morphology); condylar process of the mandible small (female morphology); greater sciatic notch wide, score 2 (female morphology); humeral trochlea small (female morphology)	Buikstra & Ubelaker, 1994, pp. 15–21; Cavazzuti et al., 2019; Piontek, 1985, pp. 210–215; Strzałko et al., 1973	Female	85%	F
20	Rolantowice	21	TCA 72	Numerous fragments of cranial bones and the mandible	Morphology of the frontal bone; morphology of the mastoid processes of the temporal bones; morphology of the zygomatic bones; morphology of the mandibular angles and size of the condylar processes	Frontal bone smooth, without pronounced supraorbital ridges (female morphology); mastoid processes small, score 2 (female morphology); zygomatic bones small, without a marginal tubercle (female morphology); mandibular angles smooth, without marked muscular attachments (female morphology); condylar processes of the mandible small (female morphology)	Buikstra & Ubelaker, 1994, pp. 15–21; Cavazzuti et al., 2019; Piontek, 1985, pp. 210–215; Strzałko et al., 1973	Probably female	75%	(F)
21	Świbie	7	TCA 55	Morphology of the supraorbital region; size of the dens of the axis (C2); size of the radial head	Morphology of the supraorbital region; size of the dens of the axis (C2); size of the radial head	Supraorbital ridges gracile (female morphology); dens of the axis small (female morphology); radial head small (female morphology)	Buikstra & Ubelaker, 1994, pp. 15–21; Cavazzuti et al., 2019; Piontek, 1985, pp. 210–215; Strzałko et al., 1973	Possibly female	65%	F?
22	Świbie	31	TCA 56	Cranial bones including the supraorbital region; axis (C2); pelvic bone with preserved greater sciatic notch, auricular surface and preauricular sulcus; distal humerus; proximal radius	Morphology of the supraorbital margin; size of the axis (C2) including the dens; morphology of the greater sciatic notch, auricular surface and preauricular sulcus; size of the humeral trochlea; size of the radial head	Supraorbital margin sharp, score 1 (female morphology); axis (C2) small including the dens (female morphology); greater sciatic notch wide, score 2 (female morphology); auricular surface with preauricular sulcus present and well developed (female morphology); humeral trochlea small (female morphology); radial head small (female morphology)	Buikstra & Ubelaker, 1994, pp. 15–21; Cavazzuti et al., 2019; Piontek, 1985, pp. 210–215; Strzałko et al., 1973; Wescott, 2015	Female	90%	F
23	Świbie	42	TCA 58	Frontal bone; occipital bone; pelvic fragments with preserved greater sciatic notch; proximal and distal humerus	Morphology of the glabella; development of the supraorbital ridge; morphology of the nuchal lines; morphology of the greater sciatic notch; size of the humeral head and humeral trochlea	Glabella gracile, score 2 (female morphology); supraorbital ridge moderately developed (intermediate morphology); nuchal lines minimally expressed, score 1 (female morphology); greater sciatic notch wide, score 2 (female morphology); humeral head small (female morphology); humeral trochlea small (female morphology)	Buikstra & Ubelaker, 1994, pp. 15–21; Cavazzuti et al., 2019; Piontek, 1985, pp. 210–215; Strzałko et al., 1973	Possibly female	70%	F?

No.	Site	Grave number	TCA ID	Preserved diagnostic elements	Sex traits assessed	Trait assessment	Method reference	Sex estimation	Confidence of sex assessment (%)*	Final sex assessment
24	Świbie	132	TCA 52	Cranial bones including the supraorbital region and zygomatic bones; distal humeri	Morphology of the supraorbital margin and supraorbital region; morphology of the frontal process of the zygomatic bone; size of the humeral trochleae	Supraorbital margin sharp, score 2 (female morphology); supraorbital ridges gracile (female morphology); frontal process of the zygomatic bone narrow without a marginal tubercle (female morphology); humeral trochleae small (female morphology)	Buikstra & Ubelaker, 1994, pp. 15–21; Cavazzuti et al., 2019; Piontek, 1985, pp. 210–215; Strzałko et al., 1973	Probably female	75%	(F)
25	Świbie	175	TCA 60	Cranial bones including the supraorbital region and left zygomatic bone; right pelvic bone with preserved auricular surface and preauricular sulcus; proximal femur	Morphology of the supraorbital margin and supraorbital region; morphology of the zygomatic bone and frontal process of the zygomatic bone; morphology of the auricular surface and preauricular sulcus; size of the femoral head	Supraorbital margin sharp, score 2 (female morphology); supraorbital region weakly developed (female morphology); left zygomatic bone small, frontal process narrow (female morphology); auricular surface with preauricular sulcus present (female morphology); femoral head small (female morphology)	Buikstra & Ubelaker, 1994, pp. 15–21; Cavazzuti et al., 2019; Piontek, 1985, pp. 210–215; Strzałko et al., 1973; Wescott, 2015	Probably female	80%	(F)
26	Świbie	175	TCA 64	Proximal humerus; right pelvic bone with preserved auricular surface	Size of the humeral head; morphology of the auricular surface of the ilium	Humeral head very large (male morphology); auricular surface robust (male morphology)	Buikstra & Ubelaker, 1994, pp. 15–21; Cavazzuti et al., 2019; Piontek, 1985, pp. 210–215; Strzałko et al., 1973; Wescott, 2015	Possibly male	70%	M?
27	Świbie	188	TCA 32	Zygomatic bone; mandible; proximal radius	Morphology of the frontal process of the zygomatic bone; morphology of the mental eminence; size of the radial head	Frontal process of the zygomatic bone broad (male morphology); mental eminence prominent, score 4 (male morphology); radial head large (male morphology)	Buikstra & Ubelaker, 1994, pp. 15–21; Cavazzuti et al., 2019; Piontek, 1985, pp. 210–215; Strzałko et al., 1973	Possibly male	70%	M?
28	Świbie	194	TCA 33	Mandible; axis (C2); proximal femur	Morphology of the mandibular angle and masseteric tuberosity; morphology of the odontoid process of the axis (C2); size of the femoral head	Mandibular angle gracile without a pronounced masseteric tuberosity (female morphology); odontoid process of the axis small (female morphology); femoral head small (female morphology)	Buikstra & Ubelaker, 1994, pp. 15–21; Cavazzuti et al., 2019; Piontek, 1985, pp. 210–215; Strzałko et al., 1973	Possibly female	70%	F?
29	Świbie	196	TCA 34	Frontal bone; occipital bone; mandible; distal humerus	Morphology of the glabella; morphology of the nuchal lines; mental eminence; size of the humeral trochlea	Glabella, score 3 (intermediate morphology); nuchal lines pronounced, score 3–4 (male-leaning morphology); mental eminence prominent, score 4 (male morphology); humeral trochlea large (male morphology)	Buikstra & Ubelaker, 1994, pp. 15–21; Cavazzuti et al., 2019; Piontek, 1985, pp. 210–215; Strzałko et al., 1973	Possibly male	70%	M?
30	Świbie	197	TCA 61	Cranial bones including the supraorbital region and occipital bone; zygomatic bones; axis (C2)	Morphology of the supraorbital margin; external occipital protuberance and nuchal lines; zygomatic bones and frontal processes of the zygomatic bones; size of the dens of the axis (C2)	Supraorbital margin rounded, score 3–4 (male morphology); external occipital protuberance and nuchal lines pronounced, score 3–4 (male morphology); zygomatic bones large (male morphology), frontal processes of the zygomatic bones broad with a pronounced marginal tubercle (male morphology); dens of the axis large (male morphology)	Buikstra & Ubelaker, 1994, pp. 15–21; Cavazzuti et al., 2019; Piontek, 1985, pp. 210–215; Strzałko et al., 1973	Possibly male	70%	M?
31	Świbie	211	TCA 35	Cranial bones including the supraorbital region, temporal bones and zygomatic bone; axis (C2); pelvic fragments with preserved auricular surface and preauricular sulcus; proximal femur	Morphology of the supraorbital margin; size of the mastoid processes; morphology of the zygomatic bone and frontal process; size of the dens of the axis (C2); morphology of the auricular surface and preauricular sulcus; size of the femoral head	Supraorbital margin sharp, score 2 (female morphology); mastoid processes small, score 1–2 (female morphology); zygomatic bone small with a narrow frontal process (female morphology); dens of the axis small (female morphology); auricular surface with preauricular sulcus present (female morphology); femoral head small (female morphology)	Buikstra & Ubelaker, 1994, pp. 15–21; Cavazzuti et al., 2019; Piontek, 1985, pp. 210–215; Strzałko et al., 1973; Wescott, 2015	Probably female	85%	(F)
32	Świbie	226	TCA 51	Cranial bones including the frontal bone, supraorbital region and zygomatic bones; mandible; proximal radius	Morphology of the glabella, supraorbital region and supraorbital margins; mental eminence; frontal process of the zygomatic bone; mandibular condylar process; size of the radial head	Glabella pronounced, score 4 (male morphology); supraorbital ridges pronounced (male morphology); supraorbital margins rounded, score 5 (male morphology); mental eminence prominent, score 4 (male morphology); frontal process of the zygomatic bone broad with a pronounced marginal tubercle (male morphology); mandibular condylar process very large (male morphology); radial head large (male morphology)	Buikstra & Ubelaker, 1994, pp. 15–21; Cavazzuti et al., 2019; Piontek, 1985, pp. 210–215; Strzałko et al., 1973	Probably male	80%	(M)
33	Świbie	248	TCA 63	Occipital bone; mandible; axis (C2); proximal femur	Morphology of the occipital squama, external occipital protuberance and nuchal lines; mental eminence; size of the mandibular condylar process; size of the axis (C2) including the dens; size of the femoral head	Occipital squama gracile without a pronounced external occipital protuberance and nuchal lines, score 1 (female morphology); mental eminence slightly developed, score 2 (female morphology); mandibular condylar process small (female morphology); axis (C2) and dens small (female morphology); femoral head small (female morphology)	Buikstra & Ubelaker, 1994, pp. 15–21; Cavazzuti et al., 2019; Piontek, 1985, pp. 210–215; Strzałko et al., 1973	Probably female	80%	(F)
34	Świbie	252	TCA 36	Zygomatic bone; mandible	Morphology of the frontal process of the zygomatic bone; size of the mandibular condylar process	Frontal process of the zygomatic bone broad with a very pronounced marginal tubercle (male morphology); mandibular condylar process large (male morphology)	Cavazzuti et al., 2019; Piontek, 1985, pp. 210–215; Strzałko et al., 1973	Possibly male	65%	M?
35	Świbie	263	TCA 50	Cranial bones including the supraorbital region; pelvic bone with preserved auricular surface and preauricular sulcus	Morphology of the supraorbital region; morphology of the auricular surface and preauricular sulcus	Supraorbital ridges gracile (female morphology); auricular surface gracile (female morphology); preauricular sulcus present and well developed (female morphology)	Buikstra & Ubelaker, 1994, pp. 15–21; Cavazzuti et al., 2019; Piontek, 1985, pp. 210–215; Strzałko et al., 1973; Wescott, 2015	Possibly female	70%	F?

No.	Site	Grave number	TCA ID	Preserved diagnostic elements	Sex traits assessed	Trait assessment	Method reference	Sex estimation	Confidence of sex assessment (%)*	Final sex assessment
36	Świbie	309	TCA 37	Frontal bone with glabella and supraorbital region; temporal bone with mastoid process; zygomatic bone; distal humerus	Morphology of the glabella, supraorbital margin and supraorbital region; size of the mastoid process; morphology of the frontal process of the zygomatic bone; size of the humeral trochlea	Glabella prominent, score 4 (male morphology); supraorbital margin, score 3 (intermediate morphology); supraorbital ridges pronounced (male morphology); mastoid process large, score 4 (male morphology); frontal process of the zygomatic bone broad (male morphology); humeral trochlea broad (male morphology)	Buikstra & Ubelaker, 1994, pp. 15–21; Cavazzuti et al., 2019; Piontek, 1985, pp. 210–215; Strzałko et al., 1973	Probably male	80%	(M)
37	Świbie	365	TCA 62	Occipital bone; mandible; pelvic bone with preserved auricular surface	Morphology of the nuchal lines; size of the mandibular condylar process; overall robusticity of the mandible; morphology of the auricular surface and presence of the preauricular sulcus	Nuchal lines pronounced, score 4 (male morphology); mandibular condylar process large (male morphology); mandible robust (male morphology); auricular surface without a preauricular sulcus (male morphology)	Buikstra & Ubelaker, 1994, pp. 15–21; Cavazzuti et al., 2019; Piontek, 1985, pp. 210–215; Strzałko et al., 1973; Wescott, 2015	Probably male	80%	(M)
38	Świbie	371	TCA 38	Occipital bone; zygomatic bone; mandible; axis (C2)	Morphology of the occipital squama and nuchal lines; morphology of the frontal process of the zygomatic bone; morphology of the mandibular angle and masseteric tuberosity; size of the odontoid process of the axis (C2)	Occipital squama gracile without pronounced nuchal lines (female morphology); frontal process of the zygomatic bone very narrow without a marginal tubercle (female morphology); mandibular angle gracile without a pronounced masseteric tuberosity (female morphology); odontoid process small (female morphology)	Buikstra & Ubelaker, 1994, pp. 15–21; Cavazzuti et al., 2019; Piontek, 1985, pp. 210–215; Strzałko et al., 1973	Possibly female	70%	F?
39	Świbie	421	TCA 49	Fragments of cranial bones, including the frontal bone and occipital squama	Morphology of the supraorbital margin and supraorbital region; morphology of the external occipital protuberance and nuchal lines	Supraorbital margin sharp, score 1 (female morphology); supraorbital ridge gracile (female morphology); external occipital protuberance and nuchal lines gracile, score 2 (female morphology)	Buikstra & Ubelaker, 1994, pp. 15–21; Piontek, 1985, pp. 210–215; Strzałko et al., 1973	Possibly female	65%	F?
40	Świbie	424	TCA 59	Mandible and left zygomatic bone	Morphology of the mental eminence and zygomatic bone	Mental eminence small, score 2 (female morphology); left zygomatic bone small (female morphology); frontal process of the left zygomatic bone narrow (female morphology)	Buikstra & Ubelaker, 1994, pp. 15–21; Piontek, 1985, pp. 210–215; Strzałko et al., 1973	Possibly female	65%	F?
41	Świbie	427	TCA 48	Fragment of the occipital squama	Morphology of the nuchal crest	External occipital protuberance pronounced, score 4 (male morphology)	Buikstra & Ubelaker, 1994, pp. 15–21; Piontek, 1985, pp. 210–215; Strzałko et al., 1973	Possibly male	60%	not determined
42	Świbie	429	TCA 53	Sexually dimorphic elements not preserved	None	Not applicable	Not applicable	not determined	Not applicable	not determined; subadult individual
43	Świbie	439	TCA 39	Frontal bone	Morphology of the glabella	Glabella gracile, score 1 (female morphology)	Not applicable	not determined	Not applicable	not determined; subadult individual
44	Świbie	458	TCA 40	Occipital bone; mandible; clavicle with preserved sternal end	Morphology of the nuchal lines; size of the mandibular condylar process; size of the sternal end of the clavicle	Nuchal lines gracile and weakly expressed (female morphology); mandibular condylar process small (female morphology); sternal end of the clavicle very small (female morphology)	Buikstra & Ubelaker, 1994, pp. 15–21; Cavazzuti et al., 2019; Piontek, 1985, pp. 210–215; Strzałko et al., 1973	Possibly female	65%	F?
45	Świbie	543	TCA 57	Sexually dimorphic elements not preserved	None	Not applicable	Not applicable	not determined	Not applicable	not determined; subadult individual
46	Świbie	558	TCA 41	Frontal bone; mandible	Morphology of the glabella and supraorbital region; morphology of the mandibular angle and masseteric tuberosity	Supraorbital ridges gracile (female morphology), glabella gracile, score 2 (female morphology); left mandibular angle gracile without a pronounced masseteric tuberosity and without a masseteric fossa (female morphology)	Buikstra & Ubelaker, 1994, pp. 15–21; Piontek, 1985, pp. 210–215; Strzałko et al., 1973	Possibly female	70%	F?
47	Świbie	562	TCA 54	Cranial bones including the frontal bone and supraorbital region	Morphology of the glabella, supraorbital ridges and supraorbital margins	Glabella pronounced, score 4 (male morphology); supraorbital ridges pronounced (male morphology); supraorbital margins rounded, score 4 (male morphology)	Buikstra & Ubelaker, 1994, pp. 15–21; Piontek, 1985, pp. 210–215; Strzałko et al., 1973	Possibly male	70%	M?
48	Wrocław-Żerniki	11	TCA 79	Fragments of cranial bones including the frontal bone and zygomatic bones; fragments of the mandible; fragment of the axis (C2); fragments of the humerus and radius	Morphology of the glabella; morphology of the zygomatic bones and presence/absence of the marginal tubercle; size of the mandibular condylar process; size of the odontoid process (dens) of the axis; size of the humeral and radial heads	Glabella gracile, score 2 (female morphology); zygomatic bones small, marginal tubercle absent (female morphology); mandibular condylar process small (female morphology); odontoid process (dens) of the axis small (female morphology); humeral head small (female morphology); radial head small (female morphology)	Buikstra & Ubelaker, 1994, pp. 15–21; Cavazzuti et al., 2019; Piontek, 1985, pp. 210–215; Strzałko et al., 1973	Probably female	75%	(F)

No.	Site	Grave number	TCA ID	Preserved diagnostic elements	Sex traits assessed	Trait assessment	Method reference	Sex estimation	Confidence of sex assessment (%)*	Final sex assessment
49	Wrocław-Żerniki	18	TCA 80	Fragments of the occipital bone with the external occipital protuberance and nuchal lines; fragment of the frontal bone; fragments of the mandible; fragment of the axis (C2)	Morphology of the external occipital protuberance and nuchal lines; morphology of the frontal bone and supraorbital region; size of the mandibular condylar process; size of the odontoid process (dens) of the axis	Frontal bone smooth, with delicate supraorbital ridges (female morphology); glabella not preserved; external occipital protuberance and nuchal lines moderately developed, score 2–3 (female-leaning morphology); condylar process of the mandible small (female morphology); odontoid process (dens) of the axis small (female morphology)	Buikstra & Ubelaker, 1994, pp. 15–21; Cavazzuti et al., 2019; Piontek, 1985, pp. 210–215; Strzałko et al., 1973	Possibly female	60%	F?
50	Wrocław-Żerniki	52	TCA 81	Fragments of cranial bones including the frontal bone; fragment of the axis (C2)	Morphology of the glabella; size of the odontoid process (dens) of the axis	Glabella moderately developed, score 3–4 (male-leaning morphology); odontoid process (dens) of the axis large (male morphology)	Buikstra & Ubelaker, 1994, pp. 15–21; Cavazzuti et al., 2019; Piontek, 1985, pp. 210–215; Strzałko et al., 1973	Possibly male	60%	M?
51	Wtórek	514	TCA 82	Fragments of the mandible including the mental region and condylar process; fragment of the axis (C2)	Morphology of the mental eminence; morphology of the mandibular condylar process; morphology and robusticity of the axis (C2)	Mental eminence pronounced, score 4 (male morphology); mandibular condylar process robust (male morphology); axis (C2) robust (male morphology)	Buikstra & Ubelaker, 1994, pp. 15–21; Cavazzuti et al., 2019; Piontek, 1985, pp. 210–215; Strzałko et al., 1973	Possibly male	60%	M?
52	Wtórek	526	TCA 42	Fragments of cranial bones including the frontal and temporal regions; fragments of the mandible; fragments of the humerus and radius; fragments of pelvic bones including the auricular surface; fragment of the femur	Morphology of the glabella; morphology of the mastoid process; morphology of the mandibular condylar process and mandibular angles; morphology of the mental eminence; size of the humeral trochlea and radial head; morphology of the auricular surface of the ilium; size of the femoral head	Glabella gracile, score 1 (female morphology); mastoid process small, score 2 (female morphology); mandibular condylar process small (female morphology); mental eminence, score 2 (female morphology); mandibular angles with marked muscular attachments (male-leaning morphology); humeral trochlea small (female morphology); radial head small (female morphology); auricular surface of the ilium (female morphology); femoral head small (female morphology)	Buikstra & Ubelaker, 1994, pp. 15–21; Cavazzuti et al., 2019; Piontek, 1985, pp. 210–215; Strzałko et al., 1973; Wescott, 2015	Probably female	75%	(F)
53	Wtórek	542	TCA 83	Sexually dimorphic elements not preserved	None	Not applicable	Not applicable	not determined	Not applicable	not determined
54	Wtórek	556	TCA 84	Fragments of cranial bones; fragment of the axis (C2); fragment of the pelvic bone	Morphology of the orbital margins; morphology and robusticity of the occipital squama (including the external occipital protuberance and nuchal lines); robusticity of the mastoid process; size of the zygomatic bones; size of the axis (C2); morphology of the pelvic bone, including the auricular surface and presence/absence of the preauricular sulcus	Occipital squama robust, external occipital protuberance prominent, nuchal lines well developed, score 4 (male morphology); orbital margins rounded, score 4 (male morphology); mastoid process robust, score 4 (male morphology); zygomatic bones large (male morphology); axis (C2) large and robust (male morphology); fragment of the iliac bone with auricular surface preserved, greater sciatic notch not preserved; absence of a preauricular sulcus (male morphology)	Buikstra & Ubelaker, 1994, pp. 15–21; Cavazzuti et al., 2019; Piontek, 1985, pp. 210–215; Strzałko et al., 1973; Wescott, 2015	Probably male	75%	(M)
55	Wtórek	558	TCA 85	Fragments of cranial bones including the occipital bone and mandible; fragment of the radius	Morphology of the external occipital protuberance; morphology of the mental eminence; size of the radial head	External occipital protuberance pronounced, score 4 (male morphology); mental eminence pronounced, score 4 (male morphology); radial head large (male morphology)	Buikstra & Ubelaker, 1994, pp. 15–21; Cavazzuti et al., 2019; Piontek, 1985, pp. 210–215; Strzałko et al., 1973	Possibly male	60%	M?
56	Wtórek	578	TCA 43	Sexually dimorphic elements not preserved	None	Not applicable	Not applicable	nd	Not applicable	not determined
57	Wtórek	582	TCA 86	Fragments of cranial bones including the frontal and zygomatic bones; fragments of the mandible with preserved mental region and mandibular angles	Morphology of the glabella; morphology of the supraorbital margin and supraorbital ridges; morphology of the mental eminence; morphology of the mandibular angle, including masseteric tuberosity and masseteric fossa; size and morphology of the zygomatic bones, including the presence of marginal tubercles	Glabella moderately developed, score 3–4 (male-leaning morphology); supraorbital margin score 3 (intermediate morphology); supraorbital ridges pronounced (male morphology); mental eminence pronounced, score 4 (male morphology); mandibular angle robust, with marked masseteric tuberosity and masseteric fossa (male morphology); zygomatic bones large, with marginal tubercles present on the frontal processes (male morphology)	Buikstra & Ubelaker, 1994, pp. 15–21; Cavazzuti et al., 2019; Piontek, 1985, pp. 210–215; Strzałko et al., 1973	Possibly male	70%	M?
58	Wtórek	740	TCA 87	Fragments of cranial bones including the frontal bone; fragments of the zygomatic bones; fragments of the mandible with preserved condylar processes	Morphology of the glabella; size of the mandibular condylar processes; size and morphology of the zygomatic bones, including the presence/absence of the marginal tubercle	Glabella strongly developed, score 4–5 (male morphology); mandibular condylar processes large (male morphology); zygomatic bones large, with the marginal tubercle present (male morphology)	Buikstra & Ubelaker, 1994, pp. 15–21; Cavazzuti et al., 2019; Piontek, 1985, pp. 210–215; Strzałko et al., 1973	Possibly male	70%	M?
59	Wtórek	795	TCA 88	Fragments of cranial bones including the frontal bone; fragment of the occipital squama; left zygomatic bone with preserved frontal process	Morphology of the supraorbital ridges; morphology of the occipital squama, including the external occipital protuberance and nuchal lines; size and morphology of the zygomatic bone, including the frontal process.	Supraorbital margins weakly developed (female morphology); occipital squama smooth, without pronounced nuchal lines or external occipital protuberance (female morphology); left zygomatic bone small, with a narrow frontal process (female morphology)	Buikstra & Ubelaker, 1994, pp. 15–21; Cavazzuti et al., 2019; Piontek, 1985, pp. 210–215; Strzałko et al., 1973	Possibly female	60%	F?

No.	Site	Grave number	TCA ID	Preserved diagnostic elements	Sex traits assessed	Trait assessment	Method reference	Sex estimation	Confidence of sex assessment (%)*	Final sex assessment
60	Wtórek	798	TCA 89	Fragments of cranial bones including the frontal bone and occipital squama; right zygomatic bone with preserved frontal process; fragments of the mandible with the condylar process	Morphology of the supraorbital ridges; morphology of the supraorbital margin; morphology of the glabella; morphology of the external occipital protuberance; morphology of the zygomatic bone, including the frontal process; size of the mandibular condylar process	Supraorbital margins weakly developed (female morphology); supraorbital margin sharp, score 1 (female morphology); glabella smooth, score 1 (female morphology); external occipital protuberance smooth, score 1–2 (female morphology); frontal process of the right zygomatic bone narrow (female morphology); mandibular condylar process small (female morphology)	Buikstra & Ubelaker, 1994, pp. 15–21; Cavazzuti et al., 2019; Piontek, 1985, pp. 210–215; Strzałko et al., 1973	Probably female	80%	(F)
61	Wtórek	819	TCA 90	Fragments of the occipital bone with preserved nuchal lines; left zygomatic bone; proximal epiphysis of the radius	Morphology of the nuchal lines; size and morphology of the left zygomatic bone, including the presence of the marginal tubercle; size of the radial head	Nuchal lines pronounced, score 4 (male morphology); left zygomatic bone very large, with the marginal tubercle present (male morphology); radial head large (male morphology)	Buikstra & Ubelaker, 1994, pp. 15–21; Cavazzuti et al., 2019; Piontek, 1985, pp. 210–215; Strzałko et al., 1973	Possibly male	70%	M?
62	Wtórek	828	TCA 91	Fragments of cranial bones and the mandible	Morphology of the frontal bone and the mental eminence	Glabella gracile, score 1 (female morphology); mental eminence small, score 2 (female morphology)	Buikstra & Ubelaker, 1994, pp. 15–21; Piontek, 1985, pp. 210–215; Strzałko et al., 1973	Possibly female	60%	F?

* Confidence values reflect the reliability of sex estimation based on the number and diagnostic value of the evaluated skeletal traits according to the applied osteological methods and do not represent a statistical posterior probability.

Table S5. Variation of the AEFC thickness (AEFCt) as well as the number (ILSc) and width (ILSw) of ILS according to tooth root division into apical, middle and cervical part.

	AEFCt			ILSc			ILSw		
Tooth root part	Apical part	Middle part	Cervical part	Apical part	Middle part	Cervical part	Apical part	Middle part	Cervical part
N	56	66	80	56	66	80	56	66	80
Min	8,21	9,66	10,31	4,00	5,00	5,50	1,09	1,49	1,47
Max	145,52	124,40	123,03	40,00	38,00	35,90	5,98	5,31	4,87
Mean	52,17	54,79	57,59	16,62	16,80	17,71	3,16	3,26	3,32
Stand. dev.	28,810	24,608	22,884	7,767	6,841	6,455	1,085	0,863	0,868
Median	53,97	55,16	58,40	15,50	16,75	17,00	3,11	3,17	3,43
25 prcntil	28,78	34,32	43,90	10,13	11,38	13,13	2,26	2,72	2,79
75 prcntil	68,33	71,10	67,31	20,88	20,63	21,50	3,89	3,73	3,85

Table S6. Variation in the AEFC thickness (AEFCt) according to the age category determined by morphological methods.

AEFCt [μm]	Infans I	Infans II	Infans II/ Juvenis	Juvenis	Adultus	Adultus/ Maturus	Maturus	Maturus/ Senils	Adult	Not determined
N	3	4	1	2	29	9	3	2	5	2
Min	12,23	10,04	38,00	29,62	27,08	20,25	32,63	48,08	19,54	21,96
Max	24,83	33,43	-	58,93	130,90	69,83	116,06	143,46	97,93	61,20
Mean	17,08	22,41	-	44,28	56,09	54,82	78,47	95,77	53,13	41,58
Stand. dev	6,780	10,078	-	20,725	18,502	15,170	42,321	67,444	28,984	27,747
Median	14,19	23,09	-	44,28	54,97	59,20	86,71	95,77	54,67	41,58
25 prcntil	12,23	12,35	-	29,62	48,04	47,58	32,63	48,08	28,95	21,96
75 prcntil	24,83	31,80	-	58,93	62,94	65,06	116,06	143,46	76,55	61,20

Table S7. Variation in the number of ILS (ILSc) according to the age category determined by morphological methods.

ILSc	Infans I	Infans II	Infans II/ Juvenis	Juvenis	Adultus	Adultus/ Maturus	Maturus	Maturus/ Senils	Adult	Not determined
N	3	4	1	2	29	9	3	2	5	2
Min	5,30	4,30	11,70	11,80	9,00	12,90	16,80	13,30	8,50	10,80
Max	8,00	9,50	-	27,50	31,20	31,20	34,80	27,30	29,10	22,20
Mean	6,87	7,03	-	19,65	18,65	20,37	27,03	20,30	16,58	16,50
Stand. dev	1,401	2,396	-	11,102	4,656	5,596	9,250	9,899	7,871	8,061
Median	7,30	7,15	-	19,65	18,40	19,30	29,50	20,30	16,20	16,50
25 prcntil	5,30	4,68	-	11,80	15,75	16,30	16,80	13,30	10,05	10,80
75 prcntil	8,00	9,25	-	27,50	21,35	24,55	34,80	27,30	23,30	22,20

Table S8. Variation in the width of ILS (ILSw) according to the age category determined by morphological methods.

ILSw [µm]	Infans I	Infans II	Infans II/ Juvenis	Juvenis	Adultus	Adultus/ Maturus	Maturus	Maturus/ Senils	Adult	Not determined
N	3	4	1	2	29	9	3	2	5	2
Min	1,98	2,40	3,28	2,14	1,89	1,62	1,87	3,97	2,34	2,08
Max	3,10	3,52	-	2,66	4,11	4,21	4,09	4,21	3,49	2,89
Mean	2,48	3,12	-	2,40	3,18	3,18	2,82	4,09	3,08	2,49
Stand. dev	0,571	0,498	-	0,368	0,626	0,880	1,145	0,170	0,477	0,573
Median	2,35	3,28	-	2,40	3,23	3,22	2,49	4,09	3,34	2,49
25 prcntil	1,98	2,60	-	2,14	2,62	2,42	1,87	3,97	2,60	2,08
75 prcntil	3,10	3,48	-	2,66	3,66	3,94	4,09	4,21	3,43	2,89

Table S9. The width of single ILS (ILSw) divided according to the morphological sex, taking into account the analysis of 202 slides and mean values for 60 teeth.

ILSw [μm]	Female	Male	nd	Total	Female	Male	nd	Total
N	99	68	35	202	28	19	13	60
Min	1,09	1,70	1,49	1,09	1,62	2,46	1,98	1,62
Max	5,84	5,98	4,04	5,98	4,10	4,21	3,52	4,21
Mean	3,16	3,61	2,85	3,21	3,06	3,36	2,80	3,10
Stand. dev	0,956	0,854	0,768	0,915	0,756	0,592	0,563	0,689
Median	3,16	3,55	2,86	3,19	3,12	3,36	2,89	3,16
25 prcntil	2,33	3,07	2,25	2,57	2,52	2,86	2,25	2,52
75 prcntil	3,76	4,21	3,62	3,77	3,70	3,78	3,33	3,64

Table S10. The ILSw determined on the basis of analyses of 202 slides with respect to particular geographical regions.

ILSw [μm]	South Greater Poland Lowland	Silesian Lowlands	Silesian Upland
N	35	62	105
Min	1,77	1,09	2,25
Max	5,84	4,13	5,98
Mean	3,38	2,45	3,69
Stand. dev	1,076	0,664	0,672
Median	3,22	2,34	3,67
25 prcntil	2,58	1,885	3,17
75 prcntil	4,03	2,98	4,11

Table S11. The ILSw determined on the basis of analyses of 60 teeth with respect to particular geographical regions.

ILSw [µm]	South Greater Poland Lowland	Silesian Lowlands	Silesian Upland
N	13	21	26
Min	1,95	1,32	2,29
Max	4,21	3,13	4,07
Mean	3,16	2,29	3,32
Stand. dev	0,663	0,479	0,408
Median	3,36	2,36	3,29
25 prcntil	2,62	1,98	3,10
75 prcntil	3,69	2,66	3,52

SI References:

1. Großkopf, B. Individualaltersbestimmung mit Hilfe von Zuwachsringen im Zement bodengelagerter menschlicher Zähne. *Zeitschrift für Rechtsmedizin* **103**, 351–359 (1990).

2. Jankauskas, R., Barakauskas, S. & Bojarun, R. Incremental lines of dental cementum in biological age estimation. *HOMO* **52**, 59–71 (2001).

3. Naji, S. *et al.* Cementochronology, to cut or not to cut? *International Journal of Paleopathology* **15**, 113–119 (2016).

4. Wittwer-Backofen, U., Gampe, J. & Vaupel, J. W. Tooth cementum annulation for age estimation: Results from a large known-age validation study. *American Journal of Physical Anthropology: The Official Publication of the American Association of Physical Anthropologists* **123**, 119–129 (2004).

5. Wittwer-Backofen, U. & Buba, H. Age estimation by tooth cementum annulation: Perspectives of a new validation study. in *Paleodemography: Age Distributions from Skeletal Samples* (eds Hoppa & Vaupel) 107–128 (Cambridge University Press., Cambridge, 2002).

6. Bosshardt, D. D. & Schroeder, H. E. Cementogenesis reviewed: a comparison between human premolars and rodent molars. *The Anatomical Record: An Official Publication of the American Association of Anatomists* **245**, 267–292 (1996).

7. Bosshardt, D. D. & Selvig, K. A. Dental cementum: the dynamic tissue covering of the root. *Periodontol 2000* **13**, 41–75 (1997).

8. Couoh, L. R. *et al.* Tooth acellular extrinsic fibre cementum incremental lines in humans are formed by parallel branched Sharpey's fibres and not by its mineral phase. *Journal of Structural Biology* **216**, 108084 (2024).

9. Black, G. V. *A Study of the Histological Characters of the Periosteum and Peridental Membrane*. (WT Keener, 1887).

10. Magitot, E. *Treatise on Dental Caries*. (Boston, Houghton, Osgood & Co, Boston, 1878).

11. Zander, H. A. & Hürzeler, B. Continuous Cementum Apposition. *Journal of Dental Research* **37**, 1035–1044 (1958).

12. Sergeant, D. E. & Pimlott, D. H. Age determination in moose from sectioned incisor teeth. *The Journal of Wildlife Management* **23**, 315–321 (1959).

13. Condon, K., Charles, D. K., Cheverud, J. M. & Buikstra, J. E. Cementum annulation and age determination in Homo sapiens. II. Estimates and accuracy. *Journal of Physical Anthropology* **71**, 321–330 (1986).

14. Stott, G. C., Sis, R. F. & Levy, B. M. Cemental annulation as an age criterion in forensic dentistry. *Journal of Dental Research* **61**, 814–817 (1982).

15. Yamamoto, T., Hasegawa, T., Yamamoto, T., Hongo, H. & Amizuka, N. Histology of human cementum: Its structure, function, and development. *Japanese Dental Science Review* **52**, 63–74 (2016).

16. Gocha, T. P. & Schutkowski, H. Tooth cementum annulation for estimation of age-at-death in thermally altered remains. *Journal of forensic sciences* **58**, S151–S155 (2013).

17. Cavalli, F., Innocenti, D., Črešnar, M. & Vinazza, M. Multidetector computed tomography and micro-excavation of prehistoric urn from Novine/Hoarachkogel. (Slovenia/Austria). in *Archäologische Biographie einer Landschaft an der steirisch-slowenischen Grenze. Ergebnisse des grenzübergereifenden Projekts BorderArch-Steiermark. Arheološka biografija krajine ob meji med avstrijsko Štajersko in Slovenijo. Rezultati čezmejneg aprojekta BorderArch-Steiermark* (eds Črešnar, M., Mele, M., Peitler, K. & Vinazza, M.) vol. Beiheft 6/2015 238–243 (2015).

18. Cobb, E. M. Evaluation of Cementochronology As an Aging Method for Inexperienced Researchers. (Appalachian State University, Boone, NC, USA, 2017).

19. Cerrito, P., Naji, S. & Bromage, T. Optimizing Preparation Protocols and Microscopy for Cementochronology. in *Dental Cementum in Anthropology* (eds Naji, S., Rendu, W. & Gourichon, L.) 189–200 (Cambridge University Press, 2022). doi:10.1017/9781108569507.012.

20. Roksandic, M., Vlak, D., Schillaci, M. A. & Voicu, D. Applicability of tooth cementum annulation to an archaeological population. *American Journal of Physical Anthropology: The Official Publication of the American Association of Physical Anthropologists* **140**, 583–588 (2009).

21. Bertrand, B. Age-at-death estimation by cementochronology—somewhere between indifference and overconfidence. *Forensic Science International* **302**, 109886 (2019).

22. Colard, T., Bertrand, B., Naji, S., Delannoy, Y. & Bécart, A. Toward the adoption of cementochronology in forensic context. *International journal of legal medicine* **132**, 1117–1124 (2018).

23. Huffman, M. & Antoine, D. Analysis of Cementum Layers in Archaeological Material. *DAJ* **23**, 67–73 (2018).

24. Bertrand, B. Cémentochronologie: précision et exactitude de l'estimation de l'âge au décès: influence de la taphonomie. (Université du Droit et de la Santé-Lille II, Français, 2017).

25. Grue, H. & Jensen, B. Review of the formation of incremental lines in tooth cementum of terrestrial mammals [age determination, game animal, variation, sex, reproductive cycle, climate, region, condition of the animal]. *Danish Review of Game Biology (Denmark)* **11**, (1979).

26. Klevezal, G. A. & Mina, M. V. Factors determining the character of annual layers in mammalian tooth and bone tissue. *Zhurnal obshchei biologii* **34**, 594–605 (1973).

27. Klevezal, G. A. & Shishlina, N. I. Assessment of the Season of Death of Ancient Human from Cementum Annual Layers. *Journal of Archaeological Science* **28**, 481–486 (2001).

28. Sinha, N., Sahni, P., Hm, J., Kumar, A. & Gujjar, P. Determination of Season of Death using Dental Cementum Annulations: A study in humans. *Int. J. Oral Health Med. Res* **4**, 19–21 (2017).

29. Wedel, V. L. Determination of Season at Death Using Dental Cementum Increment Analysis*†. *Journal of Forensic Sciences* **52**, 1334–1337 (2007).

30. Lieberman, D. E. The Biological Basis for Seasonal Increments in Dental Cementum and Their Application to Archaeological Research. *Journal of Archaeological Science* **21**, 525–539 (1994).

31. Reimers, E. & Nordby, Ø. Relationship between age and tooth cementum layers in Norwegian reindeer. *The Journal of Wildlife Management* 957–961 (1968).

32. Low, W. A. & Cowan, I. E. Age determination of deer by annular structure of dental cementum. *The Journal of Wildlife Management* 466–471 (1963).

33. Stein, T. J. & Corcoran, J. F. Pararadicular cementum deposition as a criterion for age estimation in human beings. *Oral surgery, oral medicine, oral pathology* **77**, 266–270 (1994).

34. Cerrito, P., Bailey, S. E., Hu, B. & Bromage, T. G. Parturitions, menopause and other physiological stressors are recorded in dental cementum microstructure. *Sci Rep* **10**, 5381 (2020).

35. Kagerer, P. & Grupe, G. Age-at-death diagnosis and determination of life-history parameters by incremental lines in human dental cementum as an identification aid. *Forensic Science International* **118**, 75–82 (2001).

36. Kagerer, P. & Grupe, G. On the validity of individual age-at-death diagnosis by incremental line counts in human dental cementum. Technical considerations. *Anthropologischer Anzeiger* 331–342 (2001).

37. Mani-Caplazi, G., Hotz, G., Wittwer-Backofen, U. & Vach, W. Measuring incremental line width and appearance in the tooth cementum of recent and archaeological human teeth to identify irregularities: First insights using a standardized protocol. *International Journal of Paleopathology* **27**, 24–37 (2019).

38. Penezić, K., Porčić, M., Urban, P. K., Wittwer-Backofen, U. & Stefanović, S. Stressful times for women - Increased physiological stress in Neolithic females detected in tooth cementum. *Journal of Archaeological Science* **122**, 105217 (2020).

39. Edinborough, M., Djotunović, I. & Edinborough, K. Tooth cementum annulation: Confounding difficulties remain when inferring life history parameters from archeological tooth samples. *Journal of Archaeological Science* **134**, 105417 (2021).

40. Bertrand, B., Oliveira-Santos, I. & Cunha, E. Cementochronology: a validated but disregarded method for age at death estimation. in *Age Estimation* 169–186 (Elsevier, 2019). doi:10.1016/B978-0-12-814491-6.00012-1.

41. Naji, S. *et al.* Recovery methods for cremated commingled remains: analysis and interpretation of small fragments using a bioarchaeological approach. in *Commingled Human Remains* 33–56 (Elsevier, 2014).

42. Pany-Kucera, D. *et al.* Social Relations, Deprivation and Violence at Schleinbach, Lower Austria. Insights from an Interdisciplinary Analysis of the Early Bronze Age Human Remains. *Archaeologia Austriaca* **104**, 13–27 (2020).

43. Rebay-Salisbury, K. *The Human Body in Early Iron Age Central Europe: Burial Practices and Images of the Hallstatt World.* (Routledge, 2016).

44. Cunha, E. *et al.* The problem of aging human remains and living individuals: A review. *Forensic Science International* **193**, 1–13 (2009).

45. Perrone, V., Gocha, T. P., Randolph-Quinney, P. & Procopio, N. Tooth Cementum Annulation: A Literature Review. *Forensic Sciences* **2**, 516–550 (2022).

46. Stutz, A. J. Polarizing Microscopy Identification of Chemical Diagenesis in Archaeological Cementum. *Journal of Archaeological Science* **29**, 1327–1347 (2002).

47. Gupta, P., Kaur, H., Shankari G S, M., Jawanda, M. K. & Sahi, N. Human age estimation from tooth cementum and dentin. *J Clin Diagn Res* **8**, ZC07-ZC10 (2014).

48. Thete, S. G. *et al.* Cementum thickness as a parameter for age estimation. *International Journal Of Drug Research And Dental Science* **2**, 1–6 (2020).

49. Sequeira, P., Bosshardt, D. D. & Schroeder, H. E. Growth of acellular extrinsic fiber cementum (AEFC) and density of inserting fibers in human premolars of adolescents. *J of Periodontal Research* **27**, 134–142 (1992).

50. Dastmalchi, R., Poison, A., Bouwsma, O. & Proskin, H. Cementum thickness and mesial drift. *J Clinic Periodontology* **17**, 709–713 (1990).

51. Colard, T. *et al.* New Insights on the Composition and the Structure of the Acellular Extrinsic Fiber Cementum by Raman Analysis. *PLoS ONE* **12**, e0174080 (2017).

52. Ten Cate, A. R. *Oral Histology: Development, Structure, and Function*. (Elsevier Academic Press, St. Louis, Missouri, 2018).

53. Stolarczyk, T. *et al. Czernikowice. Cmentarzyska z Epoki Brązu i Wczesnej Epoki Żelaza*. (Muzeum Miedzi w Legnicy, Legnica, 2020).

54. Baron, J., Skolasiński, Ł. & Żur, T. Late Bronze and Early Iron Age cemetery from Wrocław Żerniki. *Sprawozdania Archeologiczne* **62**, 381–411 (2010).

55. Domańska, J. Cmentarzysko kultury łużyckiej w Cieszkowe, pow. Milicz. *Silesia Antiqua* **15**, 133–207 (1973).

56. Hałuszko, A., Krupski, M. & Grześkowiak, M. Potencjał informacyjny tomografii komputerowej, mikromorfologii i chromatografii gazowej w badaniach pochówków ciałopalnych na przykładzie materiałów ze stan. Rolantowice 2. *Silesia Antiqua* **50**, 61–75 (2015).

57. Mackiewicz, M., Madera, P., Łaciak, D. & Hałuszko, A. The unique settlement microregion of the Lusatian Urnfield culture in Łazy (SW Poland): fieldwalking and geophysical survey results. in 187–191 (2023). doi:10.38072/978-3-928794-83-1/p37.

58. Madera, P. „Wyniki ratowniczych badań wykopaliskowych na cmentarzysku ciałopalnym kultury łużyckiej w Łazach, stan. 1, gm. Wińsk ". *Śląskie Sprawozdania Archeologiczne* **41**, 231–246 (1999).

59. Michnik, M. *Cmentarzysko z Wczesnej Epoki Żelaza w Świbiu Na Górnym Śląsku*. vol. 1 (Muzeum w Gliwicach, Wydawnictwo Profil-Archeo, Gliwice, 2022).

60. McKinley, J. I. & Tech, B. In the heat of the pyre: efficiency of oxidation in Romano-British cremations–did it really matter? in *The analysis of burned human remains* (eds Schmidt, C. & Symes, S.) 163–184 (Elsevier, 2008).

61. Malinowski, T. *Katalog Cmentarzysk Ludności Kultury Łużyckiej w Polsce*. vol. 1 (Warszawa, 1961).

62. Domańska, J. & Gołubkow, J. Materiały z cmentarzyska ciałopalnego ludności kultury łużyckiej w Cieszkowie, woj. Wrocław: Część V. *Silesia Antiqua* **21**, 33–67 (1979).

63. Domańska, J. & Gołubkow, J. Materiały z cmentarzyska ciałopalnego ludności kultury łużyckiej w Cieszkowie, pow. Milicz, z badań w 1973 roku: Część I. *Silesia Antiqua* **17**, 79–136 (1975).

64. Domańska, J. & Gołubkow, J. Materiały z cmentarzyska ciałopalnego kultury łużyckiej w Cieszkowe, woj. Wrocław, z badań w 1973 roku: Część II. *Silesia Antiqua* **18**, 77–119 (1976).

65. Domańska, J. & Gołubkow, J. Materiały z cmentarzyska ciałopalnego kultury łużyckiej w Cieszkowie, woj. Wrocław, z badań w 1974 roku: Część III. *Silesia Antiqua* **19**, 103–149 (1977).

66. Domańska, J. & Gołubkow, J. Materiały z cmentarzyska ciałopalnego kultury łużyckiej w Cieszkowie, woj. Wrocław, z badań w 1975 roku: Część IV. *Silesia Antiqua* **20**, 49–86 (1978).

67. Gołubkow, J. & Domańska, J. Badania: Cieszków, pow. Milicz. *Informator Archeologiczny* **8**, 56 (1973).

68. Domańska, J. & Gołubkow, J. Materiały z cmentarzyska ciałopalnego kultury łużyckiej w Cieszkowe, pow. Milicz, z badań w 1973 roku: Część I. *Silesia Antiqua* **17**, 79–136 (1975).

69. Kaletyn, T. Z pradziejów powiatu złotoryjskiego. *Szkice Legnickie* **5**, 43–59 (1969).

70. Kaletyn, T. & Starzyński, A. Czernikowice, pow. Złotoryja. Stanowisko 2. *Informator Archeologiczny* **6**, 60 (1972).

71. Hałuszko, A. & Guziński, M. Application of the lateral angle method for sex determination of cremated individuals from burials of the Lusatian culture cemetery in Czernikowice, Poland. *Anthropological Review* **85**, 63–75 (2022).

72. Kaletyn, T. Odkrycia i badania: Krzyżowice, pow. Wrocław. *Silesia Antiqua* **5**, 278 (1963).

73. Seger, H. Schlesische Fundchronik. *Schlesiens Vorzeit in Bild und Schrift* **7**, 209–248 (1899).

74. Madera, P. Cmentarzysko ciałopalne kultury łużyckiej w Łazach, stan. 1, pow. Wołów, woj. dolnośląskie, w świetle ostatnich badań ratowniczych. in *Wielkie cmentarzyska z epoki brązu i wczesnej epoki żelaza* (ed. Gedl, M.) vol. 5 149–174 (Wydawnictwo Naukowe PWN, Warszawa, 2002).

75. Lasak, I. Odkrycia i badania: Rolantowice, woj. Wrocław. *Silesia Antiqua* **18**, 301–303 (1976).

76. Malinowski, T. *Katalog Cmentarzysk Ludności Kultury Łużyckiej w Polsce*. vol. 2 (Warszawa, 1961).

77. Raschke, G. Rolandsmühle. (1925).

78. Raschke, G. *Das Ende Der Lausitzer Kultur in Schlesien : Inaugural-Dissertation Zur Erlangung Der Doktorwürde Bei Der Hohen Philosphischen Fakultät Der Schlesischen Friedrich-Wilhelms-Universität Zu Breslau*. (Oberschlesische Gesellschaftsdruckerei m.b. H. Ratibor Wilhelmstr. 7-11, Breslau, 1932).

79. Hałuszko, A. Kondycja biologiczna populacji pierwszych rolników i hodowców na Śląsku. in *Pierwsi rolnicy i hodowcy na Śląsku. Dialog interdyscyplinarny* (ed. Furmanek, M.) 257–288 (Wrocław, 2019).

80. *Cmentarzysko z Wczesnej Epoki Żelaza w Świbiu Na Górnym Śląsku*. vol. 2 (Muzeum w Gliwicach, Wydawnictwo Profil-Archeo, Gliwice, 2022).

81. Hałuszko, A. Analizy bioarcheologiczne szczątków kostnych z pochówków szkieletowych i ciałopalnych ze Świbia. in *Cmentarzysko z wczesnej epoki żelaza w Świbiu na Górnym Śląsku* (eds Michnik, M. & Dzięgielewski, K.) vol. 2 152–172 (Muzeum w Gliwicach, Wydawnictwo Profil-Archeo, Gliwice, 2022).

82. Pudełko, E. & Ziąbka, L. Nowe, cenne wykopaliska z okolic Ostrowa Wielkopolskiego. *Rocznik Kaliski* **32**, 213–218 (2006).

83. Hałuszko, A., Kadej, M., Gmyrek, G. & Guziński, M. Let's make a mess, maybe no one will notice. The impact of bioturbation activity on the urn fill condition. *PLoS ONE* **17**, e0274068 (2022).

84. Hunger, H. & Rother, P. Altersbestimmung am Skelett. in *Identifikation* (eds Hunger, H. & Leopold, D.) 162–183 (Springer-Verlag, Berlin, Heidelberg, New York, 1978).

85. Acsádi, G., Nemeskéri, J. & Balás, K. *History of Human Life Span and Mortality*. (Akademiai Kiado Budapest, Budapest, 1970).

86. Bass, W. *Human Osteology: A Laboratory and Field Manual*. (Missouri Archaeological Society, Springfield, 2005).

87. Dokládal, M. Morfologie spálenỳch kostí. Vỳznam pro identifikaci osob. *Vỳznam pro identifikaci osob. Lékařská Fakulta Masarykovy Univerzity v Brně* **113**, 1–187 (1999).

88. Hershkovitz, I. *et al.* Why do we fail in aging the skull from the sagittal suture? *American Journal of Physical Anthropology: The Official Publication of the American Association of Physical Anthropologists* **103**, 393–399 (1997).

89. Meindl, R. S. & Lovejoy, C. O. Ectocranial suture closure: A revised method for the determination of skeletal age at death based on the lateral-anterior sutures. *American journal of physical anthropology* **68**, 57–66 (1985).

90. Masset, C. Age estimation on the basis of cranial sutures. *Age markers in the human skeleton* (1989).

91. Lovejoy, C. O., Meindl, R. S., Pryzbeck, T. R. & Mensforth, R. P. Chronological metamorphosis of the auricular surface of the ilium: a new method for the determination of adult skeletal age at death. *American journal of physical anthropology* **68**, 15–28 (1985).

92. Smith, B. H. Chapter 8: Standards of Human Tooth Formation and Dental Age Assessment. in *Advances in Dental Anthropology* (eds Kelley, M. A. & Larsen, C. S.) 143–168 (Wiley-Liss, New York, 1991).

93. Martin, R. & Knussmann, R. *Anthropologie. Handbuch Der Vergleichenden. Biologie Des Menschen*. vol. 1 (Gustav Fischer Verlag, Stuttgart-New York, 1988).

94. Ubelaker, D. H. *Human Skeletal Remains. Excavation, Analysis, Interpretation*. (Aldine Publishing Company, Chicago, Chicago, 1978).

95. Cunningham, C., Scheuer, L. & Black, S. *Developmental Juvenile Osteology*. (Academic press, 2016).

96. Baker, B. J., Dupras, T. L. & Tocheri, M. W. *The Osteology of Infants and Children*. vol. 12 (Texas A&M University Press, 2005).

97. Herrmann, B., Grupe, G., Hummel, S., Piepenbrink, H. & Schutkowski, H. *Prähistorische Anthropologie: Leitfaden Der Feld-Und Labormethoden*. (Springer-Verlag, 1990).

98. Szilvássy, J. Alterdiagnose am Skelett. in *Anthropologie. Handbuch der vergleichenden Biologie des Menschen* (eds Martin, R. & Knussmann, R.) 421–443 (Gustav Fischer Verlag, Stuttgart-New York, 1988).

99. Buikstra, J. E. & Ubelaker, D. H. *Standards for Data Collection from Human Skeletal Remains*. vol. 44 (Arkansas Archeological Survey, Fayetteville, Arkansas, 1994).

100. White, T. D., Black, M. T. & Folkens, P. A. *Human Osteology*. (ELSEVIER Academic press, Amsterdam-Boston-Heidelberg-London-New York-Oxford-Paris-San Diego-San Francisco-Singapore-Sydney-Tokyo, 2012).

101. Strzałko, J., Piontek, J. & Malinowski, A. Teoretyczno-metodyczne podstawy badań kości z grobów ciałopalnych. *Materiały i Prace Antropologiczne* 179–201 (1973).

102. Piontek, J. *Biologia Populacji Pradziejowych*. (Uniwersytet im. Adama Mickiewicza w Poznaniu, Poznań, 1985).

103. Wescott, D. J. Sexual Dimorphism in Auricular Surface Projection and Postauricular Sulcus Morphology. *Journal of Forensic Sciences* **60**, 679–685 (2015).

104. Cavazzuti, C., Bresadola, B., d'Innocenzo, C., Interlando, S. & Sperduti, A. Towards a new osteometric method for sexing ancient cremated human remains. Analysis of Late Bronze Age and Iron Age samples from Italy with gendered grave goods. *PloS one* **14**, e0209423 (2019).

105. van Vark, G. The investigation of human cremated skeletal material by multivariate statistical methods I. Methodology. *Ossa* **1**, 63–95 (1974).

106. Waltenberger, L. *et al.* Lateral angle: A landmark-based method for the sex estimation in human cremated remains and application to an Austrian prehistoric sample. *American Journal of Biological Anthropology* **184**, e24874 (2024).

107. Bonczarowska, J. H., McWhirter, Z. & Kranioti, E. F. Sexual dimorphism of the lateral angle: Is it really applicable in forensic sex estimation? *Archives of Oral Biology* **124**, 105052 (2021).

108. Akansel, G. *et al.* Gender and the lateral angle of the internal acoustic canal meatus as measured on computerized tomography of the temporal bone. *Forensic Science International* **178 2–3**, 93–5 (2008).

109. Gonçalves, D., Thompson, T. & Cunha, E. Sexual dimorphism of the lateral angle of the internal auditory canal and its potential for sex estimation of burned human skeletal remains. *International Journal of Legal Medicine* **129**, 1183–1186 (2015).

110. Versiani, M. A., Pécora, J. D. & de Sousa-Neto, M. D. Root and Root Canal Morphology of Four-rooted Maxillary Second Molars: A Micro–Computed Tomography Study. *Journal of Endodontics* **38**, 977–982 (2012).

111. Wang, Y.-L. *et al.* A study on the root canal morphology of primary molars by high-resolution computed tomography. *Journal of Dental Sciences* **8**, 321–327 (2013).

112. Fuller, J. L., Denehy, G. E. & Hall, S. A. *Concise Dental Anatomy and Morphology*. (University of Iowa, Publications Dept., Iowa City, Iowa, 2001).

113. Van Beek, G. *Dental Morphology: An Illustrated Guide. Wright*. (Wright, Oxford, 1983).

114. AlQahtani, S. J., Hector, M. P. & Liversidge, H. M. Brief communication: The London atlas of human tooth development and eruption. *Am. J. Phys. Anthropol.* **142**, 481–490 (2010).

115. Plenk, H. The microscopic evaluation of hard tissue implants. *Techniques of biocompatibility testing* **1**, 35–81 (1986).

116. Nicklisch, N., Hinrichs, C., Palaske, L., Vach, W. & Alt, K. W. Variability in human tooth cementum thickness reflecting functional processes. *J of Periodontal Research* **59**, 408–419 (2024).
